# PKC-eta promotes breast cancer metastasis by regulating the Hippo–YAP signaling pathway

**DOI:** 10.1038/s41392-026-02572-0

**Published:** 2026-02-17

**Authors:** Vijayasteltar B. Liju, Kamran Waidha, Amitha Muraleedharan, Divya Ram Jayaram, Hodaya Haimov, Sankar Jagadeeshan, Dinesh Babu Manikandan, Raghda Abu Shareb, Livingstone Nurukurti, Menachem Sklarz, J. Silvio Gutkind, Irit Allon, Ofir Cohen, Moshe Elkabets, Etta Livneh

**Affiliations:** 1 The Shraga Segal Department of Microbiology, Immunology and Genetics, Faculty of Health Sciences, Ben-Gurion University of the Negev, Beer-Sheva, Israel; 2 Centre d’Excellence de Recherche sur les Maladies Orphelines – Fondation Courtois (CERMO-FC) et Département des Sciences biologiques, Université du Québec à Montréal, Montréal, QC, Canada; 3 Department of Medicine, Harvard Medical School, Boston, MA USA; 4 Department of Pharmacology and Moores Cancer Center, University of California, San Diego, La Jolla, CA USA; 5 Faculty of Health Sciences, Ben-Gurion University of the Negev, Beer-Sheva, Israel; 6 Institute of Pathology, Barzilai University Medical Center, Ashqelon, Israel; 7 Faculty of Computer and Information Science, Ben-Gurion University of the Negev, Beer-Sheva, Israel

**Keywords:** Breast cancer, Metastasis, Drug development, Metastasis

## Abstract

Triple-negative breast cancer (TNBC) is an aggressive disease characterized by high metastatic potential and limited treatment options. Protein kinase C-eta (PKCη), an antiapoptotic kinase of the novel PKC subfamily, is associated with poor prognosis in breast cancer patients. Analysis of TNBC tumors revealed that *PRKCH* (PKCη) expression is linked to an epithelial‒mesenchymal transition (EMT) signature, which is indicative of a metastatic phenotype. Using genetic ablation studies, we showed that PKCη promotes metastasis by enhancing EMT and stemness. Notably, compared with those in PKCη-intact tumors, orthotopic xenografts of PKCη-knockout cells in NSG mice resulted in reduced tumor growth and metastasis. Mechanistically, PKCη functions as a negative regulator of the Hippo pathway by activating YAP. PKCη phosphorylates YAP at Ser128, leading to its stabilization and nuclear translocation, which promotes metastasis. We also demonstrated that PKCη negatively regulates AKT, thereby further sustaining the downregulation of the Hippo pathway. Finally, we show that an evolutionarily conserved peptide encoded by an upstream open reading frame (uORF) preceding the PKCη coding sequence functions as a PKCη degrader, activating the Hippo pathway and promoting YAP degradation. Together, our findings reveal a PKCη-driven signaling axis that regulates the Hippo–YAP pathway in TNBC metastasis, highlighting the potential therapeutic vulnerability of this aggressive disease.

## Introduction

Breast cancer (BC) is a major health concern worldwide. It is the most prevalent cancer in women and the leading cause of death, with over 2.3 million new cases and 0.685 million deaths per year.^1,2^ Among the various BC subtypes, triple-negative breast cancer (TNBC) poses the most significant challenge because of its poor prognosis and limited treatment options. Patients with TNBC have an increased risk of metastasis, mainly to the bone, lung, liver, and brain, with 5-year survival rates of only 22%.^3,4^ Therefore, understanding the molecular mechanisms that drive TNBC metastasis is crucial for developing therapies to combat TNBC progression and metastasis.

Epithelial‒mesenchymal transition (EMT) is the process in which cell‒cell adhesion is lost, while fibroblast-like cell properties, including a spindle-shaped morphology, increased motility and invasiveness, cytoskeleton rearrangement, and the ability of cells to disseminate from the primary tumor, are acquired, thus leading to the acquisition of highly invasive traits of cancer cells.^5^ EMT and cancer stem cells (CSCs) are critical drivers of metastasis. Self-renewing CSCs promote tumor recurrence and metastatic dissemination, whereas mesenchymal-like cells sustain the CSC population, thereby facilitating the metastatic cascade.^6,7^ Activation of YAP/TAZ, a transcriptional coactivator, is one of the primary mechanisms that promotes EMT and facilitates the enrichment of CSCs.^8^ YAP and TAZ are key downstream targets of the Hippo pathway, are crucial for cancer progression and metastasis, and are associated with poor prognosis in cancer patients, including those with TNBC.^9^ The Hippo–YAP pathway is an evolutionarily conserved signaling pathway that controls organ size and tissue homeostasis.^10^ Core elements of the Hippo pathway include a kinase cascade comprising MST1/2 and LATS1/2 kinases, in which MST1/2 phosphorylates and activates LATS1/2.^11^ The phosphorylation of YAP/TAZ at specific sites via the Hippo pathway dictates their cytoplasmic retention, degradation, nuclear translocation, and activation.^12^ When Hippo signaling is inhibited, YAP/TAZ translocates into the nucleus, where it interacts with the TEA domain family of transcription factors (TEADs) and activates the transcription of genes that promote cell growth and migration. AKT and PTEN are upstream regulators of the Hippo–YAP pathway. AKT phosphorylation leads to activation of the Hippo pathway by causing MST1 and LATS1 activation, thus inducing YAP phosphorylation at Ser127 and Ser397, which increases YAP cytoplasmic retention and degradation, further suppressing metastatic drive in cancer.^13^ Dephosphorylation of AKT by the PTEN phosphatase prevents YAP inactivation and cytoplasmic retention, allowing YAP to undergo nuclear translocation and promote cancer progression.^13^

G protein-coupled receptors (GPCRs)^14^ are upstream regulators of the Hippo–YAP pathway. Protein kinase C (PKC) enzymes are major downstream effectors of GPCRs.^15^ These findings prompted us to investigate the role of PKCs in regulating the Hippo pathway in BC. PKC family members are crucial signal transducers that regulate a wide range of cellular processes. The ten isoforms in this family function as serine/threonine kinases and are classified as conventional (PKCα, βI, βII, γ), novel (PKCδ, ε, η, θ), or atypical (PKCζ, ι/λ) on the basis of their structural divergence and biochemical properties. PKCs, which are endogenous receptors for tumor-promoting phorbol esters, often exhibit altered expression and/or activity in cancers and play pivotal roles in tumor progression. The different PKC isoforms function through the modulation of EMT and CSC properties via diverse molecular mechanisms.^16–20^ Notably, PKCs also intersect with the Hippo–YAP pathway; conventional and novel PKC isoforms were reported to play opposite roles in Hippo–YAP pathway regulation.^15^

Our studies and others have suggested that PKCη, a signaling and antiapoptotic stress kinase of the novel PKC subfamily, plays a role in BC tumorigenesis.^21,22^ In some studies, elevated levels of PKCη were associated with increased tumor aggressiveness and poor prognosis and were also associated with positive lymph node status.^22–26^ Our recent study revealed that an evolutionarily conserved peptide encoded by a uORF in the 5’ UTR of the PKCη transcript inhibits the kinase activity of PKCη and other novel PKC isoforms,^21^ resulting in reduced tumor formation and metastasis; however, the underlying molecular mechanisms of its role in metastasis have not been elucidated.

In this study, we revealed a new molecular mechanism that regulates EMT and metastasis driven by PKCη in TNBC cells. Using genetic ablation and the evolutionarily designed uORF-encoded peptide (uPEP2), we demonstrated that PKCη promotes metastasis by enhancing the EMT phenotype and stemness and revealed a new role for PKCη in regulating the Hippo–YAP pathway, which contributes to the aggressive phenotype of TNBC.

## Results

### Analysis of breast cancer tumors revealed that PKCη underlies EMT plasticity in TNBC

**Fig. 1:**
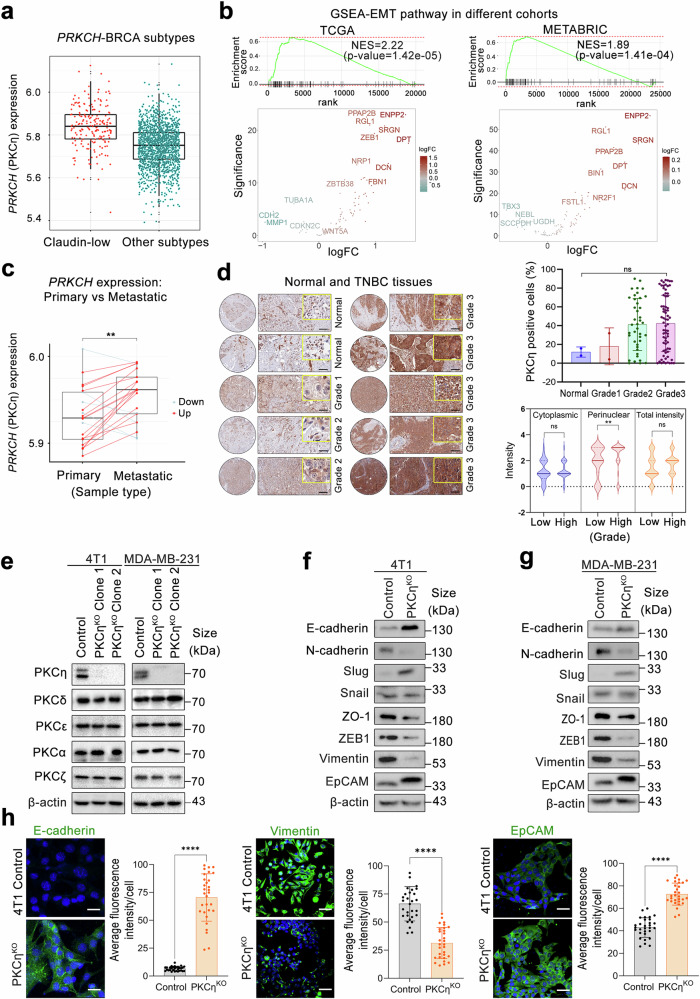
Analysis of BC patient samples revealed an association between *PRKCH* (PKCη) and epithelial‒mesenchymal transition, with ablation of PKCη diminishing EMT in 4T1 and MDA-MB-231 cells. **a** Box plots showing the expression levels of *PRKCH* in BC patients from the METABRIC dataset. Compared with other BRCA subtypes, “claudin-low” (*n* = 218) patients had higher *PRKCH* levels (*n* = 1762), *p* value = 5.21 × 10^−28^. **b** Gene set enrichment analysis (GSEA) revealed significant EMT pathway activation in *PRKCH*-overexpressing patients across several cohorts, including primary BC datasets (METABRIC and TCGA). **c** PKCη expression was significantly higher in metastatic breast tumors than in their matched primary counterparts (*p* = 0.002, paired t test) [PMID:39642223]. **d** Representative immunostaining image showing PKCη expression in TNBC tissue microarray samples. The percentage of PKCη-positive cells tended to increase from low-grade to high-grade TNBC tumors. The perinuclear PKCη intensity significantly increased from low- to high-grade tumors, with no significant difference in the cytoplasmic and total PKCη intensities between low- and high-grade tumors. The scale bar indicates 100 μm. **e** PKCη knockout in 4T1 and MDA-MB-231 cells was verified by western blot analysis, with no change in the expression of the other PKC isoforms. **f**, **g** Western blot analysis of EMT markers was performed using CRISPR control and PKCη^KO^ clones derived from 4T1 and MDA-MB-231 cells, which presented reduced expression of EMT markers. **h** Representative fluorescence images and quantification of E-cadherin, vimentin, and EpCAM expression in 4T1 cells obtained from CRISPR control and PKCη^KO strains^. E-cadherin, vimentin, and EpCAM were stained green, and the nuclei were counterstained with DAPI (blue). The scale bar indicates 50 μm. The data are presented as the means ± SEMs. Statistical significance was determined via two-way ANOVA, where *P < 0.05, **P < 0.01, ***P < 0.001, and ****P < 0.0001

To investigate the role of the *PRKCH* gene, which encodes PKCη, in BC patients, we evaluated and compared its expression levels across different intrinsic subtypes of BC via the METABRIC and TCGA datasets.^27^ In both the TCGA and METABRIC datasets, PRKCH expression was significantly elevated in claudin-low tumors compared with other intrinsic subtypes, including HER2-enriched, luminal A, luminal B, and basal subtypes (Fig. 1a and Supplementary Fig. 1a). However, this observation was not confirmed when tumors were stratified simply by TNBC status or non-TNBC status (Supplementary Fig. 1b). Importantly, in the TNBC patients in the METABRIC cohort, PKCη expression remained significantly higher in the claudin-low subset than in the nonclaudin-low subset of TNBC tumors (Supplementary Fig. 1c). Together, these findings reinforce the conclusion that PKCη is more strongly associated with the claudin-low molecular phenotype than with TNBC, which is defined solely by receptor status. This finding suggests an association between *PRKCH* and metastasis, as the claudin-low subtype of BC is characterized by an enriched stem cell-like population and increased EMT activity.^28,29^ The elevated *PRKCH* expression in the claudin-low subtype further demonstrates a plausible key role of *PRKCH* in more aggressive cancer phenotypes, such as TNBC.

Our subsequent analysis directly characterized the transcriptional state associated with *PRKCH* expression via whole-transcriptome data. We partitioned BC samples on the basis of their *PRKCH* expression levels (top 80% and bottom 20% of tumors; differentially expressed genes are specified in Supplementary Tables 1 and 2) and identified the expression signatures associated with high versus low *PRKCH* levels via gene set enrichment analysis (GSEA; see “Materials and methods”). Our results revealed that *PRKCH* levels were significantly correlated with EMT in patients with BC across multiple cohorts, including TCGA^30,31^ (*n* = 956), METABRIC^27^ (*n* = 1980), POG570^32^ (*n* = 148), and MET500^33^ (*n* = 92), with NESs of 1.89, 2.22, 4.34, and 2.23 and *p* values of 1.41 × 10⁻⁴, 1.42 × 10⁻⁵, 3.12 × 10⁻², and 1.86 × 10⁻⁵, respectively (Fig. 1b and Supplementary Fig. 2a, b). Critically, the association between high *PRKCH* expression and the EMT signature persisted when different stratification approaches were used (top and bottom 10% and 25%), confirming the reliability of this molecular link (Supplementary Fig. 3a–h) and further validating several significant signatures enriched in high-*PRKCH* tumors, including hallmark EMT, TNBC EMT, and others (Supplementary Tables 3 and 4). High *PRKCH* expression was associated with significantly elevated expression of mesenchymal markers (*CXCL12, SFRP4, CXCR4, VIM, PDGFRA, COL6A3, LUM, MMP2,* and *ZEB1*), indicating enhanced promesenchymal characteristics (Supplementary Fig. 4a). Conversely, low *PRKCH* expression is associated with increased expression of proepithelial markers (*EPCAM, ESR1, KRT19, KRT8, GATA3, KRT18, OCLN, FOXA1*, and *CDH1*), reflecting preserved epithelial features in BC (Supplementary Fig. 4b).

Next, we extended our analysis to include patient-matched primary and metastatic tumor samples via the JNCI dataset [PMID: 39642223].^34^ We observed a significant trend of increased *PRKCH* expression in metastatic samples compared with their matched primary tumors (*p* = 0.002, t test, Fig. 1c). Together, these findings demonstrate that *PRKCH* expression is not only subtype specific (claudin-low) but also increases during tumor progression and metastasis, highlighting its potential clinical relevance.

To validate the correlation between PKCη expression levels and BC malignancy in clinical samples, we performed immunohistochemistry (IHC) analysis via a human BC tissue microarray (TMA) comprising normal breast tissue and TNBC samples of varying histological grades and clinical stages (Supplementary Fig. 5a). The percentage of PKCη-positive cells tended to increase with increasing tumor grade and clinical stage and was predominantly greater in stage IIIB and IIIC tumors than in early-stage tumors (Fig. 1d and Supplementary Fig. 5b). Subcellular localization analysis revealed that perinuclear PKCη expression, indicating its activation, increased significantly in high-grade and advanced clinical stages of TNBC.^35–37^

To elucidate the functional impact of PKCη in the context of metastatic TNBC, a targeted approach involving CRISPR/Cas9 genome editing was used to achieve knockout (KO) of the *PRKCH* gene in the murine TNBC cell line 4T1 and the human cell line MDA-MB-231 (Supplementary Fig. 6). We selected these two cell lines owing to their ‘claudin-low’ expression and in vivo metastatic potential.^28,38^ Two clones were selected for each cell line, which clearly showed a specific knockout of PKCη without disrupting the expression of other PKC isoforms present in these cells (Fig. 1e and Supplementary Fig. 6b, c).

Deletion of PKCη in 4T1 and MDA-MB-231 cells resulted in a decrease in the expression of the EMT transcription factor ZEB1 compared with that in control cells, whereas Slug was slightly increased, and the expression of Snail remained unaffected by the absence of PKCη (Fig. 1f, g and Supplementary Fig. 7). Moreover, deletion of PKCη increased the expression of the epithelial markers E-cadherin and EpCAM and downregulated vimentin and N-cadherin expression (Fig. 1f–h). Further support for the role of PKCη in EMT stemmed from the re-expression of PKCη in MDA-MB-231 PKCη-ablated cells, which restored the expression of EMT markers (Supplementary Fig. 8c). Conversely, PKCη knockdown via siRNA suppressed the expression of EMT markers (Supplementary Fig. 9a). To evaluate whether PKCη is sufficient to drive EMT in nonaggressive BC cells, we performed gain-of-function experiments by overexpressing PKCη in MCF7 cells, a luminal nonaggressive BC cell line. Western blot analysis of EMT markers revealed that PKCη overexpression induced a mesenchymal phenotype, with upregulation of mesenchymal markers such as N-cadherin, Slug, Snail, vimentin, and ZO-1 (Supplementary Fig. 10a). Conversely, the epithelial marker E-cadherin was decreased following PKCη overexpression in MCF7 cells. Compared with that of control cells, PKCη overexpression significantly increased the invasive ability of MCF7 cells (Supplementary Fig. 10b). These findings indicate that PKCη is sufficient to drive EMT in luminal nonaggressive MCF7 cells, complementing our PKCη loss-of-function studies in TNBC cell lines.

### PKCη depletion impairs the migration, invasion, and stemness of TNBC cells

**Fig. 2:**
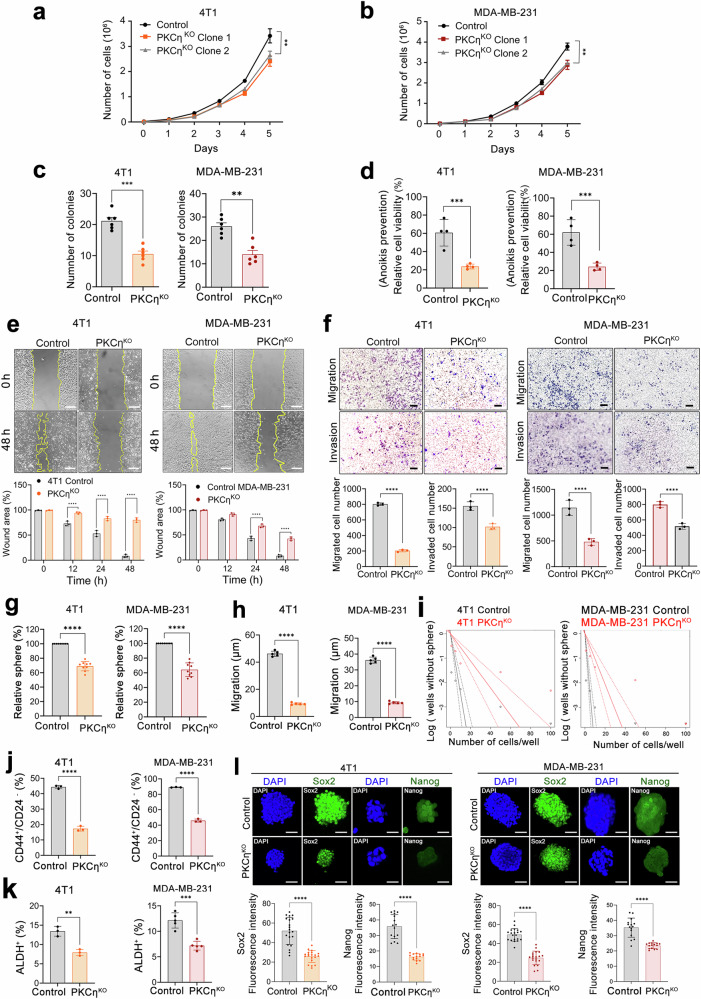
PKCη mediates the proliferation, spheroid formation, migration, invasion, and stemness of TNBC cells. **a**, **b** PKCη^KO^ cells moderately inhibited the proliferation of both the 4T1 and MDA-MB-231 cell lines. **c** PKCη^KO^ reduced the soft agar colony-forming ability of 4T1 and MDA-MB-231 cells. **d** The graph shows that 4T1 and MDA-MB-231 cells were more resistant to anoikis than were PKCη^KO^ TNBC cells. Cell viability assays were performed after 48 h of suspension culture. **e** PKCη enhanced the migration of 4T1 and MDA-MB-231 cells in wound-healing experiments. The area devoid of migratory cells (marked in yellow) was measured and expressed as the mean ± SD (*n* = 3). The scale bar indicates 100 μm. **f** Compared with control cells, 4T1 and MDA-MB-231 PKCη^KO^ cells presented reduced migration and invasion abilities. Cell migration was evaluated via Boyden chamber assays, and cell invasion was assessed via Matrigel-coated Boyden chamber assays. The scale bar indicates 50 μm. **g** Graphical representation indicating a significant reduction in both the size and number of spheres in PKCη^KO^ cells (*n* = 9). TNBC cells were cultured in special media under sphere-forming conditions for seven days. **h** PKCη knockout resulted in reduced sphere cell migration through Matrigel. The migration zones were quantified after 24 h, and the results are presented in the accompanying graph (*n* = 5). **i** An extreme limiting dilution assay (ELDA; https://bioinf.wehi.edu.au/software/elda/) was used to compare stem cell frequencies between the control and PKCη^KO^ clones of 4T1 and MDA-MB-231 cells (*n* = 20). The frequency of the sphere-forming ability of PKCη in TNBC cells is shown for 4T1 (left panel) and MDA-MB-231 (right panel) cells. **j** Flow cytometry analysis showing decreased CD44^+^/CD24^-^ expression in PKCη^KO^ cells. **k** Flow cytometry analysis of ALDEFLUOR activity revealed decreased stem cell activity in PKCη^KO^ cells compared with control 4T1 and MDA-MB-231 cells. **l** Representative images of immunofluorescence staining for the stem cell markers Sox2 and Nanog in 4T1 and MDA-MB-231 cells. Sox2 is predominantly localized in the nucleus. PKCη^KO^ cells presented reduced expression of Sox2 and Nanog. Nuclear staining was performed using DAPI (blue). The scale bar indicates 50 μm. A quantitative analysis of these images is presented as the average fluorescence intensity/cell. Statistical significance was determined via two-way ANOVA, where *P < 0.05, **P < 0.01, ***P < 0.001, and ****P < 0.0001

Depletion of PKCη in the 4T1 and MDA-MB-231 cell lines resulted in only a minimal reduction (~15%) in cell proliferation compared with that in the scrambled control cells (Fig. 2a, b). However, PKCη depletion had a profound effect on cellular processes related to metastasis. A significant reduction in the number and size of colonies formed was observed in the 4T1 and MDA-MB-231 cell lines following PKCη deletion (Fig. 2c and Supplementary Fig. 11a, b). In addition, these cells presented lower resistance to anoikis (Fig. 2d), lower wound closure rates (Fig. 2e), and significant inhibition of migration and invasion than did the control cells (Fig. 2f). Τhe re-expression of PKCη in MDA-MB-231 cells restored the migratory and invasive capacities of PKCη^KO^ cells (Supplementary Fig. 8a, b). Furthermore, acute silencing of PKCη via siRNA in both 4T1 and MDA-MB-231 cells also reduced their migration and invasion abilities (Supplementary Fig. 9b, c).

As EMT is known to be associated with an increased incidence of CSCs, we first explored the association of *PRKCH* with CSCs in public gene expression datasets, followed by the role of PKCη in CSCs in our TNBC cell lines.^39^ Analysis of Gene Expression Omnibus (GEO) datasets revealed a significant increase in *PRKCH* expression in the stem cell-enriched MDA-MB-231 cell line (Supplementary Fig. 12a, b). Moreover, MDA-MB-231 cells enriched with the CSC marker OCT4 also presented increased *PRKCH* levels (GSE86861) (Supplementary Fig. 12a). In addition, *PRKCH* levels were significantly higher in MDA-MB-231 large spheroids than in their nonspheroid counterparts (GSE235703) (Supplementary Fig. 12b). Compared with control cells, PKCη^KO^ cells presented a significant reduction in the number of spheres (Fig. 2g and Supplementary Fig. 12c). Interestingly, compared with control spheroids, TNBC spheroids devoid of PKCη embedded in Matrigel significantly inhibited spheroid cell migration (Fig. 2h and Supplementary Fig. 12d), indicating reduced invasiveness. Limiting dilution assays (LDAs) revealed a decreased ability of 4T1 and MDA-MB-231 PKCη^KO^ cells to develop spheres within one week (Fig. 2i). Among the latter cells, the CSC population, characterized by CD44^high^ CD24^low^ and ALDH^+^ cells, was significantly lower (Fig. 2j, k and Supplementary Fig. 12e, f). Finally, the expression of the CSC markers Nanog and Sox2 was significantly greater in control cells than in PKCη^KO^ cells (Fig. 2l). Overall, our results indicate that PKCη plays a critical role in promoting stemness and invasion.

### PKCη promotes tumor progression and metastasis in mice

**Fig. 3:**
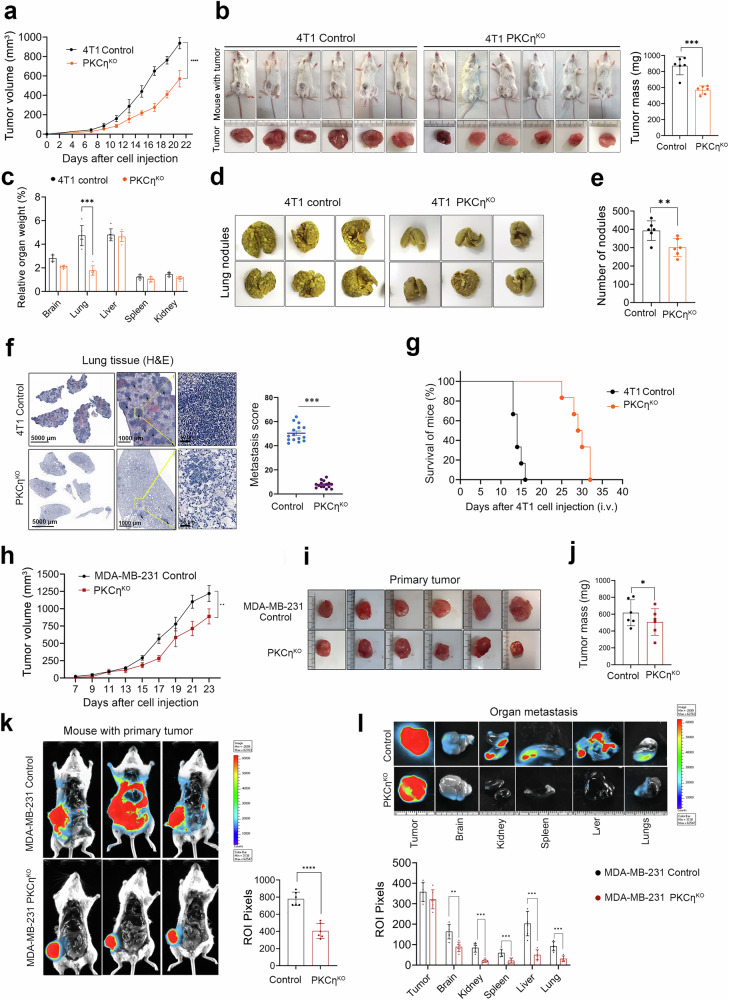
Orthotopic xenografts of 4T1 and MDA-MB-231 cells with PKCη^KO^ resulted in reduced metastasis. **a** Comparison of primary tumor growth between the control and 4T1 PKCη^KO^ cell xenograft groups, as evaluated by measuring the tumor volume on alternate days. **b** Images of primary mammary tumors (4T1 control and PKCη^KO^) were collected at the end of the experiment to determine the size and volume of the primary tumors. **c** Graphical representation of the relative organ weights of 4T1 control and PKCη^KO^ cells xenografted into NSG mice. The percentage relative lung weight increased significantly owing to the abundance of metastatic nodules in the 4T1 control group. **d** Images of the lungs were collected at the end of the experiment, which revealed abundant lung metastatic macronodules in the control 4T1 mice. **e** Quantitative analysis of metastatic lung nodules in the 4T1 control and PKCη^KO^ groups. **f** H&E staining of lung tissue sections revealed that, compared with control mice, mice injected with 4T1 PKCη^KO^ cells had significantly lower lung metastasis scores. The scale bar of the enlarged portion indicates 50 μm. **g** Mouse survival was increased by the ablation of PKCη in 4T1 cells. Female NSG mice were injected with 4T1 cells via the tail vein, and the survival (in days) of each mouse was recorded. Statistical significance was determined via survival curve comparison (log-rank (Mantel‒Cox) test, *p* = 0.0005). **h** MDA-MB-231 cell-xenografted mammary tumor growth was assessed by measuring the tumor volume on alternate days. **i** Image showing the primary tumor sizes of the control and PKCη^KO^ groups of MDA-MB-231 cells. **j** Graphical representation of the average tumor mass of the MDA-MB-231 control and PKCη^KO^ mice (six animals/group). **k** Representative bioluminescence images depicting the primary tumor size and metastasis of MDA-MB-231-PKCη^KO^-Luc xenografts in NSG mice. The bar diagram shows the mean bioluminescence intensity in the regions of interest (ROIs) in pixels. **l** Bioluminescence imaging of metastasis in various organs in control versus MDA-MB-231-PKCη^KO^-L uc-xenografted NSG mice, including a bar diagram quantifying the mean bioluminescence intensity across multiple organs at ROIs in pixels. The data are presented as the means ± SEMs (*n* = 6). Statistical significance was determined via two-way ANOVA, where *P < 0.05, **P < 0.01, ***P < 0.001, and ****P < 0.0001

To confirm the effect of PKCη^KO^ on TNBC metastasis in mice, we orthotopically injected 4T1 PKCη^KO^ or control cells and monitored primary tumor growth and metastasis in different organs. Compared with those derived from control cells, primary xenograft tumors derived from 4T1 PKCη^KO^ cells were diminished in size and mass (Fig. 3a, b). A comparative analysis of organ weights revealed a significant increase in the lung weight of mice inoculated with 4T1 control cells compared with those inoculated with PKCη^KO^ cells (due to abundant lung nodules) (Fig. 3c–e). Notably, histopathological analysis of the lung tissue revealed significant differences in both the frequency and size of macro- and micronodules (Fig. 3f).

To clarify whether the reduced metastasis observed in the mice injected with 4T1 PKCη^KO^ cells was independent of primary tumor growth delay, we euthanized the mice when the primary tumor volumes in the control and 4T1 PKCη^KO^ groups were comparable (Supplementary Fig. 13a, b). Despite similar tumor sizes, compared with control mice, 4T1 PKCη^KO^-injected mice presented a significantly reduced lung metastatic burden (Supplementary Fig. 13c–e). These findings establish that the reduction in metastasis reflects a direct prometastatic role of PKCη in TNBC.

To further confirm that PKCη is involved in TNBC metastasis, mice were injected with either 4T1 control cells or PKCη^KO^ cells through the tail vein. The control mice exhibited significant weight loss and died within 14.17 ± 1.1 days due to severe metastatic burden, whereas the mice injected with PKCη^KO^ cells presented prolonged survival of approximately 30 days (29.3 ± 2.4) (Fig. 3g and Supplementary Fig. 14a, b). Compared with PKCη^KO^ mice, control mice presented abundant lung metastatic nodules with fewer metastatic foci and a larger appearance (Supplementary Fig. 14c–e), suggesting a reduced initial colonization capacity but potential compensatory growth of lesions due to an extended lifespan.

For additional validation of metastasis, we used MDA-MB-231 cells tagged with luciferase (Luc), enabling the tracking of these cells in mice. MDA-MB-231 and PKCη^KO^ cells were orthotopically transplanted into the mammary fat pads of female mice. The development of primary xenograft tumors in these mice was closely monitored, and both the tumor volume and body weight were measured on alternate days. The data revealed a delay in the growth of primary tumors in mice inoculated with MDA-MB-231 PKCη^KO^ cells compared with those inoculated with control cells (Fig. 3h–j). No significant differences were observed in the relative weights of the organs examined (Supplementary Fig. 15a). Metastatic capability was evaluated via bioluminescence imaging across various organs in both MDA-MB-231 control and PKCη^KO^ mice. This analysis revealed greater bioluminescence signals in mice injected with control cells than in those injected with PKCη^KO^ cells (Fig. 3k). Importantly, mice injected with MDA-MB-231 control cells demonstrated widespread metastasis to critical organs, including the liver, lungs, spleen, kidneys, and brain (Fig. 3l), which was markedly reduced in mice injected with MDA-MB-231 PKCη^KO^ cells. Organs such as the lungs, liver, and brain were harvested, and subsequent H&E staining of tissue sections confirmed the presence of metastasis in the control tumors (Supplementary Fig. 15b–d). This effect was accompanied by a decrease in the proliferation marker Ki67 in PKCη^KO^ primary tumors compared with control tumors for both 4T1 and MDA-MB-231 cells (Supplementary Fig. 16a, b). Further support for the role of PKCη in promoting metastasis was obtained from in vivo rescue experiments in MDA-MB-231 cells, in which PKCη was re-expressed in PKCη-knockout cells, which revealed that the restored primary tumor growth and distant organ metastasis were comparable to the control levels (Supplementary Fig. 8e–j). Taken together, these results suggest that PKCη promotes tumor progression and metastasis in TNBC.

### PKCη promotes YAP activation and stability in TNBC

**Fig. 4:**
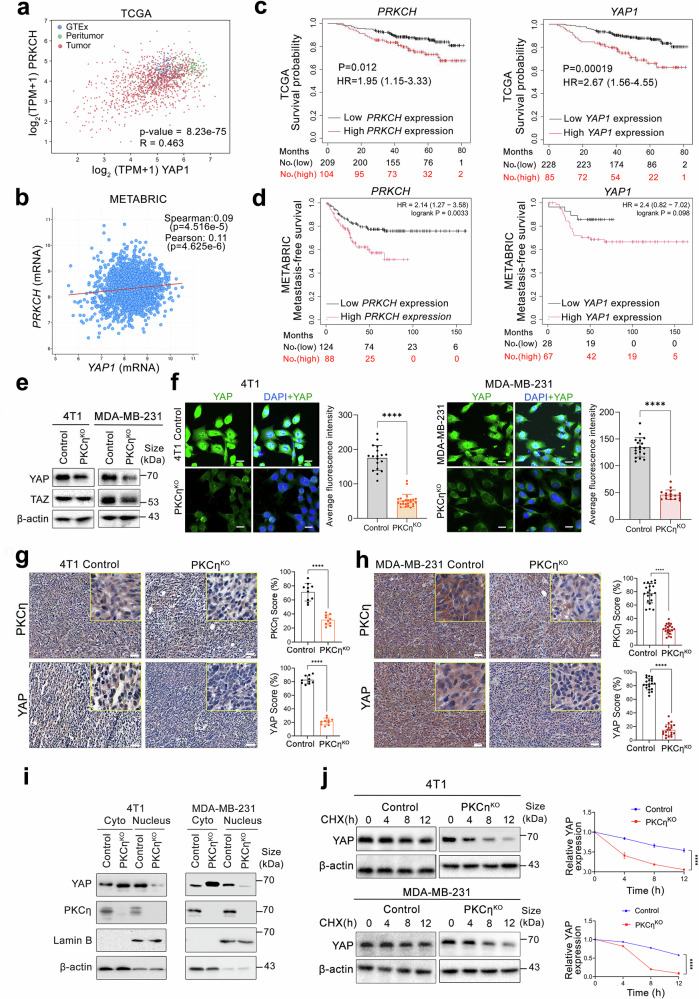
PKCη stabilizes the YAP protein in TNBC cells. **a**, **b** Scatter plots show a significant positive correlation between *PRKCH* and *YAP1* mRNA expression in patients with BC. TCGA dataset correlation analysis was performed via GEPIA3 with the TCGA-BRCA datasets (TCGA-Tumor, TCGA-Peritumor, and Genotype-Tissue Expression (GTEx)-normal reference). Correlation analysis was performed with the METABRIC datasets via cBioPortal. **c** Kaplan–Meier survival curves demonstrating that elevated *PRKCH* and *YAP* expression is associated with poor overall prognosis in BC patients. **d** Kaplan–Meier survival curves showing that elevated *PRKCH* and *YAP* expression correlated with decreased distant metastasis-free survival (DMFS) in TNBC patients in the METABRIC dataset. **e** Western blot analysis revealed reduced YAP expression in PKCη^KO^ 4T1 and MDA-MB-231 cells. **f** Immunofluorescence images of YAP nuclear and cytoplasmic expression in 4T1 and MDA-MB-231 control and PKCη^KO^ cells. YAP was stained green, and the cell nuclei were counterstained with DAPI (blue). The results of the quantitative analysis of these images are presented as the average fluorescence intensity/cell. The scale bar indicates 20 μm. **g** Representative images of IHC staining for PKCη and YAP in the primary tumors of 4T1 cells. Tumor tissue sections obtained from 4T1 cells xenografted with PKCη^KO^ breast tumors were immunostained with PKCη-specific and YAP-specific antibodies. The scale bar indicates 50 μm. **h** IHC staining highlighted the expression of PKCη and YAP in primary tumor tissues from MDA-MB-231 cells with PKCη^KO^ cells xenografted into female NSG mice, including quantification of the average protein expression of PKCη and YAP in both control and PKCη^KO^ primary tumor tissues. The scale bar indicates 50 μm. **i** Cytoplasmic and nuclear protein expression of PKCη and YAP in the nuclear and cytoplasmic fractions of 4T1 and MDA-MB-231 cells. The nuclear and cytoplasmic fractions were verified using the cellular markers lamin B and β-actin, respectively. **j** Time-course treatment of control and PKCη^KO^ cells derived from both the 4T1 and MDA-MB-231 cell lines with cycloheximide (10 μg/ml) revealed that the YAP protein level was stabilized by PKCη. The YAP protein concentration was normalized to that of β-actin. All quantification data are presented as the mean ± SEM. Statistical significance was determined via two-way ANOVA, where *P < 0.05, **P < 0.01, ***P < 0.001, and ****P < 0.0001

As a downstream effector of GPCR signaling, PKC has been suggested to bridge GPCR signaling pathways by regulating the Hippo–YAP pathway. GPCRs coupled with Gq/11 (*GNAQ/GNA11*), G12/13 (*GNA12* and *GNA13*), and Gi (*GNAI1*) have been shown to stimulate YAP/TAZ activation and nuclear translocation.^14^ We analyzed TCGA-BRCA patient samples and found that the RNA expression levels of G-coupled proteins, such as GNAQ/GNAI1, GNA12/GNA13, and GNAI1/GNAI3, were positively correlated with increased expression levels of *PRKCH* and *YAP1* (Supplementary Fig. 17a, b). Given the established roles of YAP and the Hippo pathway in the EMT and metastasis of TNBC,^40^ we tested the hypothesis that PKCη may regulate these pathways. We analyzed BC clinical samples from both the TCGA and METABRIC datasets and found that the *PRKCH* (PKCη) and *YAP1* mRNAs were positively correlated (Fig. 4a, b) and that elevated levels of either *YAP1* or *PRKCH* were associated with poor prognosis in BC patients (Fig. 4c). K‒M survival analysis of the METABRIC dataset revealed that TNBC patients with elevated *PRKCH* and *YAP1* expression levels had reduced distant metastasis-free survival (DMFS), indicating a poor prognostic effect of *PRKCH* and *YAP1* (Fig. 4d). Moreover, BC patients with high levels of both *YAP1* and *PRKCH* presented poorer prognostic outcomes than those with high YAP but low *PRKCH* levels did (Supplementary Fig. 18).

This positive correlation between YAP and PKCη was further validated in our cells, as the depletion of PKCη led to a decrease in YAP expression in both the 4T1 and MDA-MB-231 cell lines (Fig. 4e). TAZ expression was decreased in MDA-MB-231 PKCη^KO^ cells, which exhibited only cytoplasmic expression (Fig. 4e and Supplementary Fig. 19). Immunofluorescence analysis also revealed a significant reduction in YAP levels in the nucleus of 4T1 PKCη^KO^ cells, with residual YAP in the cytoplasm. MDA-MB-231 PKCη^KO^ cells also presented reduced YAP expression in the nucleus (Fig. 4f). Immunohistochemistry of tumor tissues revealed that YAP and PKCη expression was increased in 4T1 and MDA-MB-231 xenograft primary tumor tissues but decreased in PKCη^KO^ xenograft tumor tissues (Fig. 4g, h). Immunohistochemistry of the metastatic lung tissue of 4T1 xenografts revealed the presence of PKCη, with reduced expression in PKCη^KO^ xenografts (Supplementary Fig. 19b). Compared with that in control tissues, YAP expression was also significantly lower in the metastatic lung tissues of PKCη^KO^ xenografts (Supplementary Fig. 19c). Similarly, reduced YAP expression in PKCη^KO^ xenograft tumors was confirmed by western blotting (Supplementary Fig. 20a, b).

To validate the subcellular distribution of PKCη and YAP in TNBC, PKCη expression was tested in the nuclear and cytoplasmic fractions of both control and PKCηKO 4T1 and MDA-MB-231 cells. Nuclear YAP levels were significantly lower in PKCη^KO^ cells than in control cells across both cell lines, whereas cytoplasmic expression was increased (Fig. 4i). To further explore the role of PKCη in regulating YAP expression, we examined the time course of YAP expression in the presence of cycloheximide (CHX), a known inhibitor of protein synthesis. We showed that YAP expression was significantly reduced over time in PKCη^KO^ cells (Fig. 4j), highlighting the critical role of PKCη in maintaining YAP stability.

### PKCη phosphorylates YAP at Ser128

**Fig. 5:**
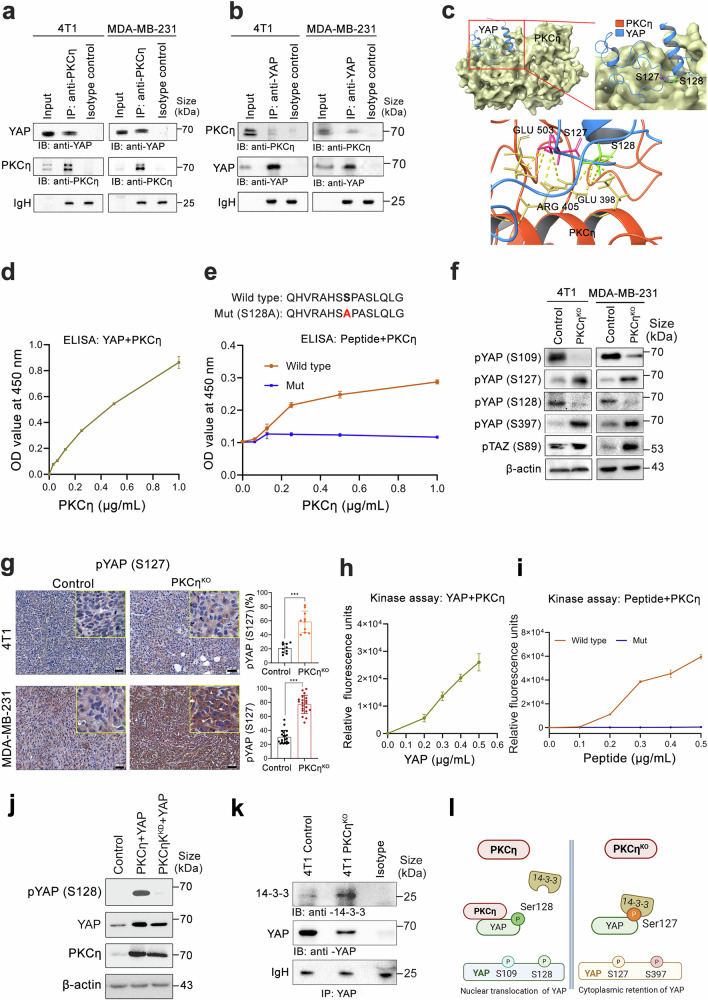
PKCη promotes YAP phosphorylation at Ser128 and attenuates the interaction of YAP at Ser127 with the 14-3-3 proteins. **a**, **b** Coimmunoprecipitation assays using 4T1 and MDA-MB-231 cell lysates demonstrated the interaction between PKCη and YAP (a mouse PKCη antibody was used for YAP pulldown, and a rabbit YAP antibody was used for PKCη pulldown). **c** Surface representation of the YAP1-PKCη docked pose. A structural model depicting the docked pose of YAP1-PKCη reveals the potential binding probability between the catalytic domain of PKCη and the TEAD-binding domain of YAP, highlighting the Ser128 and Ser127 phosphorylation sites. **d** ELISA depicting the binding affinity of YAP for PKCη (the YAP concentration was set at 1 µg/ml, and the PKCη concentration was tested between 0.1 and 1 µg/ml). **e** ELISAs were performed to determine the binding affinities of PKCη with YAP-derived wild-type and mutated peptides (at S128) (peptide concentrations were maintained at 1 µg/mL in the presence of varying PKCη levels (0.1 to 1 µg/mL)). **f** Immunoblotting depicting YAP phosphorylation at Ser109, Ser127, Ser128, Ser397 and TAZ Ser89 in 4T1 and MDA-MB-231 control cells and in cells devoid of PKCη. **g** Immunohistochemical (IHC) analysis of phosphorylated YAP (Ser127) in primary tumor tissues from xenografts of 4T1 and MDA-MB-231 cells (control and PKCη^KO^). Quantification revealed that pYAP (Ser127) expression was increased in PKCη^KO^ tumors compared with control tumors. The scale bar indicates 50 μm. **h** Kinase activity assay of PKCη phosphorylation on recombinant YAP. Phosphorylation is represented in relative fluorescence units (RFUs). **i** Kinase activity assay of PKCη phosphorylation of YAP-derived peptides: wild-type and Ser128-mutated peptides. **j** HEK293FT cells were transfected with YAP alone (control), YAP + wild-type PKCη (active), or YAP + kinase-dead PKCη (PKCη^KD^). Western blot analysis revealed that YAP S128 phosphorylation occurred only in cells overexpressing YAP together with active PKCη but not with PKCη^KD^, demonstrating that PKCη kinase activity is essential for this phosphorylation event. **k** Coimmunoprecipitation assays using 4T1 cell lysates demonstrated an interaction between YAP and 14-3-3 (a mouse YAP antibody was used for 14-3-3 pull-down). **l** Schematic representation of YAP activation by PKCη. We propose that PKCη expression leads to YAP activation (pYAP Ser128) and nuclear retention, disrupting the phosphorylation-dependent interaction between YAP (pYAP Ser127) and 14-3-3. Statistical significance was determined via two-way ANOVA, where *P < 0.05, **P < 0.01, ***P < 0.001, and ****P < 0.0001

To assess the potential interaction between PKCη and YAP, coimmunoprecipitation experiments were performed, revealing a direct physical interaction between the endogenous forms of PKCη and YAP (Fig. 5a, b). Protein‒protein docking analysis further supported the binding between YAP and PKCη (Fig. 5c). PKCη and YAP1 (TEAD-binding domain) were docked (Supplementary Fig. 21), and the HDOCK server predicted the docked pose at a confidence score of 0.86 (range: 0–1) and a docking score of −242.46 (Supplementary Fig. 21b). Protein‒protein docking predicted that YAP1 predominantly interacts with the kinase domain of PKCη, which is composed primarily of α-helices and β-sheets. The interactions between YAP1 and PKCη are shown in Supplementary Fig. 21b. The Ser127 and Ser128 residues of YAP1 formed four hydrogen bonds with the amino acid residues Glu398, Arg405, and Glu503 of PKCη. Both Ser127 and Ser128 have been reported to be crucial for the cytoplasmic retention and activation of YAP1, respectively (Fig. 5c).^41^ On the basis of these findings, we expected that the interaction between YAP1 and PKCη might be responsible for the phosphorylation of Ser128 and the physical hindrance posed by the presence of PKCη to prevent Ser127 phosphorylation by LATS. The protection of Ser127 phosphorylation from LATS was further indicated by the presence of the α-helical structure of YAP (residues 61–73) and the coiled structures (residues 116–123), which may act as a barrier to prevent any further access to the Ser127 site (Fig. 5c).

Further validation of the PKCη-YAP interaction was performed via ELISA (Fig. 5d). To identify the precise phosphorylation site of YAP, we synthesized wild-type and mutated peptide fragments of YAP with an alanine substitution at Ser128. The results showed that only the wild-type peptide could bind to PKCη (Fig. 5e), suggesting that Ser128 is critical for the binding of PKCη to YAP (also indicated by molecular docking). Compared with that in control cells, the phosphorylation of YAP at Ser109 and Ser128 was markedly reduced in PKCη^KO^ cells in both cell lines, whereas the phosphorylation of YAP at Ser127 and Ser397 and TAZ at Ser89 was significantly increased (Fig. 5f). Immunohistochemical analysis of pYAP (Ser127) in xenograft tumor tissues from 4T1 and MDA-MB-231 cells and their PKCη^KO^ cells further demonstrated increased YAP phosphorylation at Ser127 in the cytoplasm of PKCη^KO^ cells, which was consistent with cytoplasmic retention of YAP (Fig. 5h). Notably, the reintroduction of PKCη into MDA-MB-231 PKCη^KO^ cells restored YAP expression and its Ser128 phosphorylation (Supplementary Fig. 8d). Finally, acute siRNA-mediated PKCη suppression in 4T1 and MDA-MB-231 cells, excluding potential long-term cell adaptation caused by PKCη knockout, induced YAP phosphorylation at Ser127 and Ser397 and its subsequent degradation (Supplementary Fig. 9d).

To confirm the role of PKCη in YAP phosphorylation and activation, PKCη was overexpressed in both TNBC cell lines. This resulted in increased YAP expression and phosphorylation at Ser128 (Supplementary Fig. 22). To further examine the direct phosphorylation of YAP by PKCη at Ser128, we conducted in vitro kinase assays using active recombinant PKCη and YAP. A concentration-dependent increase in PKCη kinase activity was observed (Fig. 5h). Immunoblot analysis of the kinase assay end products validated the phosphorylation of YAP at Ser128 in the presence of PKCη (Supplementary Fig. 23). To further validate the specific site of YAP phosphorylation, we used wild-type YAP and a mutated peptide with alanine substitutions at Ser128. Only the wild-type YAP peptide was phosphorylated by PKCη, and the mutated variant showed negligible phosphorylation (Fig. 5i). To further demonstrate that the kinase activity of PKCη is essential for YAP Ser128 phosphorylation, wild-type (active kinase) and PKCη-kinase-dead (PKCη^KD^) constructs were overexpressed in HEK293FT cells. Western blot analysis using a phospho-specific YAP Ser128 antibody revealed that YAP Ser128 phosphorylation was detected only in cells coexpressing YAP with wild-type PKCη but not in control cells (expressing YAP alone) or in cells coexpressing YAP with PKCη^KD^ (Fig. 5j). These findings demonstrated that Ser128 is a critical site for PKCη-mediated phosphorylation of YAP, facilitating its nuclear translocation. Furthermore, to assess the specificity of PKC isoforms in mediating YAP phosphorylation, different novel PKC isoforms (PKCδ, PKCε, and PKCη) were co-overexpressed in HEK293FT cells with YAP. YAP128 phosphorylation occurred only in the presence of PKCη but not in the presence of PKCε or PKCδ (Supplementary Fig. 24). This isoform-specific phosphorylation of YAP highlights the specific regulatory role of PKCη in YAP-activating signaling and its regulation of the Hippo pathway.

Previous studies have revealed that phosphorylation of YAP at Ser127 can create a docking site for 14-3-3 proteins, leading to inactivation of YAP and its retention in the cytoplasm.^41^ In the absence of PKCη, YAP is phosphorylated at Ser127, leading to its association with 14-3-3 proteins and subsequent cytoplasmic retention (Fig. 5f, g, k). Coimmunoprecipitation experiments further validated the association of phosphorylated YAP (Ser127) with 14-3-3ζ in 4T1 PKCη^KO^ cells (Fig. 5k). Overall, our results suggest that PKCη promotes YAP phosphorylation at Ser128, leading to YAP activation. Conversely, PKCη ablation facilitates YAP phosphorylation at Ser127, which promotes 14-3-3 binding and subsequent cytoplasmic retention (Fig. 5l).

### PKCη is a negative regulator of AKT that phosphorylates the upstream kinase cascade of the Hippo pathway

**Fig. 6:**
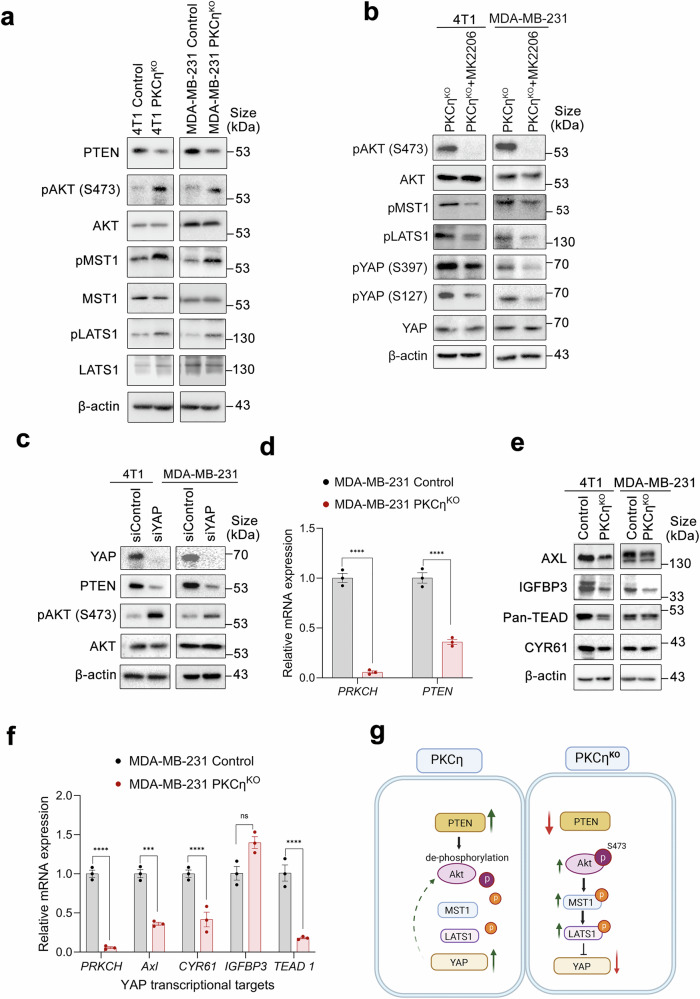
AKT phosphorylation of the upstream kinase cascade of the Hippo pathway is negatively regulated by PKCη. **a** Western blot analysis showing the activation of members of the upstream kinase cascade of the Hippo pathway, which is regulated by AKT phosphorylation, in PKCη^KO^ TNBC cells. PKCη promotes YAP and PTEN expression, leading to dephosphorylation and inactivation of AKT. **b** An AKT inhibitor (MK-2206) effectively inhibits the upstream Hippo pathway phospho-cascade regulated by AKT in PKCη^KO^ TNBC cells. **c** YAP-specific siRNA downregulates PTEN expression in TNBC cells in conjunction with AKT activation. **d** Relative mRNA expression of *PTEN* and *PRKCH* in control and MDA-MB-231 PKCη^KO^ cells, as determined by RT‒qPCR. Knockout of PKCη in MDA-MB-231 cells resulted in a marked reduction in PTEN transcript levels compared with those in control cells. **e** Impact of PKCη expression on YAP transcriptional targets in TNBC cells. **f** Relative mRNA expression of canonical YAP target genes (*AXL, CYR61, IGFBP3*, and *TEAD*) was measured via RT‒qPCR in MDA-MB-231 PKCη^KO^ and control cells. PKCη knockout results in a significant reduction in the expression of these downstream effectors. **g** Schematic illustration of how YAP activity regulates PTEN expression and modulates the AKT pathway through this feedback mechanism (in the presence or absence of PKCη). Statistical significance was determined via two-way ANOVA, where *P < 0.05, **P < 0.01, ***P < 0.001, and ****P < 0.0001

YAP is a core regulator of the Hippo pathway and is phosphorylated by the MST1/LATS1 cascade downstream of GPCRs.^14^ Immunoblot analyses of 4T1 and MDA-MB-231 cells revealed that ablation of PKCη was correlated with increased AKT phosphorylation (S473), pMST1 (Thr183), and pLATS1 (Thr1079) and decreased PTEN expression (Fig. 6a). To explore the interplay between AKT phosphorylation and PKCη, cells were treated with or without the AKT inhibitor MK-2206. The results revealed marked dephosphorylation of AKT in PKC^KO^ cells, along with a decrease in the phosphorylation of the kinases MST1 and LATS1 and a decrease in YAP phosphorylation at Ser127 and Ser397 (Fig. 6b). Inhibition of AKT phosphorylation by PTEN suppresses the phosphorylation cascade of upstream Hippo kinases, thereby promoting YAP activation.^42^ To explore whether YAP is involved in PTEN expression and subsequent AKT inhibition, we silenced YAP1 in 4T1 and MDA-MB-231 cells with siRNA, which resulted in a significant decrease in the protein expression levels of YAP and PTEN in both cell lines, accompanied by increased AKT phosphorylation (Fig. 6c). To determine whether PTEN expression is regulated by PKCη at the transcriptional level, the relative mRNA levels of PTEN in control and MDA-MB-231 PKCη^KO^ cells were determined via RT‒qPCR. Our results revealed a significant reduction in PTEN transcript levels compared with those in control cells (Fig. 6d). Reduced YAP expression in PKCη^KO^ cells (Fig. 6c) was accompanied by decreased expression of YAP/TAZ transcriptional targets, at both the protein and mRNA levels, including AXL, IGFBP3, TEAD, and CYR61, in both cell lines (Fig. 6e, f). Overall, we demonstrated that PKCη plays a negative role in AKT activation by increasing PTEN expression, which subsequently leads to the suppression of the Hippo pathway and YAP activation (Fig. 6g).

### A uORF-encoded micropeptide causes PKCη degradation and downregulation of YAP activity in vitro and in vivo

**Fig. 7:**
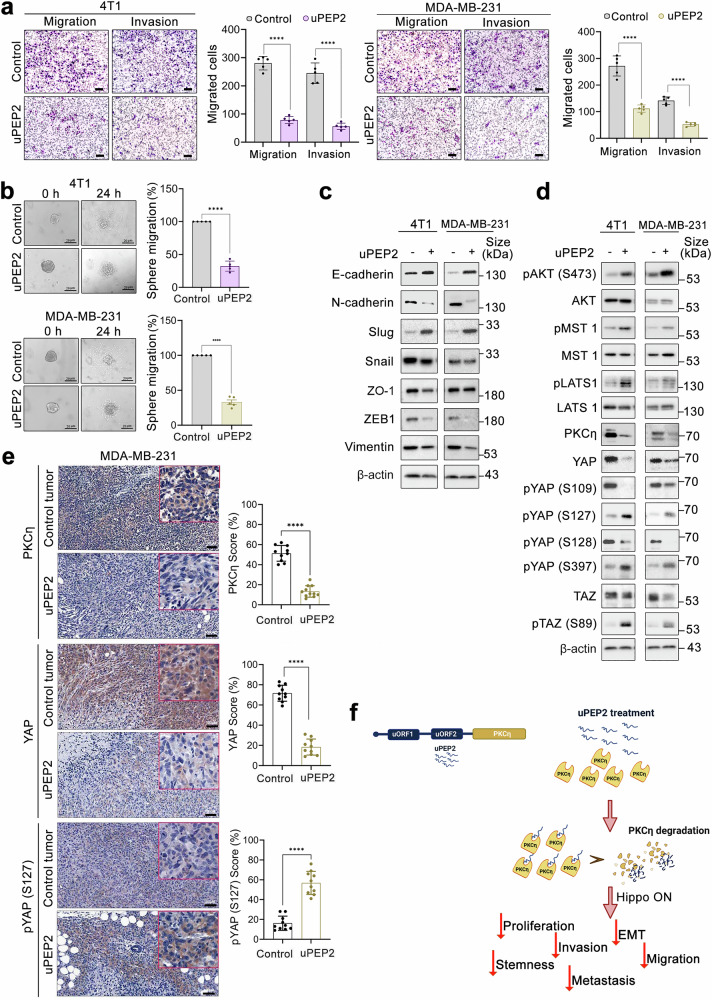
The uORF2-encoded peptide (uPEP2) upstream of PKCη degrades PKCη and downregulates the YAP-Hippo pathway both in vitro and in vivo. **a** Compared with control cells, 4T1 and MDA-MB-231 cells treated with uPEP2 presented decreased migration and invasion capabilities. Migration was evaluated via a Boyden chamber assay. Invasion was assessed using Matrigel-coated Boyden chamber chambers. The scale bar indicates 50 μm. **b** uPEP2 inhibits sphere cell invasion through Matrigel. The area of the invaded zone was measured after 24 h, and the results are shown (*n* = 5). The scale bar indicates 20 μm. **c** Effects of uPEP2 treatment on EMT markers in 4T1 and MDA-MB-231 cells. **d** uPEP2 treatment of 4T1 and MDA-MB-231 cells downregulated PKCη and YAP expression and affected members of the Hippo pathway and their phosphorylation. **e** Immunohistochemical analysis of PKCη, YAP, and pYAP (S127) expression in uPEP2-treated MDA-MB-231 xenograft primary tumors. Representative images and quantitative analyses are presented. Scale bar, 100 μm. **f** Schematic of the effect of uPEP2 on PKCη and its downstream functions. uORF2-encoded uPEP2 (blue) inhibits the catalytic activity of PKCη (yellow) and causes PKCη degradation, leading to reduced cell proliferation, EMT, stemness, migration, invasion, and metastasis. The data are expressed as the means ± SEMs (*n* = 3). Statistical significance was determined via two-way ANOVA, where *P < 0.05, **P < 0.01, ***P < 0.001, and ****P < 0.0001

Our recent findings revealed that a micropeptide (uPEP2), encoded by a uORF in the 5’UTR of PKCη mRNA, inhibits the kinase activity of PKCη and leads to a reduction in tumor progression and metastasis.^21^ In the present study, we investigated the effects of uPEP2 on the migration and invasion abilities of the 4T1 and MDA-MB-231 cell lines via transwell migration and invasion assays. uPEP2 significantly decreased the migration and invasion of both cell lines (Fig. 7a). Furthermore, uPEP2 treatment affected stem cell migration, as indicated by the results of the sphere cell invasion assay in Matrigel (Fig. 7b). The effect of uPEP2 treatment on EMT was also studied by examining markers, including E-cadherin, N-cadherin, Slug, Snail, ZO-1, ZEB1, and vimentin demonstrating the capacity of uPEP2 to reduce EMT characteristics in TNBC cells (Fig. 7c). The effects of uPEP2 treatment on PKCη expression and the subsequent effects on YAP were also determined. Treatment of 4T1 and MDA-MB-231 cells with uPEP2 resulted in a reduction in PKCη and YAP/TAZ expression and a decrease in YAP phosphorylation at Ser128, accompanied by increased phosphorylation at specific YAP Ser127 and Ser397 sites (Fig. 7d). This pattern strongly resembled the phosphorylation profile observed in PKCη^KO^ cells (see Fig. 5). Notably, uPEP2 treatment led to increased phosphorylation of AKT, MST1, and LATS1, which are the key upstream regulators of the Hippo pathway (Fig. 7d). Immunohistochemical analysis of MDA-MB-231 tumors treated with uPEP2 (as described previously^21^) revealed a significant reduction in PKCη and YAP expression, with elevated YAP phosphorylation at Ser127 (Fig. 7e), which was associated with reduced proliferative micrometastasis in the liver and lungs (Supplementary Fig. 25a, b). Taken together, our findings suggest that uPEP2 has therapeutic potential for suppressing tumor growth and metastasis by modulating the PKCη-YAP-Hippo signaling axis (Fig. 7f).

## Discussion

TNBC is a highly aggressive and metastatic form of cancer that has fewer treatment options than other types of BC.^43,44^ Thus, elucidating the molecular mechanisms mediating TNBC metastasis could reveal new targets for intervention. In this study, using in vitro and in vivo models, we demonstrated the role of PKCη in promoting TNBC cell migration and invasion and established that its expression is required to maintain EMT, stemness, and metastasis. We further delineated the molecular mechanisms mediated by PKCη in TNBC metastasis by regulating the Hippo pathway via the direct activation of YAP.

Analysis of BC patient data revealed elevated levels of PKCη expression in the “claudin-low” subtype, which accounts for 75% of TNBC patients.^28,29,45^ An enriched stem cell-like population and increased EMT activity characterize the “claudin-low” subtype. Our analysis demonstrated that, in patients overexpressing PKCη, a significant EMT activation signature was observed across several BC cohorts. Moreover, analysis of patient-matched primary and metastatic breast tumor samples revealed significant upregulation of PRKCH during metastatic progression, supporting its role as a driver of tumor aggressiveness beyond subtype-specific expression. Importantly, TNBC tissue microarray analysis further revealed that PKCη predominantly displayed perinuclear localization in TNBC patient samples and cell lines, a characteristic of an active PKCη kinase.^25,36,37^ This perinuclear distribution significantly correlated with a higher tumor grade and stage and was more pronounced in advanced tumors (Fig. 1d). These findings corroborated our transcriptomic data, establishing a strong association between PKCη expression and TNBC malignancy.

During EMT, epithelial cells lose their ability to adhere to cells and gain migratory and invasive properties to become mesenchymal stem cell-like populations, leading to the initiation of BC metastasis.^46,47^ This is accompanied by altered gene expression profiles and the acquisition of specific markers characteristic of the epithelial and mesenchymal state.^7^ In our study, we used the TNBC cell lines MDA-MB-231 and 4T1, which belong to the “claudin-low” subtype, and ablated PKCη expression to investigate its role in EMT.^28^ PKCη suppressed the epithelial markers E-cadherin and EpCAM and upregulated vimentin, N-cadherin and ZEB1, resulting in a highly invasive phenotype (Fig. 1). Furthermore, overexpressing PKCη in nonaggressive luminal MCF7 cells was sufficient to drive EMT marker expression and enhance invasive capacity (Supplementary Fig. 10a). Recent studies have indicated that EMT occurs in a gradual manner, exhibiting intermediate characteristics of epithelial and mesenchymal markers, which are referred to as partial EMT states.^48–50^ PKCη promotes ZEB1 expression but reduces Slug expression but has no effect on Snail, suggesting partial EMT. According to recent reports, partial EMT in tumor cells contributes to high rates of metastasis. Cells undergoing partial EMT exhibit enhanced collective cell invasion and are required for lung metastasis in BC.^50,51^ Several PKC isoforms have been implicated in promoting EMT through diverse, context-dependent mechanisms. For example, PKCα enhances ZEB1 expression to drive EMT and invasiveness in breast cancer.^16^ Inactivation of PKCδ inhibits cancer stem cell proliferation and survival,^18^ whereas PKCλ is required for the function of ALDH^+^ breast cancer stem cells. PKCθ promotes cancer stem cell formation by translocating to the nucleus and forming complexes with ZEB1.^19,20^ In our experiments, PKCη did not affect Snail expression in TNBC cells; however, in nontumorigenic MCF10A cells, TGFβ2-induced Snail expression was PKCε dependent.^17^ The overexpression of PKCη in luminal nontumorigenic MCF7 cells increased Snail expression without affecting ZEB1.

An important attribute exacerbating TNBC is increased CSCs, which are associated with cancer recurrence and metastasis.^52–54^ An increased CD44^+^/CD24^-^ ratio and ALDH^+^ expression are positively correlated with increased self-renewal, proliferation, tumor growth, and metastasis.^55^ The presence of these markers in BC promotes invasiveness and serves as an indicator of poor prognosis, which is associated with drug resistance and cancer relapse. Interestingly, ablation of PKCη in 4T1 and MDA-MB-231 cells significantly decreased the number of CD44^+^/CD24^-^ and ALDH^+^ cells. In addition, sphere formation and CSC invasion were reduced by PKCη depletion, along with a significant reduction in the expression of the CSC markers Sox2 and Nanog, suggesting a role for PKCη in stemness.

Different signaling pathways have been implicated in EMT stemness and metastasis, of which the Hippo pathway is the most studied.^8,56^ YAP/TAZ, two key downstream targets of the Hippo pathway, are frequently associated with human cancer metastasis. Analysis of BC patients with high metastatic potential and poor survival revealed a positive correlation between PKCη and YAP expression levels. Both PKCη and the Hippo–YAP pathway were shown to be activated by the extracellular signaling of GPCRs.^8,15,57^ Indeed, our data analysis of BC patient samples demonstrated that G proteins (Gq/11, G12/13, and Gi/o) were positively associated with elevated expression levels of PKCη.

**Fig. 8:**
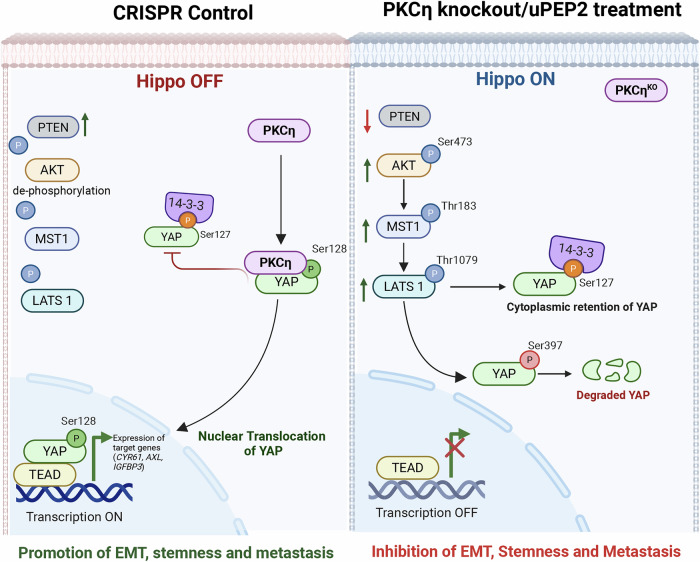
Schematic representation of the molecular mechanism of PKCη-YAP-mediated regulation of the Hippo pathway and metastasis in TNBC. CRISPR Control: In the normal state of TNBC, PKCη inhibits the Hippo pathway (Hippo-OFF), promoting S128 phosphorylation and stabilization, which leads to the nuclear import of YAP, where it regulates the transcription of several genes related to EMT, stemness, and metastasis. Additionally, YAP regulates PTEN transcription, thereby inhibiting AKT activation of the Hippo pathway through this feedback mechanism. The activation of the PKCη-YAP axis enhances migration and invasion, thereby increasing the metastatic potential and facilitating secondary spread to the lungs, liver, and brain. PKCη knockout/uPEP2 treatment: YAP and PTEN expression decreased in the absence of PKCη. Activation of the Hippo pathway (Hippo-ON) leads to increased phosphorylation and activation of AKT, MST1, and LATS1. Phosphorylation of YAP results in the recruitment of 14-3-3 proteins that mediate cytoplasmic retention or proteolytic degradation. Treatment with uPEP2 inhibits PKCη kinase activity and decreases protein expression. Thus, pharmacological treatment of TNBC with uPEP2 phenocopied the effects of PKCη^KO^ in BC cells, reducing their metastatic potential and reversing EMT and stemness *via* the PKCη–YAP Hippo pathway axis (created at https://BioRender.com)

The activation, degradation, and nuclear translocation of YAP and TAZ are critically dependent on their phosphorylation status.^58^ Phosphorylation of YAP at Ser127 and TAZ at Ser89 is associated with cytoplasmic retention and inactivation, whereas phosphorylation of YAP at Ser397 is associated with proteasomal degradation. YAP phosphorylation at Ser128 prevents 14-3-3 binding, thereby overriding the inhibitory effects of the canonical Hippo pathway via Ser127 phosphorylation, which leads to YAP activation and nuclear localization.^59^ In the nucleus, YAP binds to TEAD, initiating the transcription of genes that promote EMT and metastasis (Fig. 8).^60–62^ In this study, we demonstrated that PKCη directly phosphorylates Ser128 of YAP (Fig. 5), unlike other novel PKCs, highlighting the specificity of PKCη kinase activity. Phosphorylation of YAP at Ser128 was previously implicated to occur by Nemo-like kinase (NLK), which is overexpressed in approximately 20% of BCs, especially aggressive endocrine-resistant breast tumor subtypes.^59,63^ NLK exhibits context-dependent dual functionality in breast cancer (BC), acting as both a tumor suppressor and an oncogene.^63^ NLK was reported to phosphorylate YAP at Ser128 in HEK293T cells under osmotic stress conditions.^59^ Notably, our analysis of PKCη expression in the luminal B cohort revealed a negative correlation with NLK expression. Moreover, a negative correlation between PKCη and NLK was also found in the “claudin-low” population (Supplementary Fig. 26), suggesting that PKCη and NLK could act in a complementary manner in specific subtypes of BC. Hence, our study suggests that PKCη could be another kinase that binds to and phosphorylates YAP at Ser128 in TNBC.

The Hippo pathway mediates YAP Ser127 phosphorylation via the MST and LATS phosphorylation cascades, where AKT acts as an upstream regulator of this cascade. Depletion of PKCη activated the MST-LATS-YAP pathway through increased phosphorylation of AKT, leading to the retention of YAP in the cytoplasm and its degradation (Hippo ON pathway). Importantly, we showed that the upregulation of YAP by PKCη increased the expression of PTEN, which has been shown to be a negative regulator of AKT.^64^ Our studies suggest that PKCη regulates YAP via two mechanisms: (i) PKCη promotes YAP activation through direct phosphorylation, leading to its nuclear translocation (Fig. 5). (ii) PKCη regulates the expression of PTEN at the transcriptional level. This increase in PTEN expression resulted in AKT inactivation and suppression of the upstream phosphorylation cascade of the Hippo pathway, enabling sustained YAP activation (Fig. 6).^65^ We previously reported that PKCη acts as a negative regulator of AKT.^65,66^ In contrast to our observations, some studies reported that YAP downregulates PTEN expression.^67^ These findings are consistent with our findings in PKCη^KO^, where in the absence of PKCη, both YAP and PTEN expression are suppressed (Fig. 6). Thus, the presence of PKCη in a subset of aggressive tumors could be responsible for the subsequent activation of the PKCη-YAP signaling pathway, which is associated with an aggressive tumor phenotype and metastasis. Furthermore, both high YAP and PKCη expression predicted poor prognosis in patients with BC (Fig. 4c), highlighting the clinical relevance of PKCη in BC.

We recently characterized a PKCη degrader peptide encoded by an evolutionarily conserved uORF (uPEP2) upstream of the main coding sequence of PKCη.^21^ The endogenous expression of uPEP2 has been demonstrated in various cell lines, including MCF7, MOLT-4, and BeWo, where it acts both as a cis-repressor element that suppresses the translation of PKCη and as a kinase inhibitor, leading to destabilization and degradation of PKCη to maintain its low basal levels.^21,24^ uPEP2 also acts in trans, leading to the downregulation of other novel PKC isoforms. Under stress conditions, the translation of PKCη, a stress response kinase, is upregulated through leaky scanning dependent on eIF-2α phosphorylation by GCN2.^68^ We previously reported that uPEP2 treatment effectively reduced tumor progression and metastasis in the lungs and liver of MDA-MB-231 xenograft models.^21^ Here, we further showed that treatment with uPEP2 significantly reduced the EMT and stemness characteristics of MDA-MB-231 and 4T1 cells. Additionally, uPEP2 treatment attenuated YAP levels in the latter cells, accompanied by an increase in YAP phosphorylation at Ser127 or Ser397 and decreased phosphorylation of YAP at Ser128, suggesting its role in promoting cytoplasmic retention and degradation of YAP (Fig. 7). Hence, knockout of PKCη in TNBC cells and pharmacological treatment with uPEP2 resulted in similar signaling pathway alterations that collectively supported the direct role of PKCη in regulating the Hippo–YAP pathway axis. Our experiments demonstrated that PKCη is the only novel PKC capable of activating and stabilizing YAP via Ser128 phosphorylation, whereas PKCη suppressed Ser127 phosphorylation, leading to YAP activation. This specificity is important when considering the broader implications of Hippo–YAP signaling regulation by PKCη and the impact of uPEP2 as a therapeutic agent (Fig. 8). Gong et al. (2015) described opposing roles of novel and conventional PKCs in regulating the Hippo–YAP pathway, where novel PKCs activate the LATS kinase and conventional PKCs inhibit its activity.^15^ However, even in their study, PKCη behaves differently from other novel PKCs, as it decreases YAP phosphorylation at Ser127 in the absence of TPA and suppresses TPA-induced YAP Ser127 phosphorylation, in accordance with our data.^15^ Taken together, our results highlight the unique role of PKCη in YAP Ser128 phosphorylation and activation.

Our study provides insights into the role of PKCη in TNBC metastasis; however, several limitations should be considered. Our in vivo studies were conducted in immunodeficient mouse models, which may not fully capture the complex interactions between tumors and the immune system in patients. Here, we focused on the PKCη isoform, although we cannot deny the possibility that other PKCs contribute to BC progression via other mechanisms. We previously reported the synergistic action of uPEP2 with chemotherapy.^21^ Therefore, further research should evaluate the therapeutic benefit of combining PKCη inhibition with existing BC therapies, particularly in YAP-induced resistance cohorts. The development of allosteric inhibitors that disrupt the PKCη–YAP interaction suggests an alternative approach for modulating this pathway and therapeutic intervention. In this context, a rigorous parallel clinical assessment of PKCη and YAP as prognostic biomarkers in BC patients may enable more accurate risk management and the development of personalized treatment approaches. Future research should explore the clinical applicability of uPEP2 as a targeted therapeutic agent.

Overall, our study identified a novel role for PKCη as a negative regulator of the Hippo pathway and demonstrated its function in promoting EMT, stemness, and metastasis by activating YAP in TNBC. Our findings suggest that PKCη could represent a therapeutic target for this highly lethal and metastatic BC disease, for which uPEP2, the recently discovered uORF-encoded kinase inhibitory peptide (upstream of PKCη), could be utilized as a therapy.

## Materials and methods

### Cell culture and cell lines

4T1, MDA-MB-231, 4T1 scrambled, 4T1 PKCη^KO^, MDA-MB-231 scrambled, MDA-MB-231 PKCη^KO^, MDA-MB-231 PKCη Rescue, MDA-MB-231-Luc, MDA-MB-231-Luc PKCη^KO^, MCF7, MCF7 PKCη-OE, MDA-MB-231 PKCη-OE, 4T1 PKCη-OE and HEK293FT cells were maintained in complete Dulbecco’s modified Eagle’s medium (DMEM) (no. 01–055-1A, Biological Industries). The media were supplemented with penicillin (100 units/mL), streptomycin (0.1 mg/mL), L-glutamine (200 mM, Sartorius, 03-020 1 B), sodium pyruvate (Sartorius, 03-042 1 B), and 10% fetal bovine serum (Biowest, S1400-500) for the cell lines. All the cells were maintained at 37 °C in a humidified incubator with 5% CO_2_. Mycoplasma contamination was not detected via a Mycoplasma Detection Kit (HyLabs).

### Peptides

uPEP2 (Myr-MASRGALRRCLSPGLPRLLHLSRGLA) was synthesized by GL-Biochem Ltd. (Shanghai, China). The peptide contained a C-terminal amidation for stability and a myristoyl group at the N-terminus to enable cell penetration. The short peptides used for the kinase assay were as follows: wild-type peptide (wild-type: QHVRAHSSPASLQLG) and Ser128-mutated peptide (S128A) (Mut: QHVRAHS**A**PASLQLG).

### PKCη knockout cell line generation via CRISPR/Cas9 technology

In this study, we engineered a CRISPR-Cas9 lentiviral system on the basis of the protocol described by Zhang’s laboratory.^69,70^ The PKCη gRNA sequence targeting exon 1 of the murine *PRKCH* gene (for 4T1) and nonspecific scrambled sequences were designed via Benchling software and synthesized by Sigma, Israel, and the human *PRKCH* gene (for MDA-MB-231 and MDA-MB-231 Luc) and the corresponding nonspecific scrambled sequences were designed via Benchling software and synthesized by IDT-Syntezza, Israel (Supplementary Table 6). The LentiCRISPRv2 vector was digested with *BsmBI* (NEB) and dephosphorylated with sheep alkaline phosphatase for 30 min at 37 °C. The digested plasmid was purified via a GeneJet Gel Extraction Kit (Thermo Scientific Inc., USA). The gRNA and scrambled oligos were phosphorylated and annealed via T4 PNK (NEB) in T4 ligation buffer (Promega) with the following parameters: 37 °C for 30 min, 95 °C for 5 min, and a ramp down to 25 °C at 5 °C/min. Fifty nanograms of the digested plasmid and 1:100 diluted oligos were ligated via T4 ligase (NEB) for 30 min at RT. The ligation mixture was transformed into DH5α cells, which were spread onto ampicillin agar plates and incubated overnight at 37 °C. Positive clones were selected and cultured, and cloning was confirmed via colony PCR and sequencing. The plasmids were amplified via DH5α and purified via the MaxiPrep Plasmid DNA Kit (Invitrogen, USA). LentiCRISPRv2Puro carrying PKCη gRNA or scrambled constructs was packaged into lentiviral particles via a mixture of plasmids containing the lentiviral packaging genes VSVG and psPAX2. The mixture and the specific plasmid were transfected into subconfluent HEK293T cells via the PEI reagent. HEK293T cells were maintained in DMEM supplemented with 10% FCS and antibiotics and incubated overnight at 37 °C and 5% CO_2_. The medium was replaced 24 h after transfection, and 48 h later, the supernatants were collected, and the cell debris was removed via centrifugation and passed through a 0.45 µm filter. The viruses were concentrated via Amicon filter tubes (100 kDa MWCO) (Merck Millipore, USA). Fresh cultures of mouse 4T1, human MDA-MB-231, and MDA-MB-231 Luc cells were then treated with the corresponding viral suspension mixture containing 10 µg/ml polybrene. Positive clones were isolated via puromycin selection. *PRKCH* deletion in the established single-cell clones was confirmed by sequencing and western blotting.

### GSEA characterization of transcriptional signatures associated with *PRKCH* overexpression in tumors

To determine the transcriptional signatures associated with *PRKCH* overexpression, we partitioned BC tumors on the basis of their *PRKCH* levels. Specifically, we performed an analysis to infer differentially expressed genes (DEGs) between the PKCη-high tumors (top 80%) and PKCη-low tumors (bottom 20%) via the Limma R package (v3.60.2). Next, we performed gene set enrichment analysis (GSEA) via the fgsea package (v1.30.0) with an aggregated collection of gene sets from MSigDB v2024.1.^71^

### Orthotopic xenograft BC models in NSG mice generated from 4T1 and MDA-MB-231 cells with and without PKCη

The mice were housed in air-filtered laminar flow cabinets with a 12/12 h light‒dark cycle and provided with food and water ad libitum. The mice were housed and handled in accordance with the institutional guidelines of Ben-Gurion University of Negev. The animal experimental protocol was approved by the Institutional Animal Ethics Committee. All the animal experiments were conducted with 6–8-wk-old female NSG mice (The Jackson Laboratory, NOD.Cg-PrkdcscidIl2rgtm1Wjl/SzJ). 4T1 (1 × 10^6^), MDA-MB-231-Luc (4 × 10^6^), and their respective PKCη^KO^ cells were suspended in PBS (100 μL) and injected subcutaneously into the mammary fat pads of NSG female mice.^21^ The tumor volume was measured on alternate days via a digital Vernier caliper. The tumor volume (mm^3^) was calculated via the following formula: V = (W^2^ × L)/2 (V, tumor volume; W, tumor width; L, tumor length). The mice were sacrificed when the control tumors (with PKCη) reached approximately ∼10% of their body weight (∼1500 mm^3^). At the end of the experiment, all the animals were weighed and euthanized, and the liver, lungs, spleen, kidneys, brain, and tumors were harvested. The weights of the tumors and organs were recorded. Portions of the tumors, lungs, liver, and brain were preserved in 4% paraformaldehyde for histological and immunohistochemical analysis. For immunoblot analysis, a small portion of the dissected tumors was snap-frozen in liquid nitrogen. Tumor tissue samples (25 mg) were homogenized in RIPA buffer (500 μL) containing protease and phosphatase inhibitors, followed by sonication for 5 min. Homogenized tissue samples were centrifuged at 13,000 × *g* for 25 min at 4 °C, and the supernatants were collected and subjected to immunoblot analysis.

In the MDA-MB-231 xenograft model, whole-body images of luciferase expression in NSG mice were acquired via the Xenogen In Vivo Imaging System (Xenogen). After the mice were anesthetized, 200 μL of D-luciferin (15 mg/mL) (no. LUCK-1G, Gold Biotechnology) was injected intraperitoneally, and imaging analysis was performed in vivo. Luciferase expression data were quantified via Living Image software (version 4.7.3) in a fixed region of interest.

To evaluate the direct prometastatic role of PKCη independent of primary tumor growth effects, mice (*n* = 6) injected with 4T1 control or PKCη^KO^ cells (1 × 10^6^) were monitored and euthanized when the primary tumor volumes reached comparable sizes between the groups. At the end of the experiment, all the animals were weighed and euthanized, and the liver, lungs, spleen, kidneys, brain, and tumors were harvested. The weights of the tumors and organs were recorded. The lungs were fixed in Bouin’s solution (HT101320-1 L, Sigma), and visible metastatic nodules were counted macroscopically and recorded. Portions of the lung tissue were preserved in 4% paraformaldehyde for histological analysis. The sections were stained with hematoxylin and eosin (H&E) and examined via light microscopy. The metastatic burden (metastasis score) was quantified via ImageJ software (NIH). Individual metastatic lesions were manually outlined via freehand or polygon selection tools, and their regions of interest were measured. The total metastatic area was calculated by summing all individual lesion areas. The entire lung tissue area was subsequently measured by outlining the complete lung tissue boundary. The metastasis score was calculated as a percentage.^72^ Data are presented as the mean ± SEM (*n* = 6 per group). Statistical significance was determined via two-way ANOVA, with significance levels set at *P < 0.05, **P < 0.01, ***P < 0.001, and ****P < 0.0001.

### Human TNBC tissue microarray (TMA) and IHC staining of PKCη

**A** TNBC tissue microarray (BR1301a) was obtained from Tissue Microarray. Com LLC (Derwood, MD, USA), containing 130 cases/130 cores with a single core per case, including pathology grade, TNM classification, clinical stage (AJCC 8th edition), and IHC results (ER, PR, HER2+). The array comprised 122 TNBC cases with 6 positive (ER+, PR+, and HER2+) and 2 normal breast tissue controls. TMA slides (5 μm thick) were stored at 4 °C and baked at 60 °C for 60 min before staining to prevent tissue detachment during antigen retrieval.

The tumor sections were deparaffinized and rehydrated following standard protocols. Antigen retrieval was performed by incubating the slides in 10 mM citric acid buffer (pH 6.0) at 100 °C for 20 minutes. Endogenous peroxidase activity was blocked with 0.3% H₂O₂, followed by blocking with 0.1% Tween and 5% bovine serum albumin for 1 h at room temperature. The sections were incubated with a primary antibody against PKCη (rabbit polyclonal PKCη antibody; NBP2-38711; NOVUS Biologicals). Detection was performed via a VECTASTAIN ABC Kit (Cat #PK-6200, Vector Laboratories) according to the manufacturer’s instructions.

Images were captured (PANNORAMIC MIDI scanner), and PKCη-positive cells were quantified for each tissue core. The results are expressed as the percentage of positively stained cells (QuPath software) (Supplementary Table 5).

### Immunohistochemistry

Formalin-fixed, paraffin-embedded blocks of tumor samples were sectioned at a thickness of 5 μm via a fully automated rotary microtome (no. RM2255, Leica), dried for 1 h at 65 °C, deparaffinized, and rehydrated. The slides were incubated in 10 mM citric acid buffer (pH 6) at 100 °C for 20 min for antigen retrieval. Endogenous peroxidase activity was blocked with H_2_O_2_ (0.3%). The sections were then blocked for 1 h at room temperature with blocking solution (0.1% Tween and 5% bovine serum albumin), followed by incubation with primary antibodies against Ki67 (Abcam, #ab15580), PKCη (Santa Cruz, #sc215), YAP (CST, #14074) and pYAP (Ser127) (CST, #13008). VECTASTAIN ABC Kits (Cat #PK-6200, Vector Laboratories, Inc.) were used for detection according to the manufacturer’s protocol.

Images were captured via a PANNORAMIC MIDI scanner (3DHISTECH) and analyzed with QuPath software (version 0.2.1). For each tissue, regions of interest were marked, and individual cells were automatically detected and classified as positively or negatively stained on the basis of the intensity of the DAB channel (brown staining). The number of positive nuclei and the total annotated area were calculated for PKCη, YAP, pYAP Ser127, and Ki67 expression. The results are expressed as the percentage of positively stained cells within each selected tissue area. The quantification of positive cells was performed consistently across all samples to ensure standardized quantification. The average percentage of positively stained cells was calculated and represented graphically as expression scores.^73^

### METABRIC-BRCA patient data analysis

#### Correlation analysis

Correlation analysis of *PRKCH* and *YAP1* was performed on the Breast Cancer (METABRIC)^27,74^ dataset via cBioPortal (https://www.cbioportal.org/).

#### Distant metastasis-free survival (DMFS)

The DMFS analysis was performed via the KMPLOT Webserver. The patient samples whose expression was above the median from different probes of the same gene were added to the probes of *YAP1* (213342_at) and *PRKCH* (206099_at), with the best cutoff for autoselection. The ER status (IHC), PR status (IHC), and HER2 status array results were considered negative.

### TCGA-BRCA patient data analysis

#### Correlation analysis

Correlation analysis of *PRKCH* and *YAP1* was performed via Gepia3 (https://gepia3.bioinfoliu.com/) in the TCGA-BRCA (TCGA-Tumor, TCGA-Peritumor and GTEx-normal reference) datasets using Spearman correlation coefficient. Furthermore, the correlations of *PRKCH* and *YAP1* with the GPCR isoforms *GNAQ/GNAi1, GNA12, GNA13*, and *GNAI1* were also determined via GEPIA3.

#### Overall survival (OS)

Overall survival (OS) data for *YAP1* and *PRKCH* were generated from the KMPLOT webserver (https://kmplot.com/analysis/). The analysis was restricted to a cohort of 313 breast cancer patients who had not received any endocrine or chemotherapy treatment in the TCGA-BRCA datasets.

#### Prognostic implications

Analysis of the prognostic impact of high-YAP/high-*PRKCH* vs high-YAP/low-*PRKCH* tumors was performed on the TCGA-BRCA dataset downloaded from cBioPortal (https://www.cbioportal.org/). Patients in the upper 25th percentile of *YAP* mRNA expression were considered the high-*YAP* patient group. The high-YAP patient group was further stratified into high PRKCH (upper 25th percentile) and low PRKCH (lower 25th percentile) groups. The survival data were uploaded to the KMPLOT webserver (https://kmplot.com/analysis/) for generation of a survival plot and calculation of the hazard ratio.

### Immunoblot analysis

Whole-cell extracts were prepared by lysing the cells in RIPA lysis buffer containing 10 mM Tris (pH 8.0), 100 mM NaCl, 5 mM EGTA (pH 8.0), 45 mM 2-mercaptoethanol, 1% NP-40, 10 mM EGTA (pH 8.0), 50 mM NaF, and 0.1% SDS. Protease inhibitors (1 mM PMSF, 10 μg/ml aprotinin, and 10 μg/ml leupeptin) and phosphatase inhibitors (1 mM sodium orthovanadate, 50 mM β-glycerophosphate, and 5 mM sodium pyrophosphate) were added immediately prior to cell lysis. The lysates were incubated on ice for 30 min and sheared several times via a 21-gauge needle. The cell lysates were centrifuged at 13,000 × *g* for 25 min at 4 °C, and protein concentrations were determined via the Bio-Rad (Hercules, CA) protein assay. Aliquots of 30–60 μg of protein were prepared and resolved by electrophoresis on 10–15% polyacrylamide gels using Bio-Rad Mini-PROTEAN II cells. Proteins from the gel were electroblotted onto PVDF (Bio-Rad) in Bio-Rad Mini Trans-Blot transfer cells. After 1 h of blocking with 3% BSA in PBS at 37 °C, the PVDF membranes were incubated with the indicated primary antibodies overnight at 4 °C, followed by incubation with HRP-conjugated secondary antibodies. Immunoreactive protein bands were visualized via an enhanced chemiluminescence (ECL) reagent (Biological Industries) and detected via a Gel Doc system (Bio-Rad). Quantification was performed via ImageJ software. The antibodies used in this study were against YAP (CST, #14074), MST1 (CST, #3682), pMST1 (CST, #3681), LATS1 (CST, #3477), 14-3-3 ζ/δ (CST, #7413), pLATS1 (CST, #8654), pYAP (Ser127) (CST, #13008), pYAP (Ser109) (CST, #53749), pYAP (Ser397) (CST, #13619), pTAZ (Ser89) (CST, #75275), TAZ (CST, #83669), PTEN (CST, #9559) Lamin B1 (CST, #13435), AKT (pan) (CST, #4691), AXL (CST, #8661), Pan-TEAD (CST, #13295), CYR61 (CST, 14479), IGFBP3 (CST, #25864), HA-Tag (CST, #3724), FLAG (CST, #14793), pAKT (Ser473) (CST, #4060), and Anti-rabbit IgG (CST, # 7074). Epithelial–Mesenchymal Transition (EMT) Antibody Sampler Kit, CST, #9782 [(Vimentin (D21H3), Snail (C15D3), Slug (C19G7), ZEB1 (D80D3), N-cadherin (D4R1H), ZO-1 (D7D12), E-cadherin (24E10)]. pYAP (Ser128) (Thermo Fisher Scientific, # PA5-117264), PKCη (Santa Cruz, #sc215), PKCε (BD Biosciences, #610085), PKCδ (BD Biosciences, #610398), PKCα (BD Biosciences, #610108), PKCζ (Abcam #ab59364), PKCη (Abcam #ab179524), EpCAM (Abcam, ab223582), and β-actin (ICN Biomedicals Inc., 691001) were used.

### Coimmunoprecipitation

#### Co-IP of PKCη with a mouse monoclonal antibody against YAP1

Five hundred microliters of 3% BSA was used to precoat the protein G beads (#17061801, Cytiva, Sweden). Protein G beads (200 μL) were preabsorbed with mouse monoclonal anti-YAP (CST, #12395) (5 µL/sample) for 90 min at 4 °C. Excess antibodies were removed via wash buffer (25 mM Tris/HCl, pH 7.5; 150 mM NaCl; 5 mM EDTA; and 1% Triton X-100). PKCη scrambled control cell lysates (2–2.5 mg/sample) of MDA-MB-231 or 4T1 cells were added to the bead‒antibody complex and incubated at 4 °C overnight. The cells were lysed in RIPA buffer without SDS. The immune complexes were washed extensively with wash buffer, followed by the addition of SDS sample buffer. The corresponding samples were electrophoresed on 10% polyacrylamide gels and immunoblotted with an anti-PKCη antibody (Abcam, #ab179425). An anti-mouse IgG1 isotype control (#02-6100; Invitrogen) was used.

#### Reverse Co-IP of YAP1 with a rabbit monoclonal antibody against PKCη

After precoating with 3% BSA, protein A beads (Cytiva, #17078001) and protein G beads (Cytiva, #17061801) (200 μL) were preabsorbed with anti-PKCη (#ab179425, Abcam, UK) (10 µL/sample) for 90 min at 4 °C, followed by washing with wash buffer to remove unbound antibody. PKCη scrambled control cell lysates (2–2.5 mg/sample) of MDA-MB-231 or 4T1 cells were added to the bead‒antibody complex and incubated at 4 °C overnight. The immune complexes were processed (as mentioned above) and immunoblotted with an anti-YAP antibody. An anti-rabbit IgG isotype control (#ab37415; Abcam, Cambridge, UK) was used.

#### Co-IP of 14-3-3 with a mouse monoclonal antibody against YAP1

Following precoating with 3% BSA, protein G beads (200 μL) were preabsorbed with mouse monoclonal anti-YAP (CST, #12395) (5 µL/sample) for 90 min at 4 °C, and excess antibody was removed via wash buffer. PKCη scrambled control or knockout cell lysates (2–2.5 mg/sample) of 4T1 cells were added to the bead‒antibody complex and incubated at 4 °C overnight. The immune complexes were washed extensively with wash buffer, followed by the addition of SDS sample buffer. The corresponding samples were electrophoresed on 10% polyacrylamide gels and immunoblotted with an anti-14-3-3 ζ/δ antibody (CST, #7413). An anti-mouse IgG1 isotype control antibody was used as the control.

### Kinase assay

To analyze the phosphorylation of YAP at position Ser128 by PKCη, we used a kinase assay kit from Promega (ADP-Glo^TM^ Kinase Assay, #V6930). To analyze the kinase activity of PKCη with recombinant YAP, different concentrations of YAP (from 0.6 to 1 µg/mL) were tested against active PKCη at a concentration of 1 µg/mL. To confirm specific phosphorylation at the Ser128 site, we constructed a small peptide representing the YAP moiety with Ser128, comprising 15 amino acids. The peptides were designed as follows: wild-type peptide (wild-type: QHVRAHSSPASLQLG) and S128A mutant peptide (Mut: QHVRAHS**A**PASLQLG). We performed kinase assays using wild-type and mutant peptides in conjunction with active PKCη to assess the specific phosphorylation potential of PKCη at the YAP-Ser128 site.

### Statistical analysis

The statistical significance of the differences between the experimental groups was determined via the unpaired two-tailed t-test and two-way ANOVA test of variance in cases of multiple variables (using GraphPad Prism software, version 10.6.0). The data are presented as the means ± SEMs. Statistical significance is shown as *P < 0.05, ** *P* < 0.01, ***P < 0.001, and ****P < 0.0001.

### Ethics declarations

The in vivo protocols and experiments were approved by the Institutional Animal Ethics Committee and Israel Ministry of Health (Protocol # IL-17-03-2019 (E) and IL-2412-111-5 (E)).

## Supplementary information

Supplementary file

Table S1

Table S2

Table S3

Table S4

Table S5

## Data Availability

All the data supporting the conclusions are included in the manuscript and supplementary materials.
