# Supplementary material for: PKC-eta promotes breast cancer metastasis by regulating the Hippo–YAP signaling pathway
Source: Signal Transduct Target Ther. 2026 Feb 17;11:58. doi: 10.1038/s41392-026-02572-0 (PMC12910040; doi:10.1038/s41392-026-02572-0)
Supplement: Supplementary file 1 — Supplementary file [file 41392_2026_2572_MOESM1_ESM.docx]

Supplementary Materials for

**PKC-eta promotes breast cancer metastasis by regulating the Hippo–YAP signaling pathway**

Vijayasteltar B Liju^1^, Kamran Waidha^1^, Amitha Muraleedharan^1,2^, Divya Ram Jayaram^1,3^, Hodaya Haimov^1^, Sankar Jagadeeshan^1^, Dinesh Babu Manikandan^1^, Raghda Abu Shareb^1^, Livingstone Nurukurti^1^, Menachem Sklarz^1^, J Silvio Gutkind^5^, Irit Allon^6,7^, Ofir Cohen^1,4^, Moshe Elkabets^1*^, and Etta Livneh^1*^

^*^Correspondence to: Prof. Etta Livneh ([etta@bgu.ac.il](mailto:etta@bgu.ac.il)), Prof. Moshe Elkabets ([moshee@bgu.ac.il](mailto:moshee@bgu.ac.il)).

**This PDF file includes:**

Materials and Methods

Supplementary Figures. S1 to S26

Supplementary Tables S1 to S7

**Supplementary materials and methods**

**Relative expression of PKCη among BC subtypes**

We used METABRIC whole-transcriptome data^1^ available across the cohort (n=1980), with a clinical table that specified the intrinsic subtype for each sample. Box plots were generated via the ggplot2 R package (v3.5.1). Statistical significance was assessed via Welch's t test.

The TCGA and METABRIC expression matrices were filtered to retain the genes present in both datasets. For both datasets, zero values were replaced with missing values, quantile normalization was applied, and a log₂ transformation was performed. The expression within each sample was then centered and scaled to unit variance. The datasets were then combined, and batch effects were corrected via ComBat [PMID: 22257669].^2^ A gradient-boosted tree binary classifier, xgboost [arXiv:1603.02754],^3^ was trained on METABRIC data (70% training, 30% internal test split) to distinguish claudin-low samples from other samples. The optimal number of boosting rounds was determined via 5-fold cross-validation. The AUC for the trained model was 0.98 (for the test set). The trained model was then applied to TCGA data to assign probabilities for claudin-low status, with thresholds of <0.2 (“claudin-low”), >0.8 (“not claudin-low”), and intermediate values (“unknown”). The hormone receptor status of TCGA BC tumors was obtained from Lehmann *et al*. [PMID: 34725325].^4^

**Colony formation assay**

The *in vitro* cell survival assay is based on the ability of a single cell to grow into colonies. 4T1 and MDA-MB-231 cells (control and PKCη^KO^) were detached and resuspended by trypsinization. A total of 1000 cells were seeded into each well of a 6-well plate. The cells were then incubated in a 5% CO_2_ environment at 37°C. The experiment was independently repeated three times. After 7 days, the plates were washed with PBS, fixed with formaldehyde (1%), and stained with crystal violet solution (0.5 g of crystal violet; 0.05% w/v crystal violet). Air-dried dishes and digital images of the colonies were obtained via a camera. Colonies were counted via ImageJ imaging analysis software.

**Soft agar assay**

4T1, MDA-MB-231 control, and PKCη^KO^ cells in complete growth medium with 0.6% agar were layered onto 1% agar beds in twelve-well plates; complete medium supplemented with 10% FBS was added to the cells, and the medium was replaced with fresh medium twice a week for 15 days. The cells were incubated with 200 μl of nitroblue tetrazolium chloride solution per well overnight at 37°C. Once the colonies were stained, photographs of the wells were taken via an imager, and the colonies were counted via image analysis software.

**Cell viability and proliferation assays.**

For the cell proliferation assays, 4T1, MDA-MB-231 scrambled control (CRISPR/Cas9), and their respective PKCη^KO^ cells were seeded (5000 cells/well) in 96-well plates. The cells were trypsinized, collected, and counted via an automated cell counter (Countess II FL, Thermo Scientific, Inc.) at intervals of 24 h for five days. A minimum of four wells were prepared at each time point. The data are representative of three independent experiments.

**Anoikis assay.**

Anoikis resistance in the tumor cell lines 4T1 and MDA-MB-231 (control and PKCη^KO^) was determined via low-adherence and reattachment assays. The cells grown in flasks were detached via trypsin/EDTA and then seeded (5000 cells/well) in low-attachment and normal-attachment 96-well plates in media supplemented with 10% FBS. The cells were maintained in low-adherence plates for 48 h and then transferred by pipetting the contents of each well into normal adherence plates for 16 h. The cells seeded in a normal adherence plate were maintained under these conditions for 48 h, with an additional 16 h. At the end of the assay, a 2,3-bis-(2-methoxy-4-nitro-5-sulfophenyl)-2H-tetrazolium-5-carboxanilide (XTT) assay was performed according to the manufacturer’s instructions (no. 20–300-1000, BI). The plates were immediately read on a Multiskan Spectrum Reader (Thermo Fisher Scientific). Cells seeded in low-attachment and normal-attachment 96-well plates were assayed by XTT to determine the optical density (OD) of the cell number versus the absorbance for all the cell lines. Each experiment was repeated at least three times in triplicate. Relative cell survival and reattachment were calculated from the mean OD of the normal attachment plate and the mean OD of the low attachment plate.

**Wound healing (scratch) assays.**

4T1, MDA-MB-231, and PKCη^KO^ cells were seeded in 24-well plates and grown to confluency. A gap in the confluent monolayer (middle of the well) was created via a sterile pipette tip (200 μL) followed by two washes with PBS. Subsequently, medium containing low serum (0.1% FBS) was added, and the wound area was photographed (three images per well at the indicated time points) at 4× magnification with an I X 70 Olympus optical light microscope at different time points, such as 0, 12, 24 and 48 h. The wound areas were measured and normalized to time 0 using ImageJ software (1.53i).

**Invasion and migration assays.**

4T1 and MDA-MB-231 cells (CRISPR control and PKCη^KO^) (50,000 cells/well) were seeded into the upper chambers in FBS-free DMEM. The lower chambers were filled with DMEM (10% fetal bovine serum). To assay invasive potential, 50,000 cells (4T1, MDA-MB-231, and their respective PKCη^KO^) were seeded into growth factor-reduced Matrigel invasion chambers (ThinCertsTM, 24-well, Greiner Bio-One, 8 μm pore). After 24 h, the cells were stained with crystal violet solution. The cells in the lower chamber of the membrane were counted and quantified. Fields of view were randomly chosen, and the average cell number was determined.

**Analysis of Gene Expression Omnibus (GEO) datasets for stemness**

**The** Gene Expression Omnibus datasets GSE235703 and GSE86861 were analyzed via Geo2R to evaluate the expression of *PRKCH* in spheroid cells enriched with the CSC marker OCT4.

**Mammosphere culture (sphere formation assay)**

For sphere formation assays, cells were plated at a density of 5,000 cells/well in 6-well plates in serum-free DMEM/F12K at 37°C in a 5% CO_2_ atmosphere. The medium was supplemented with 20 µg/mL epidermal growth factor (EGF), 10 µg/mL basic fibroblast growth factor (bFGF), 10 µg/mL insulin, 0.4 g/100 mL bovine serum albumin (BSA), 10 µL/mL L-glutamine, and 1% penicillin/streptomycin. To prevent cell adhesion, the wells were coated with a sterile solution of poly(2-hydroxyethyl methacrylate) (10 g/L in 95% ethanol; Sigma‒Aldrich, St. Louis, MO, USA). The cells were cultured in mammosphere medium and observed for 7–10 days. Spheres with a diameter of at least 50 µm were counted and designated spheres.

**Spheroid invasion assay**

Approximately 5,000 cells were seeded in poly(2-hydroxyethyl methacrylate) (poly-HEMA)-coated 6-well plates using specialized mammosphere medium. After seven days, the formed spheroids were collected for the subsequent sphere migration assay. For the migration assay, the spheroids were mixed with Matrigel and plated in 24-well plates^5^. Images of spheroid migration were captured via an inverted microscope at 24 h after spheroid seeding. The cell migration area was measured and analyzed via ImageJ software.

**Limited dilution assay**

For the limited dilution assay (LDA), different dilutions of cells (100, 50, 10, 5, and 1 cell per well) were seeded into 96-well plates coated with poly(2-hydroxyethyl methacrylate) and cultured in mammosphere medium. Each cell concentration was seeded in 10 wells. The data shown are aggregated from two independent experiments. Colony formation was visually assessed.^5^ After 10 days of culture, the number of wells containing spheres was counted, and the sphere formation frequency (SFF) was calculated via extreme limiting dilution assay (ELDA) software (ELDA; [https://bioinf.wehi.edu.au/software/elda/](https://protect.checkpoint.com/v2/r02/___https://bioinf.wehi.edu.au/software/elda/___.YzJlOmJlbmd1cmlvbnVuaXZlcnNpdHlvZnRoZW5lZ2V2OmM6bzplN2FjY2QwOGE0YWZmYjJkYzNkYTk0MWExMTQzZDIyNjo3Ojc0NDE6ZWRiZWM0YjRkNzRiZTQ3MjhjNmQwNTdmZmFlYjMzNTg5NWNiZjM1OTMwNTFjOTIxY2M0ODlkZjY4ZDlhM2Y0NDpwOlQ6Tg)).

**Flow cytometric analysis of the CD44^high^ and CD24^low^ phenotypes**

4T1 and MDA-MB-231 cells, along with their PKCη^KO^ cells, were cultured in specialized mammosphere medium. The spheroids were subsequently dissociated into single cells by using trypsin. For flow cytometric analysis, the cells were stained with primary antibodies against CD44 and CD24. The following day, the cells were fixed with 4% paraformaldehyde (PFA) for 20 min and permeabilized with PBS containing 0.2% (w/v) Triton X-100. The cells were blocked for 30 min at 37°C with 5% bovine serum albumin (BSA) and subsequently incubated at 37°C for 1 h with anti-CD44 and anti-CD24 primary antibodies. After incubation, the cells were washed three times with 1× PBS and incubated for 1 h at room temperature in the dark with the corresponding secondary fluorescent antibodies. Following incubation, the cells were washed three times with 1× PBS and analyzed by flow cytometry via a FACSAria III (BD Biosciences). The antibodies used were against CD44 (Santa Cruz, #sc7297) and CD24 (Santa Cruz, #sc19585).

**ALDEFLUOR assay**.

ALDEFLUOR™ (Stemcell, Cat# 01700) analysis was performed according to the manufacturer’s protocol. Briefly, 4T1 and MDA-MB-231 cells, along with their PKCη^KO^ cells, were cultured in specialized mammosphere medium. Single-cell suspensions were counted from the spheres, washed with PBS, and stained with substrate solutions with or without DEAB. ALDEFLUOR-stained samples were washed, resuspended in staining buffer, and loaded for analysis. FACS was performed using a FACSAria III flow cytometer (BD Biosciences).

**Immunofluorescence**

Briefly, the cells were fixed in 4% paraformaldehyde for 10 min, washed in PBS, permeabilized with Triton 0.1% for 10 min, and blocked in antibody diluent solution for 1 h. Antigen recognition was performed via incubation with primary antibodies against PKCη (NBP2--38711, Novus Biologicals), YAP (CST, #14074), E-cadherin (CST, #3195), EpCam (Abcam, #ab223582), vimentin (CST, #5741), TAZ (CST, #83669), Sox2 (CST, #23064), and Nanog (Santa Cruz, 1E6C4, sc-293121) overnight at 4°C and with goat anti-mouse/rabbit Alexa Fluor 488/546 (Thermo Fisher Scientific) as the secondary antibody for 1 h at room temperature. The nuclei were counterstained with DAPI.

The coverslips were mounted on slides via DAKO mounting medium and imaged via an Olympus FluoView FV1000 laser scanning confocal microscope. Immunofluorescence images represent single 2D confocal sections. For the sphere confocal image, we used a 35 mm glass-bottom dish (Cellvis, #D35-20-1.5-N). Multiple optical sections were acquired throughout the z-axis of the spheres via a confocal microscope and merged to generate composite 3D images. Confocal images were analyzed via ImageJ software. Regions of interest (ROIs) were manually outlined around individual cells. The mean gray value (average pixel intensity) was measured for each ROI to quantify the fluorescence intensity. Background fluorescence was measured in cell-free areas and subtracted from each cellular measurement to obtain corrected fluorescence values. The results are expressed as the average fluorescence intensity/cell.

**Survival study of 4T1-injected female NSG mice.**

4T1 cells (4 × 10^6^) were injected into the tail vein of female NSG mice in a total volume of 100 µL. The mice were observed daily, and their body weights were measured every other day to assess the intensity of metastasis. Survival data were recorded daily, and the probability of survival was calculated from the collected data. In addition, the survival probabilities of the control group and the group of mice injected with PKCη^KO^ cells were compared. Statistical significance was determined via survival curve comparison (log-rank (Mantel‒Cox) test).

At the end of the experiment, the lungs were harvested and photographed. The lungs were fixed in Bouin's solution, and visible metastatic lung nodules were counted and recorded. The lung tissues were fixed in 4% paraformaldehyde for histological analysis. Lung tissue sections were stained with hematoxylin and eosin (H&E) and examined under a light microscope. The metastatic burden (metastasis score) was quantified by dividing the total area of metastatic lesions in the lungs by the total lung tissue area.^6^ Micronodule foci were quantified across multiple sections by counting the number of microscopic lesions in the lung tissue of each animal. The data are presented as the means ± SEMs, with statistical significance determined by two-way ANOVA (*P < 0.05, **P < 0.01, ***P < 0.001, ****P < 0.0001).

**Rescue of PKCη expression in MDA‑MB‑231 PKCη knockout cells to assess EMT, YAP‑Hippo signaling, and the metastatic potential of PKCη.**

To confirm the specific role of PKCη in TNBC metastasis, rescue experiments were performed by re-expressing PKCη in MDA-MB-231 PKCη^KO^ cells via lentiviral transduction. PKCη expression was verified by western blot analysis prior to functional assays.

*PRKCH overexpression in different cell lines*: In this study, we performed rescue experiments by reintroducing *PRKCH* (VB250407-1299xzc) and *PRKCH* dead kinase (K384A & K387R) (VB250407-1302feh) expressing lentivirus plasmids ordered from VectorBuilder Inc. Lentiviruses were prepared from HEK293FT cells grown to 80% confluency in 100 mm plates. In total, 8 μg of plasmid vector and 10 μg of packaging plasmid (5 μg of plp1, 2 μg of plp2, and 3 μg of VSV-G) were transfected into HEK293FT cells via polyethylenimine (PEI) reagent (no. 23966–1; Polysciences, Inc.). The medium was changed after 24 h, and the lentiviruses were collected after 2 days. The supernatant containing the lentivirus was centrifuged at 500 × g for 5 min to remove the dead cells. The supernatant was filtered through a 0.45 μm filter, aliquoted, and stored at -80°C for further use. A fresh culture of MDA-MB-231 cells was then infected with a 1:1 ratio of virus suspension to complete DMEM supplemented with 10% FBS. Positive clones were isolated via hygromycin selection (500 μg/ml). Similarly, we overexpressed PRKCH in MCF7 cells.

*In vitro functional assays*: Migration and invasion assays were performed via Boyden chamber assays as previously described. EMT markers and Hippo–YAP pathway components were analyzed via western blotting to assess molecular restoration. The total YAP protein levels and phosphorylation status at Ser128, Ser109, Ser397, and Ser127 were evaluated to confirm pathway reactivation.

*In vivo rescue experiments*: MDA-MB-231 control, MDA-MB-231 PKCη^KO^, and PKCη-rescued cells were injected subcutaneously into the mammary fat pads of 6–8-week-old female NSG mice (n=6 per group). Primary tumor growth was monitored by measuring the tumor volume on alternate days via digital calipers. Bioluminescence imaging was performed to track primary tumor growth and metastatic dissemination.

At the end of the experiments, the mice were sacrificed, and the primary tumors and organs (brain, lung, liver, spleen, and kidney) were harvested and weighed. Relative organ weights were calculated via normalization to body weight. Bioluminescence imaging was performed on the harvested organs to quantify the organ-specific metastatic burden. The mean luminescent intensity was measured at the regions of interest (ROIs) and expressed in pixels. The data are presented as the means ± SEMs (n=6 per group). Statistical significance was determined via two-way ANOVA, with significance levels set at *P < 0.05, **P < 0.01, ***P < 0.001, and ****P < 0.0001.

**Histological (H&E) staining**

Paraffin-embedded tissues were sectioned (5 μm) before deparaffinization and rehydration. The tissue sections were stained with hematoxylin for 3 min, washed with tap water to remove excess stain, counterstained with eosin for 30 s, dehydrated, and equilibrated with xylene. The tissues were then dehydrated and mounted (Surgipath, Sub-X, Mounting Medium).

**siRNA-mediated gene silencing**

siRNA transfection was performed as previously described.^7^ Briefly, for transient silencing of YAP1, cells were transfected via GenMute siRNA Transfection Reagent (SignaGen, SL100568) according to the manufacturer's protocol, with a siRNA nontargeting control sequence (siRNA Control) (IDT, 51-01-14-04) and *YAP1* gene targeting sequences (IDT; hs.Ri.*YAP1*.13.1, hs.Ri.*YAP1*.13.2 and hs.Ri.*YAP1*.13.4 for humans and mm.Ri.*YAP1*.13.1, mm.Ri.*YAP1*.13.2 and mm.Ri.*YAP1*.13.4 for mice). PKCη knockdown was performed via predesigned siRNA sets from Med Chem Express (MCE). *PRKCH* Human (Cat. No. HY-RS11131) and mouse (Cat. No. HY-RS11132) predesigned siRNAs were used for MDA-MB-231 and 4T1 cells, respectively. Each set contained three designed siRNAs (5 nmol each, HPLC purified) and a control. Following preliminary screening of all three siRNAs, the two most effective sequences (the first two sets from humans and mice) were selected for subsequent experiments. The cells were harvested after 48 h for western blot analysis.

For migration and invasion assays, transfected cells were harvested by trypsinization 48 h post transfection, counted, and further utilized for Boyden chamber-based migration and invasion assays as described in the above sections.

**Protein–Protein Docking**

To understand the interaction between YAP1 and PKCη, a protein–protein docking approach was used. Owing to the lack of a complete PKCη and YAP1-Tead binding domain (TBD) crystal structure, a homology-based modeling approach was used.^8^ The PKCη structure was generated via Swiss-Modeler (https://swissmodel.expasy.org/). The YAP1-TEAD binding domain (TBD) was modeled via IntFOLD (https://www.reading.ac.uk/bioinf/IntFOLD/). The generated structures were validated via a SWISS-MODEL structure assessment (https://swissmodel.expasy.org/assess). Both structures were prepared and optimized prior to docking. All the structural inconsistencies were eliminated. Protein‒protein docking was performed via the online Hdock server (http://hdock.phys.hust.edu.cn).

**ELISA for protein‒protein binding analysis**

The YAP protein used in the ELISAs was obtained from OriGene, Germany (TP325864), and the recombinant PKCη protein was obtained from Abcam (#ab60849-5). Biotinylated secondary antibodies were obtained from Cell Signaling Technology (Anti-rabbit IgG, HRP-linked Antibody, #7074), as were the primary antibodies against YAP (rabbit mAb, #14074) and PKCη from Abcam (#ab179524). ELISA plates were purchased from Thermo Scientific (#464718). The assay plates were emptied and incubated with 100 µL/well blocking buffer (PBS with 2% bovine serum albumin) for 1 h at room temperature with shaking. The blocking buffer was aspirated, and the plates were washed three times with 200 µL/well of PBST. YAP (1 µg/mL in PBS) was added to the plates at a volume of 50 µL/well and incubated overnight at 4°C. The plates were then washed three times with 200 µL/well PBST. Next, 50 µL of various concentrations of active PKCη (ranging from 0.1 to 1 µg/mL in PBS) were added to 96-well ELISA plates per well. The plates were incubated at room temperature for 1 h and then washed thrice with PBST. The appropriate primary antibody for PKCη, diluted at a ratio of 1:1000, was added to the 96-well ELISA plates at 50 µL per well. The plates were then incubated at room temperature for 1 h, after which they were washed three times with PBST. The corresponding HRP-conjugated secondary antibody (diluted 1:5000 in PBS-T) was subsequently added to the plates, which were subsequently incubated at room temperature for 1 h. After incubation, the plates were washed three times with PBS. After washing, the signal was developed by adding 50 µL/well TMB substrate (KPL, #50-76-00) and incubating for up to 15 min. The reaction was stopped by adding 25 µL/well 1 M phosphoric acid. The absorbance at 450 nm was recorded via a plate reader, and the data were analyzed and fitted via GraphPad Prism (version 10.5.0).

**Analysis of the cytoplasmic/nuclear fraction**

A cell fractionation kit (Nuclear and Cytoplasmic Extraction Reagents, Thermo Fisher Scientific) was used to isolate the cytoplasmic and nuclear fractions according to the manufacturer's instructions. Western blot analysis was performed using specific antibodies against PKCη and YAP, together with loading controls: β-actin for the cytoplasmic fraction and lamin B for the nuclear fraction. Densitometric analysis of the bands was performed via ImageJ software to quantify relative protein expression. The expression levels of PKCη and YAP were compared between the cytoplasmic and nuclear fractions, and the results were interpreted on the basis of the relative localization of these proteins. Statistical significance was determined via appropriate statistical tests (two-way ANOVA) to evaluate differences between the control and PKCη^KO^ groups.

**Cycloheximide (CHX)-induced protein stability assay**

MDA-MB-231 and 4T1 cells and their PKCη^KO^ cells were cultured in the appropriate growth medium. The cells were treated with cycloheximide (CHX) at a concentration of 10 µg/mL for 1, 2, 4, 6 or 12 h to assess protein stability over time. After treatment, the cells were harvested via cell lysis buffer containing protease inhibitors. The cell lysates were subjected to western blot analysis with antibodies against YAP. Images of the blots were captured, and the data were analyzed for significance via ImageJ or GraphPad Prism software. The concentration of the YAP target protein was normalized to that of the loading control, β-actin. The results were interpreted on the basis of the relative expression of YAP at different time points after CHX treatment in both the control and PKCη^KO^ cells. Statistical significance was determined between the control and PKCη^KO^ groups.

**Coexpression of YAP–PKC isoforms**

In this study, we coexpressed YAP1 (pcDNA Flag Yap1 #18881) with PKCη & PKCη (Kinase Dead) and other isoforms of PKCs (pHACE δ and ε) in HEK293FT cells grown to 80% confluency in 145 mm plates. The plasmids were cotransfected at a ratio of 1:1 with 15 μg of plasmid each using polyethylenimine (PEI) reagent (no. 23966–1, Polysciences, Inc.). The ratio of plasmid to PEI was 1:3. The media was changed after 24 hours, and the cell lysates were collected after 2 days for further study.

**Detection of the upstream phosphorylation cascade.**

To investigate the upstream phosphorylation cascade of the Hippo pathway, which is activated by AKT phosphorylation in the absence of PKCη, PKCη-knockout TNBC cells were treated with the AKT inhibitor MK-2206 at a concentration of 10 µg/mL. The cells were incubated for 24 h to ensure effective inhibition of AKT activity. After the treatment period, the cells were washed with cold phosphate-buffered saline (PBS) to remove residual medium. Cell lysates were prepared, and western blot analysis was performed to determine the phosphorylation levels of pAKT, pLATS1, pMST1, YAP (Ser127), and YAP (Ser397). Statistical significance was determined via appropriate tests (t tests or ANOVA) to compare the differences between the treated and untreated groups.

**Quantitative real-time PCR (RT‒qPCR)**

Total RNA was isolated from the cells via an RNA isolation kit (II RNA Mini Kit, Bioline, #BIO-52073) according to the manufacturer's instructions. The isolated RNA was reverse transcribed into cDNA via the qScript cDNA Synthesis Kit (Quantabio, #95047-100). Quantitative real-time PCR was performed via a LightCycler 480 Instrument II (Roche Diagnostics, North America) with SYBR Green PCR Master Mix (AzuraView, #AZ-2305). The sequences of primers used for the specific genes are listed in Supplementary Table 7. Gene expression levels were calculated via the comparative Ct method (2^-ΔΔCt^) with TBP as the internal reference gene. Each sample was analyzed in triplicate, and the experiments were independently repeated at least three times. The relative mRNA levels were calculated.

**Supplementary Figures**

**Figure S1**


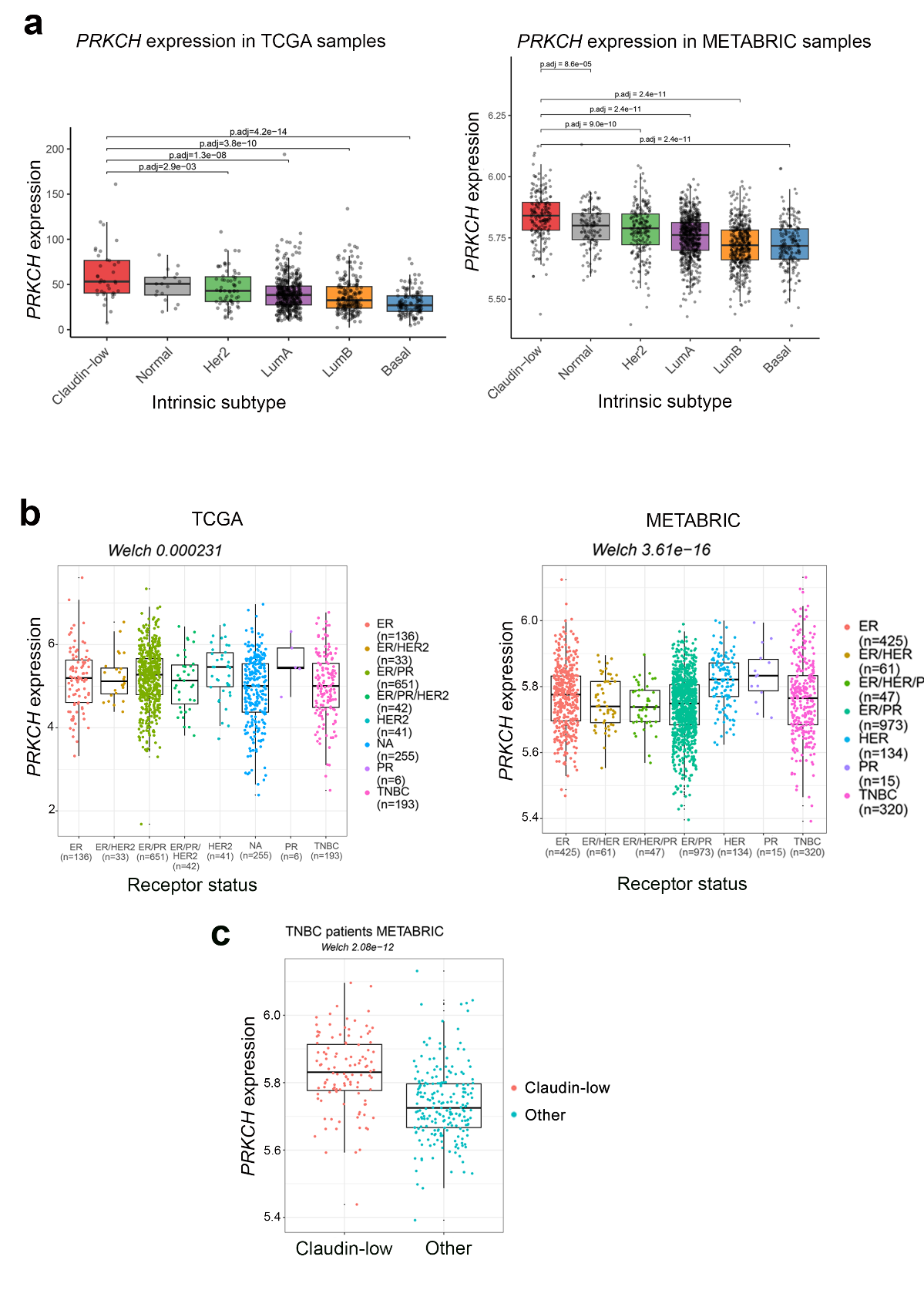


**Supplementary Fig. 1**. **Comparative analysis of *PRKCH* expression across intrinsic molecular subtypes and receptor-defined classifications in METABRIC and TCGA datasets.**

**a**, *PRKCH* expression levels in intrinsic molecular subtypes of the METABRIC and TCGA datasets, demonstrating a significant elevation of *PRKCH* expression in claudin-low tumors compared to other breast cancer subtypes. **b,** Stratification by receptor-defined status (including TNBC vs. non-TNBC) in METABRIC and TCGA revealed that *PRKCH* was not uniquely enriched in TNBC. **c**, *PRKCH* expression was significantly higher in claudin-low tumors compared with non-claudin-low tumors among TNBC patients.

**Figure S2**


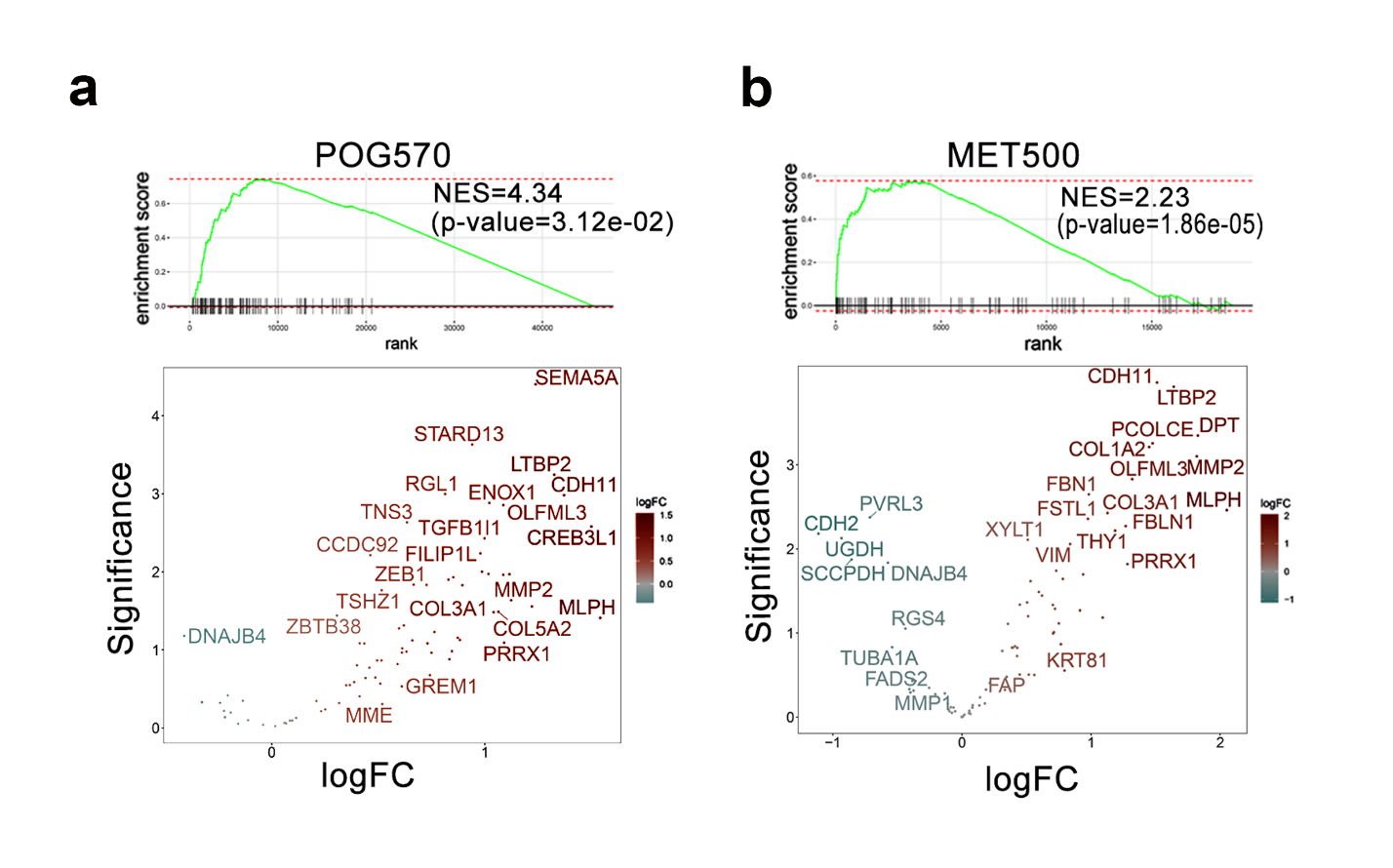


Supplementary Fig. 2. Gene set enrichment analysis demonstrated significant EMT pathway activation with *PRKCH* overexpression.

Gene Set Enrichment Analysis (GSEA) demonstrating significant EMT pathway activation in *PRKCH* overexpression across Metastatic Breast Cancer (MBC) cohorts: (**a**) POG570 and (**b**) MET500.

**Figure S3**


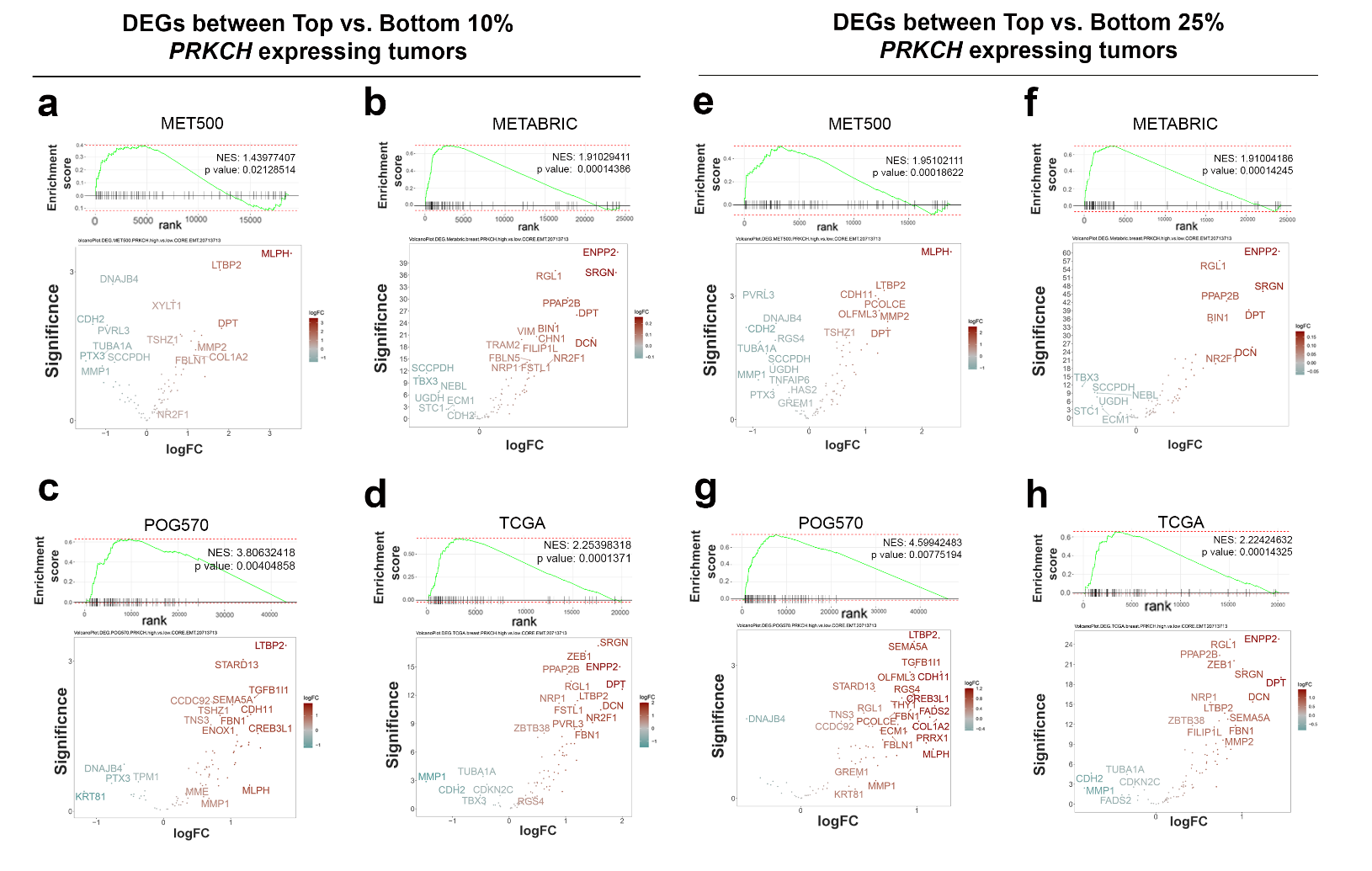


Supplementary Fig. 3. EMT-associated gene expression signature enrichment across multiple BC cohorts (primary and metastatic), with different stratification cutoffs comparing *PRKCH* high vs. low tumors.

These results demonstrate that the EMT gene expression signature was significantly enriched in BC patient groups in different cohorts and with different cutoffs to stratify *PRKCH* expression. The analysis included primary breast cancer cohorts: TCGA, METABRIC, and metastatic breast cancer cohorts: POG570 and MET500 datasets. The EMT signature is based on comparison of highly versus lowly expressing *PRKCH* tumors, with top versus bottom 10% (**a-d**), and top versus bottom 25% (**e-h**).

**Figure S4**


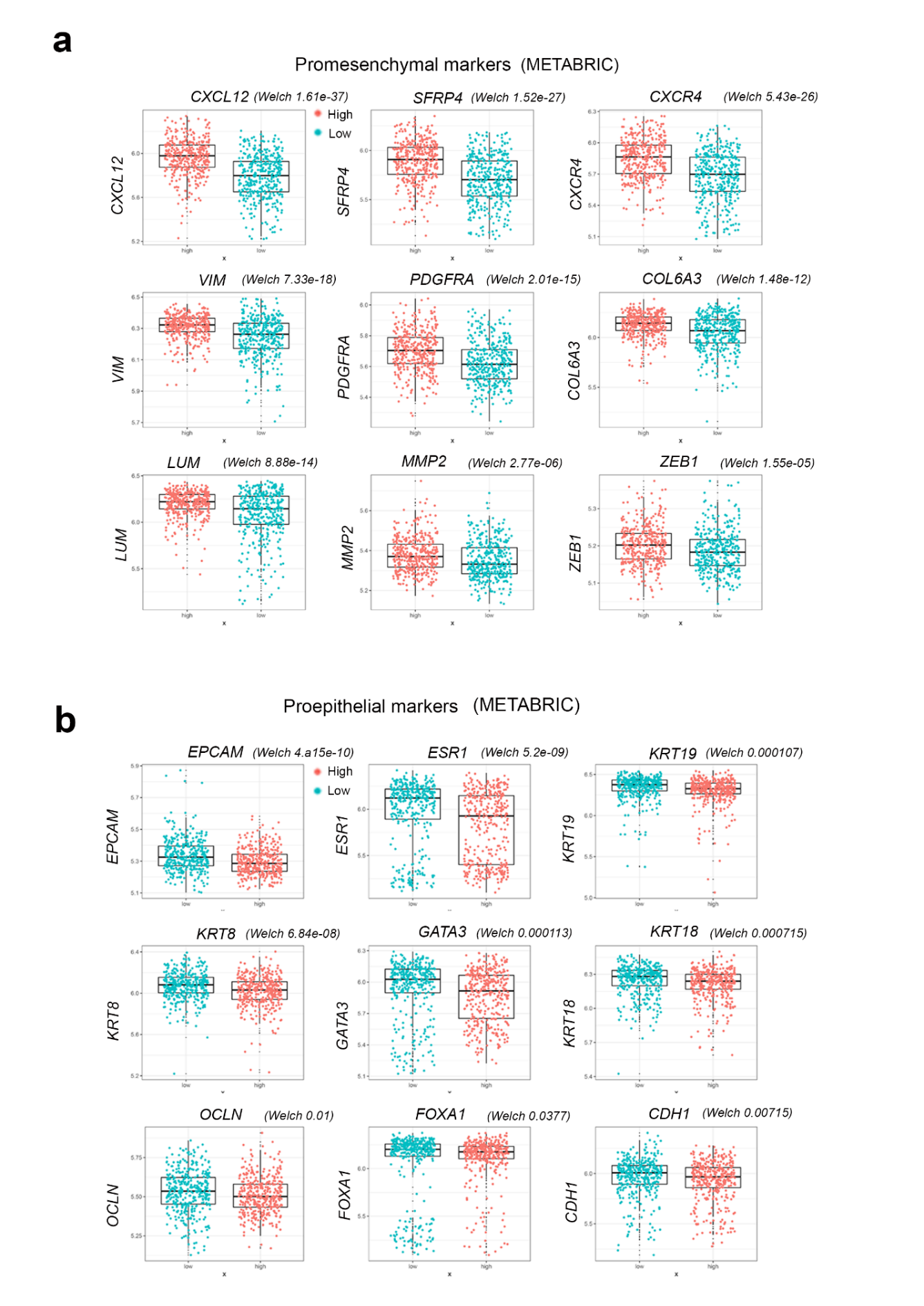


**Supplementary Fig. 4. *PRKCH* expression is associated with different levels of expression for distinct promesenchymal and proepithelial markers in BC.**

Expression analysis of EMT-associated markers in BC tumors with high (red) versus low (blue) PKCη expression**. a,** Expression analysis of promesenchymal markers showed a significant positive association with elevated *PRKCH* in BC. Markers such as *CXCL12, SFRP4, CXCR4, VIM, PDGFRA, COL6A3, LUM, MMP2*, and *ZEB*1 were upregulated in the high *PRKCH* expression group. **b,** Expression of proepithelial markers are associated with low *PRKCH* levels. Markers including *EPCAM, ESR1, KRT19, KRT8, GATA3, KRT18, OCLN, FOXA1*, and *CDH1* were elevated in the low *PRKCH* group.

**Figure S5**


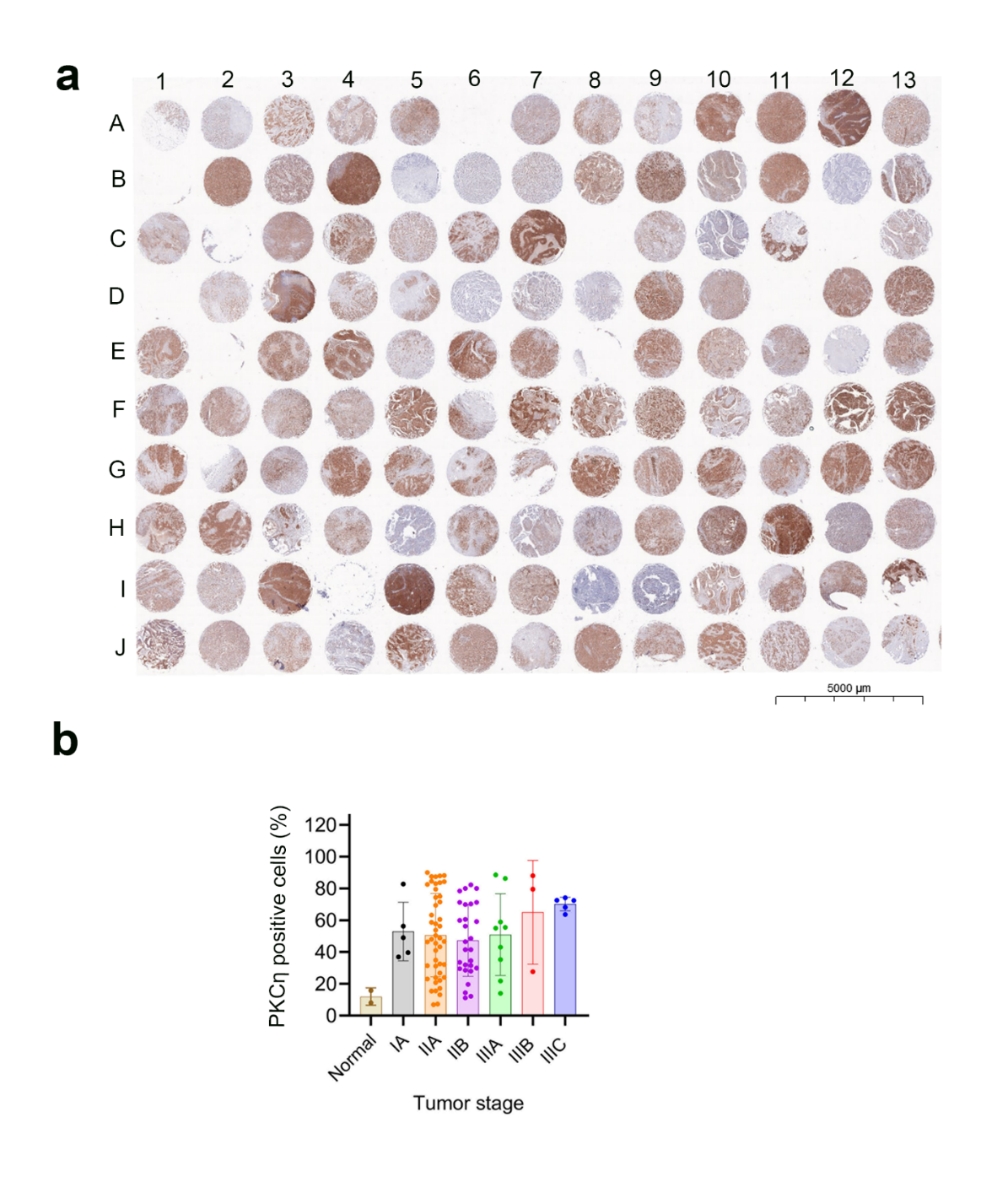


**Supplementary Fig. 5. PKCη expression analysis in TNBC tissue microarray.**

**a**, PKCη protein expression by immunohistochemistry (IHC) in <130 human breast cancer tissue microarray (TMA) samples, including normal breast tissue and TNBC specimens of varying histological grades and clinical stages. **b**, Quantitative analysis of PKCη expression in normal tissues and TNBC grades and stages. The frequency of PKCη-positive cells increased with increasing stage, particularly in advanced-stage TNBC (stages IIIB and IIIC).

**Figure S6**


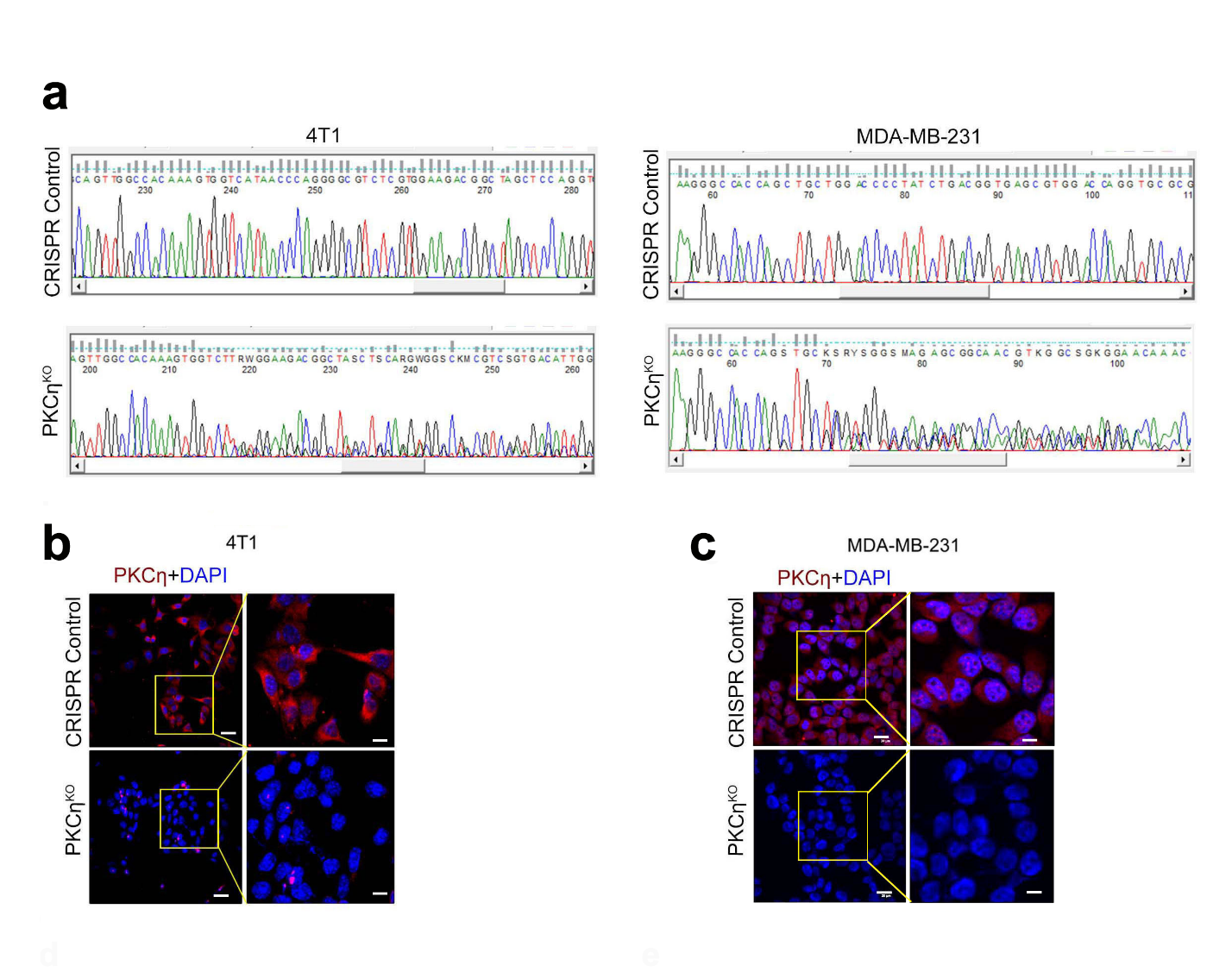


**Supplementary Fig. 6. Validation of PKCη knockout in 4T1 and MDA-MB-231 cells.**

**a,** Sanger sequencing data demonstrating CRISPR/Cas9 editing of PKCη in 4T1 and MDA-MB-231 clones. **b-c,** Representative images showing the expression pattern of PKCη in 4T1 and MDA-MB-231 cells (CRISPR control and PKCη^KO^ clones). The scale bar indicates 20 μm. PKCη was stained red, and cell nuclei were counterstained with DAPI (blue).

**Figure S7**


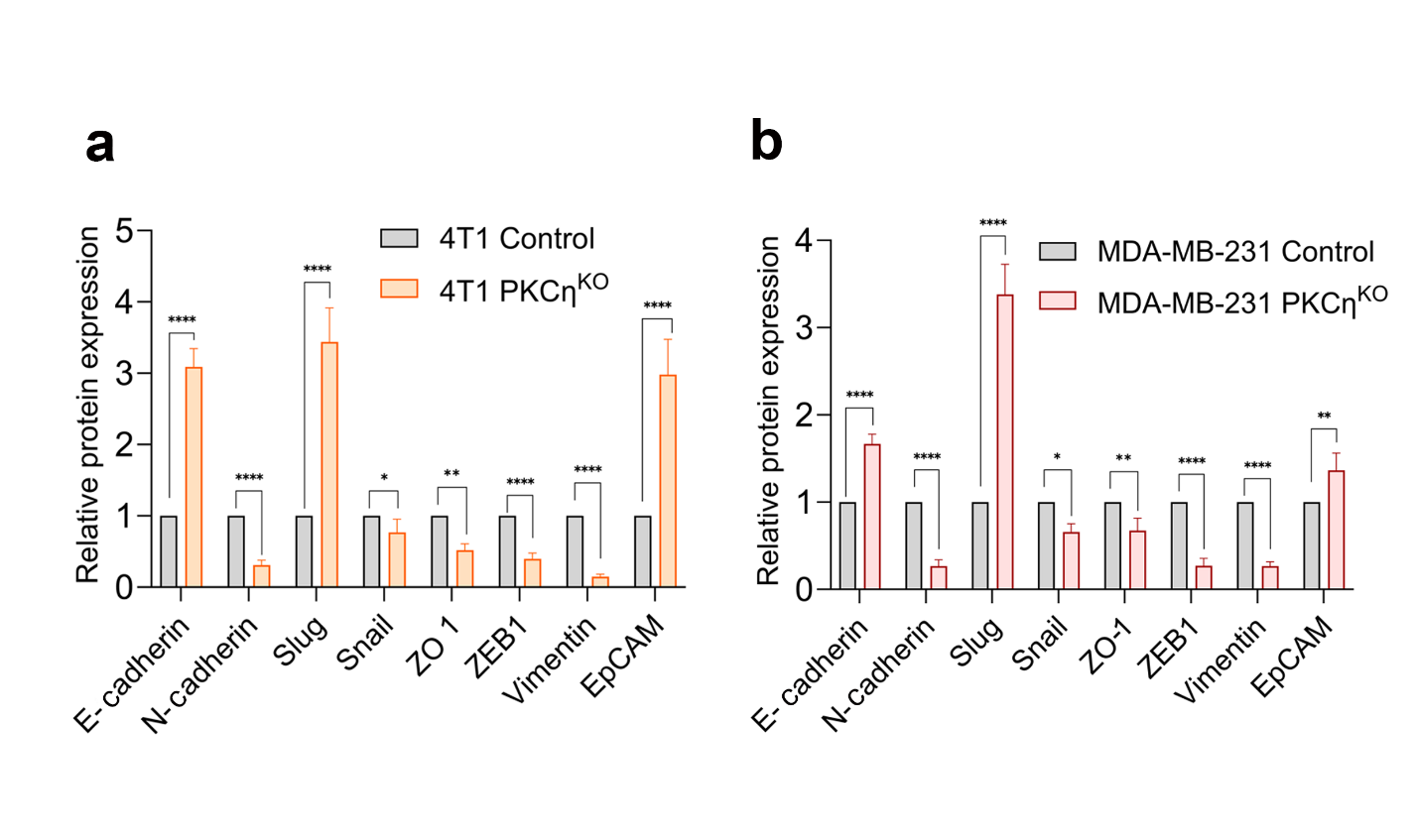


**Supplementary Fig. 7. Quantification of the western blot analysis of EMT markers in 4T1 and MDA-MB-231 cells and their corresponding PKCη^KO^ cells.**

**a-b,** Graphical representation of quantification of western blotting after PKCη knockout in 4T1 and MDA-MB-231 cells showing decreased EMT markers.

**Figure S8**


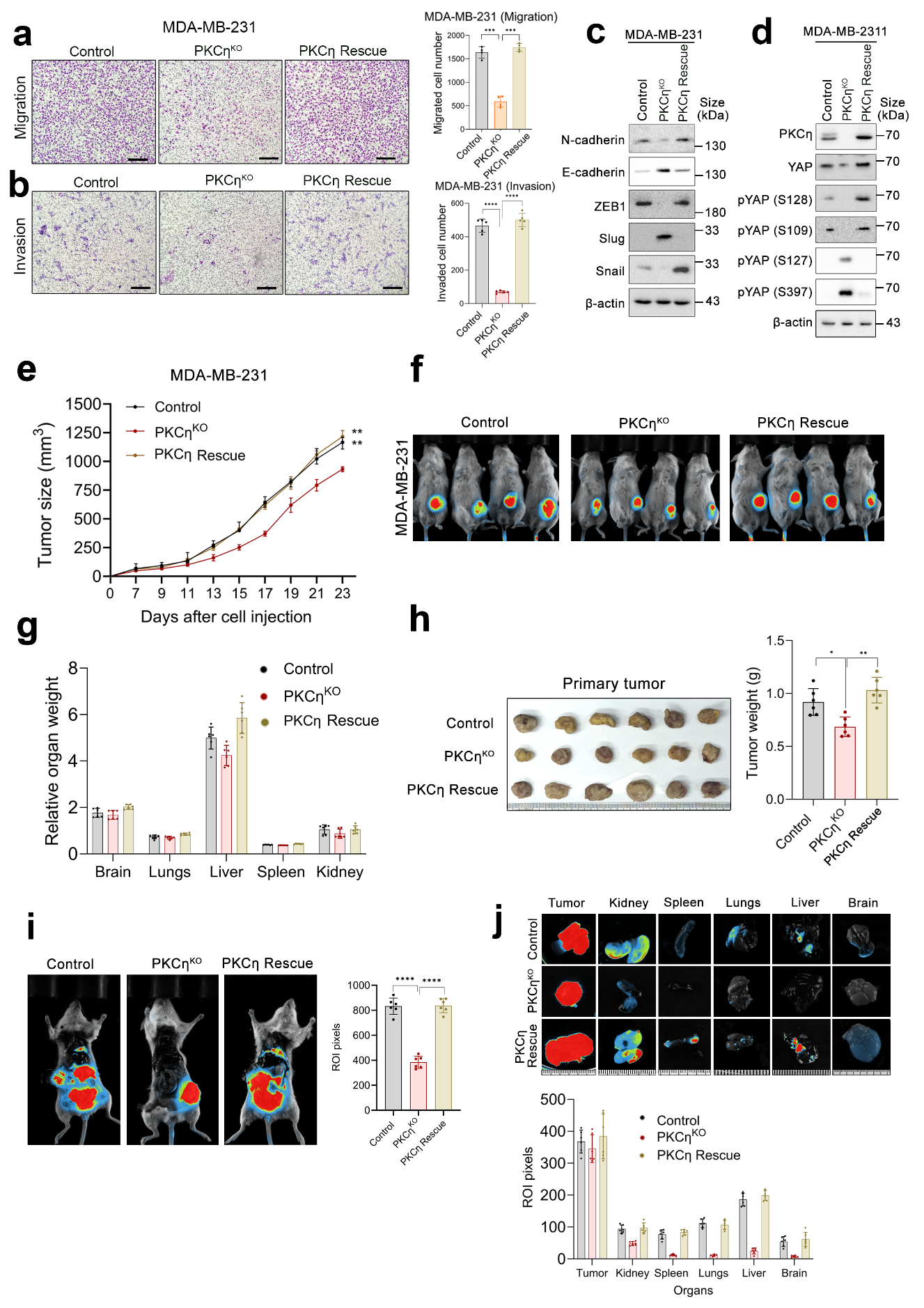


**Supplementary Fig. 8. Rescue experiments demonstrated PKCη-dependent regulation of EMT, migration, and invasion via YAP-Hippo signaling in MDA-MB-231 cells.**

**a and b,** Reduced migratory and invasive abilities of PKCη^KO^ cells were enhanced after re-expression of PKCη. The scale bar indicates 100 μm.  **c,** Re-expression of PKCη in MDA-MB-231 PKCη^KO^ cells restored the expression of EMT markers. **d,** Re-expression of PKCη in MDA-MB-231 PKCη^KO^ cells restored the expression levels of total YAP and its phosphorylation at S128 and S109, as well as reduced phosphorylation at S397 and S127. **e,** PKCη re-expression in MDA-MB-231 PKCη^KO^ cells rescued primary tumor growth in NSG mice (yellow). Tumor growth was assessed by measuring the tumor volume on alternate days. **f,** Representative bioluminescence images of primary tumors in NSG female mice bearing control, PKCη^KO^, and rescued PKCη MDA-MB-231 xenografts. **g,** Relative organ weights of NSG mice xenografted with control, PKCη^KO^, or PKCη-rescued PKCη^KO^ cells. **h,** Primary tumor size comparison and quantification. Images and graphical representation of average tumor mass in control, PKCη^KO^, or PKCη-rescued groups showing restoration of primary tumor size (n=6 animals per group). **i, Representative bioluminescence images showing** the primary tumor and metastatic spread in MDA-MB-231 control, PKCη^KO^, and PKCη-rescued xenografts. The bar diagram shows quantified mean luminescence intensity in the region of interest (ROI) in pixels. **j,** Bioluminescence imaging of organ-specific metastatic burden in control, PKCη^KO^, and PKCη-rescued MDA-MB-231 xenografts, with quantification of the mean luminescence intensity across multiple organs at ROIs in pixels. Data represent mean ± SEM (n=6). Statistical significance was determined using two-way ANOVA, where *P < 0.05, **P < 0.01, ***P < 0.001, and **** P < 0.0001.

**Figure S 9**


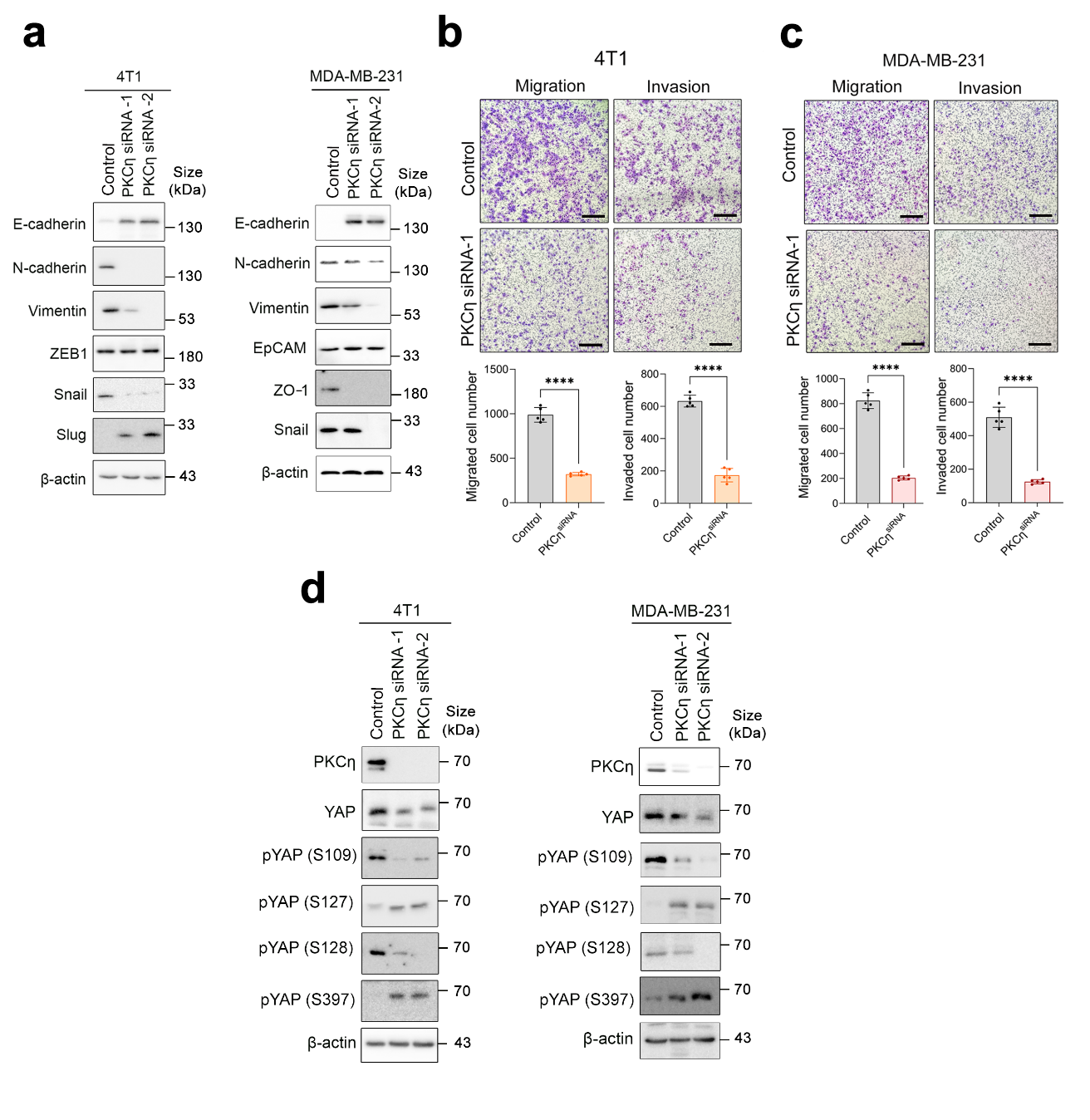


**Supplementary Fig. S9. PKCη silencing reduces EMT and migration through the YAP-Hippo pathway.**

**a,** Expression of EMT markers decreased upon PKCη silencing using siRNA, indicating a shift toward an epithelial phenotype. EMT marker analysis of MDA-MB-231 cells showed reduced mesenchymal marker expression following PKCη silencing. **b** and **c,** Migration and invasion assays revealed a significant reduction in the migratory and invasive capacities of 4T1 and MDA-MB-231 cells following PKCη silencing using siRNA. The scale bar indicates 100 μm. **d,** Western blot analysis of 4T1 and MDA-MB-231 cells following siRNA-PKCη silencing demonstrates decreased YAP expression and S128 phosphorylation (associated with nuclear accumulation and activation), along with elevated phosphorylation of YAP S127 (indicative of inactivation via cytoplasmic retention) and YAP S397 (linked to proteasomal degradation).

**Figure S10**


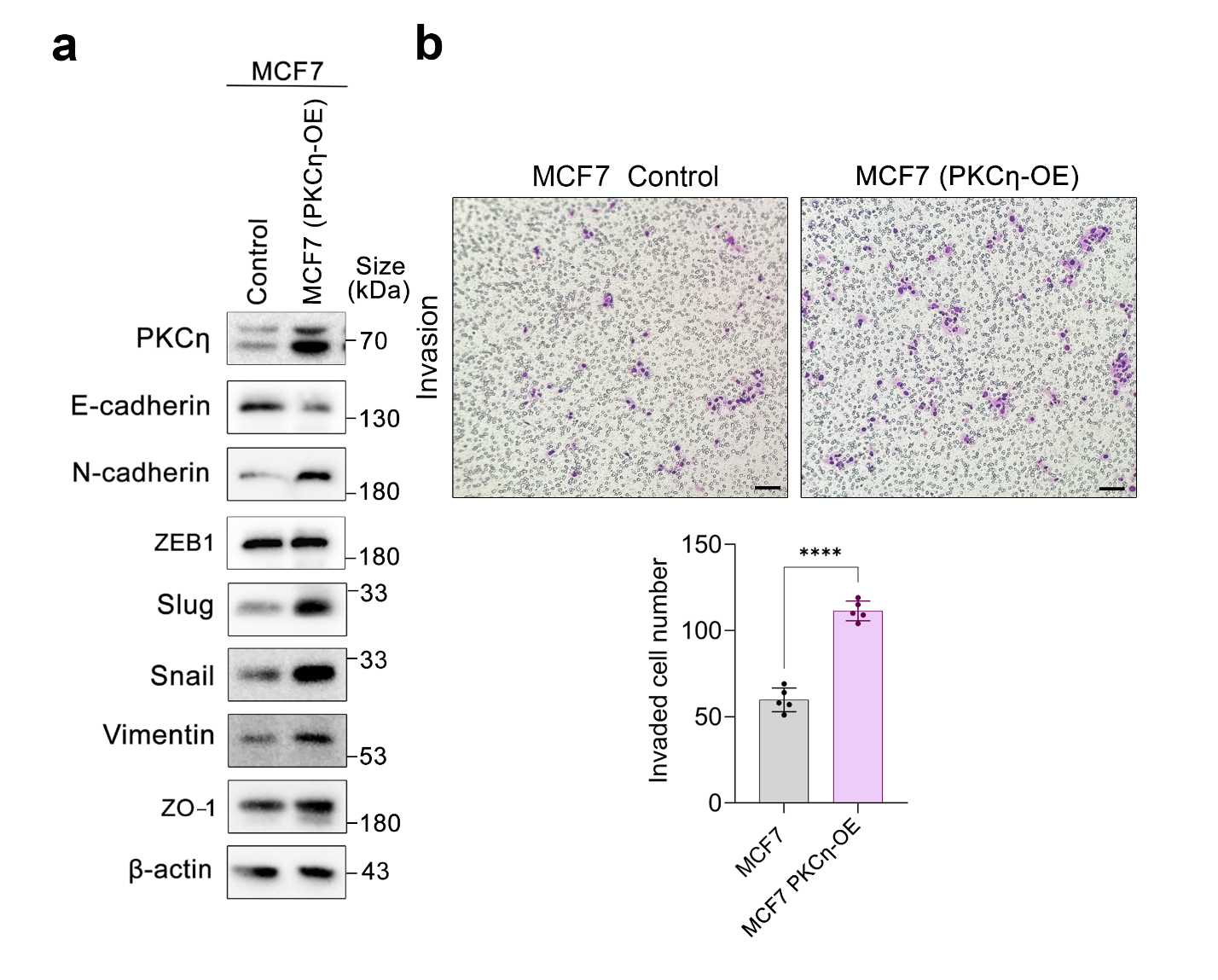


**Supplementary Fig. 10. PKCη overexpression induced EMT markers and enhanced the invasion of luminal non-aggressive MCF7 BC cells.**

**a,** PKCη overexpression (PKCη-OE) in luminal non-aggressive MCF7 BC cells shows an increase in EMT markers. **b,** Invasion ability was significantly increased in MCF7 PKCη-OE BC cells. The scale bar indicates 100 μm.

**Figure S11**


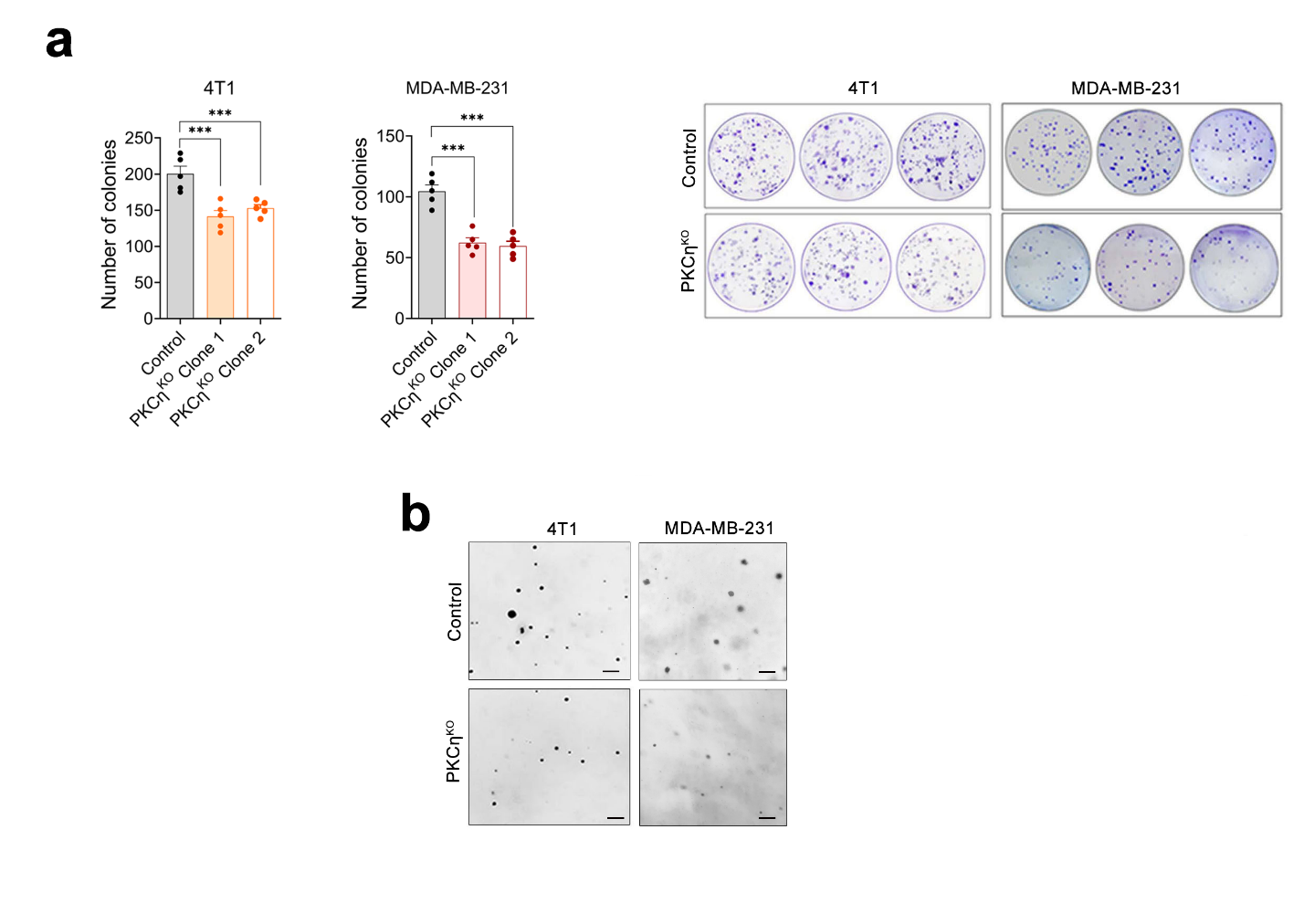


**Supplementary Fig. 11. 4T1 and MDA-MB-231 PKCη^KO^ cells exhibit reduced colony formation and anchorage-independent growth.**

**a,** Representative images and quantification showing the reduced colony-forming ability of PKCη^KO^ cells in both the 4T1 and MDA-MB-231 cell lines. **b,** Representative image showing the reduced ability of PKCη^KO^ cells to form colonies in soft agar in 4T1 and MDA-MB-231 cells (quantitation of this figure is shown in Fig. 2c). The scale bar indicates 100 μm. Data are presented as mean ± SEM. Significance was determined using two-way ANOVA. *P < 0.05, **P < 0.01, ***P < 0.001, and ***p<0.0001.

**Figure S12**


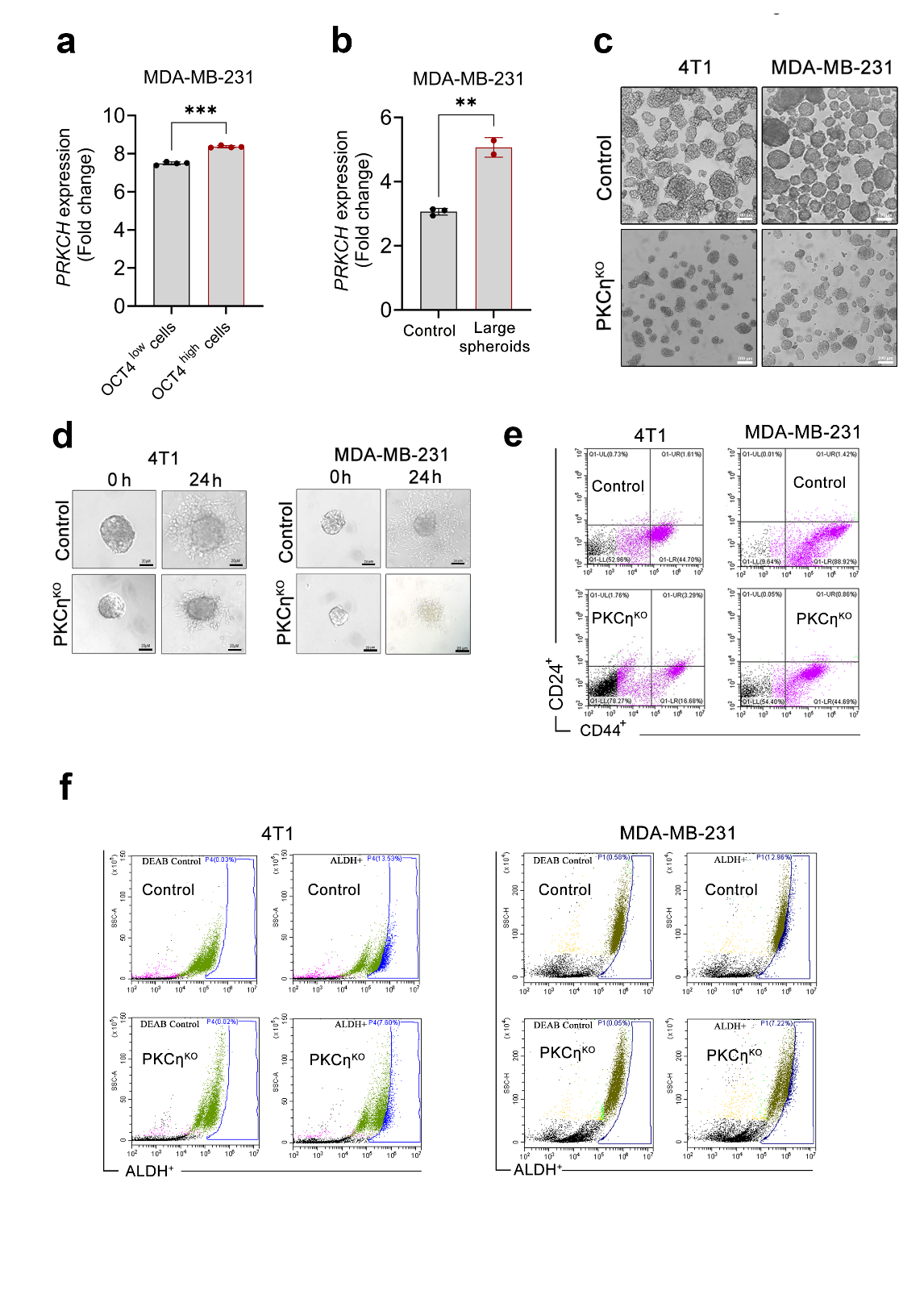


**Supplementary Fig. 12. PKCη regulates spheroid formation, invasion, and stemness in MDA-MB-231 cells.**

**a,** Gene Expression Omnibus (GEO) dataset analysis showing elevated *PRKCH* expression in MDA-MB-231 cells with high *OCT4* expression. **b,** GEO dataset analysis showing increased *PRKCH* expression in larger MDA-MB-231 spheroids. **c,** Representative bright-field images demonstrating that PKCη contributes to spheroid formation in 4T1 and MDA-MB-231 cells. Cells were cultured in specialized medium under sphere-forming conditions for seven days. The size and number of spheres were decreased in PKCη^KO^ cells (n = 9). The scale bar indicates 100 μm. **d**, PKCη knockout in 4T1 and MDA-MB-231 cells significantly reduced cell migration from spheroids through Matrigel, as quantified after 24 h and shown in the accompanying graph (Fig. 2h, n=5). The scale bar indicates 20 μm. **e,** Representative diagrams showing decreased expression of CD44^+^/CD24^-^ in PKCη^KO^ 4T1 and MDA-MB-231 cells, as determined by flow cytometry (Fig. 2j). **f,** Flow cytometric analysis of ALDEFLUOR activity highlights the difference in stem cell activity between control (13.42±1.26% and 12.14±1.49%) and PKCη^KO^ cells (7.93±0.82 and 7.19±0.87%) in 4T1 and MDA-MB-231 cell lines, respectively (Fig. 2k). Data are presented as mean ± SEM. Significance was determined using two-way ANOVA. *P < 0.05, **P < 0.01, ***P < 0.001, and ***p<0.0001.

**Figure S13**


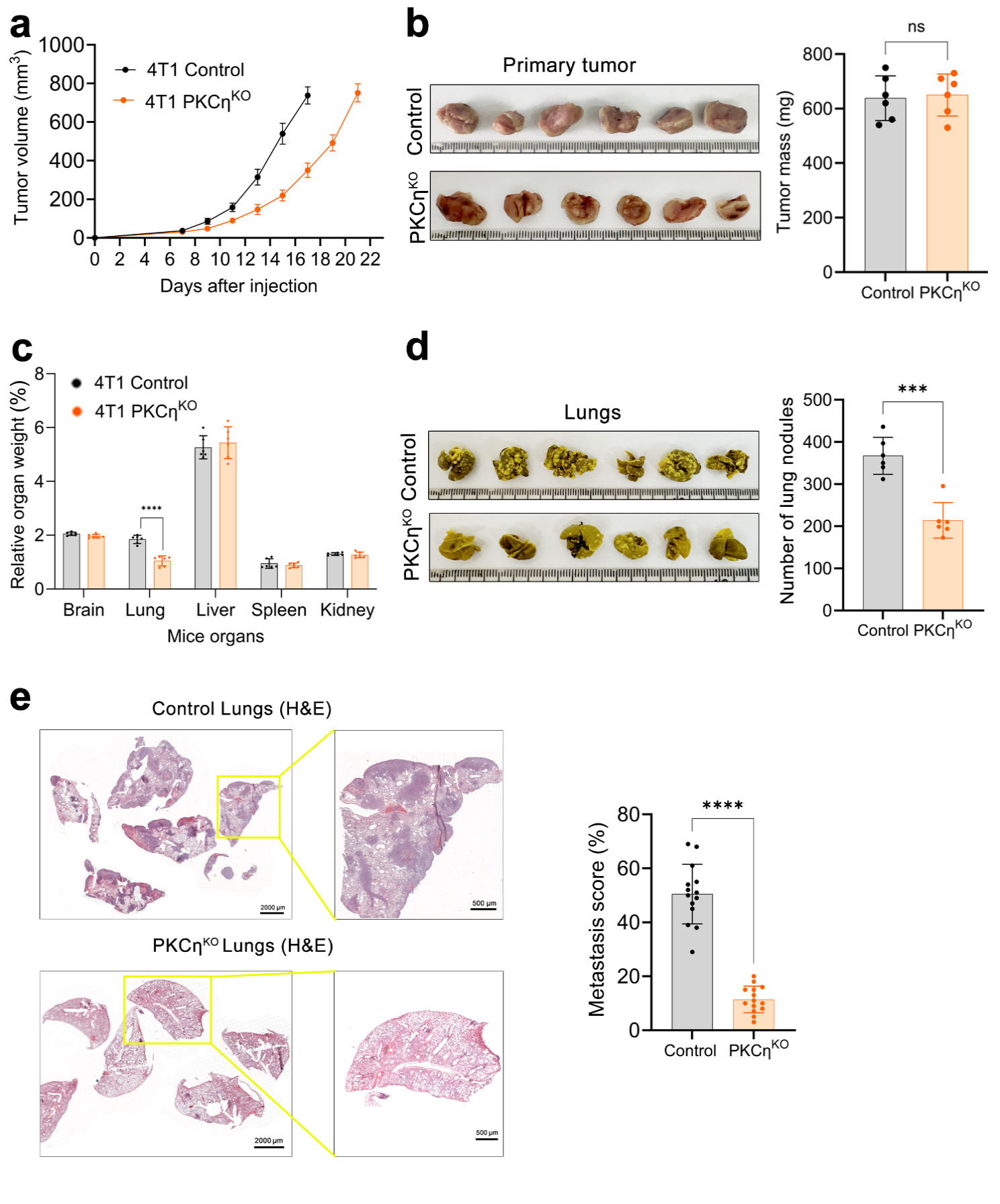


**Supplementary Fig. 13. TNBC PKCη^KO^ cells exhibited reduced levels of metastasis when euthanized at the same tumor size.**

**a,** Comparison of primary tumor growth between control and 4T1 PKCη^KO^ cells xenografted into NSG mice, evaluated by measuring the tumor volume on alternate days. **b,** Images of primary mammary tumors (control and 4T1 PKCη^KO^) collected when the volumes of the primary tumors were similar. **c,** Graphical representation of relative organ weights in the 4T1 control and PKCη^KO^ groups. The relative lung weight increased significantly owing to the abundance of metastatic nodules in the 4T1 control group. **d,** Lung images were collected at the end of the experiment, demonstrating abundant lung metastatic macronodules in control 4T1 mice. Quantitative analysis of metastatic lung nodules in control and 4T1 PKCη^KO^ groups. **e,** Lung tissue sections (control and 4T1 PKCη^KO^) were examined by H&E staining and observed under a light microscope. Graphical representation of lung metastatic burden of control and PKCη^KO^ xenografted mice. Data represent mean ± SEM (n=6). Statistical significance was determined using two-way ANOVA, where *P < 0.05, **P < 0.01, ***P < 0.001, and **** P < 0.0001.

**Figure S14**


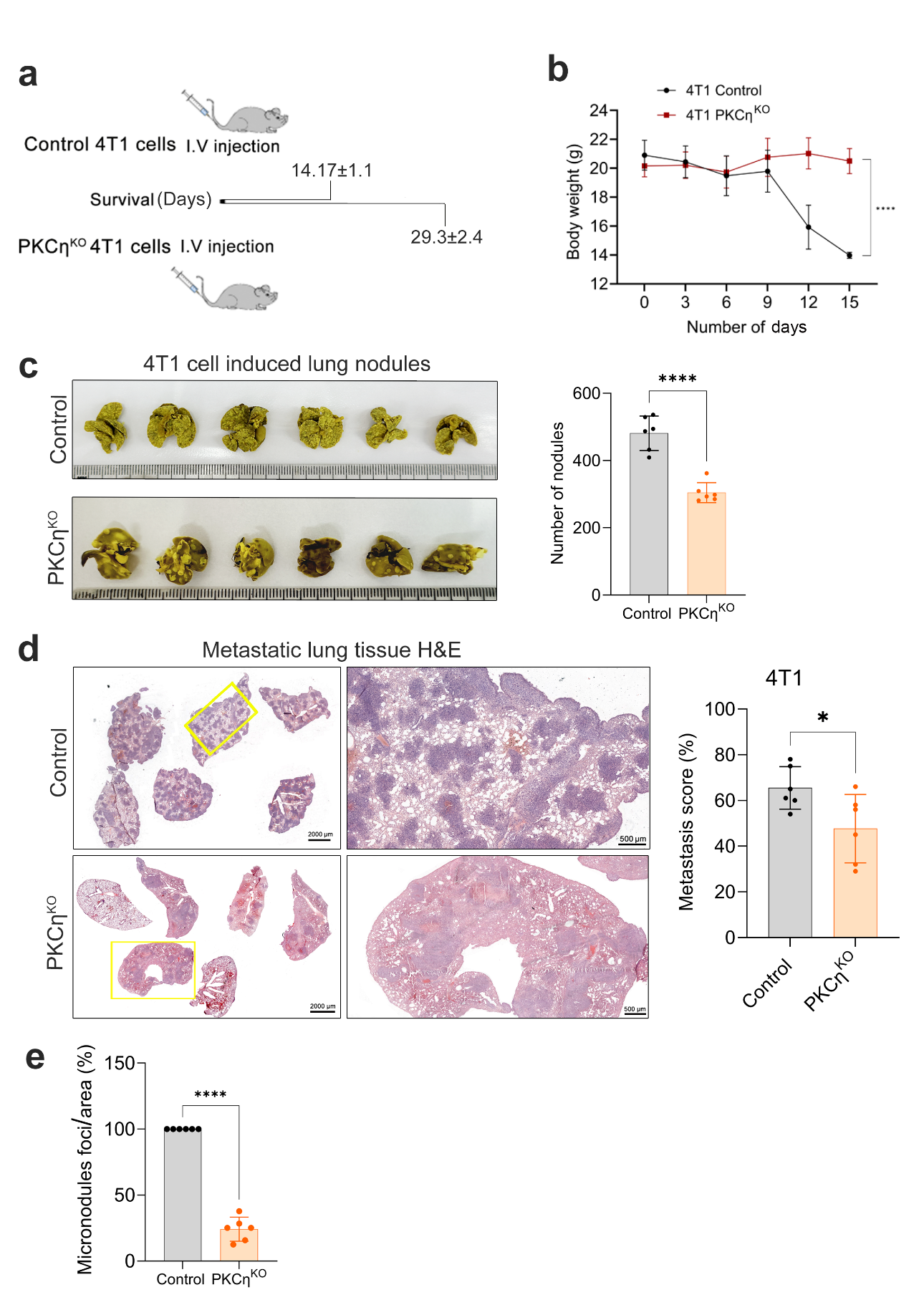


**Supplementary Fig. 14. 4T1 cells with PKCη^KO^ exhibited reduced metastatic potential and mortality in NSG mice.**

**a,** 4T1 control and PKCη^KO^ cells were injected intravenously into female NSG mice via the tail vein, and mouse survival (days) was recorded for mortality analysis (Fig. 3g). **b,** Mice injected with 4T1 PKCη^KO^ cells exhibited an enhanced survival rate and stable body weight, whereas mice injected with control 4T1 cells showed significantly decreased body weight. **c,** Images of the lungs that were collected at the end of the experiment (after 14 and 29 days for control and PKCη^KO^ groups, respectively), demonstrate abundant lung metastatic macronodules in the 4T1 control and PKCη^KO^ injected mice. Quantitative analysis of metastatic lung nodules in both the control and PKCη^KO^ 4T1 injected groups. **d,** Lung tissue (control and 4T1 PKCη^KO^) sections were examined by H&E staining and observed under a light microscope. Graphical representation of the lung metastatic score of control and PKCη^KO^ 4T1 cell-injected mice. **e**, The PKCη^KO^ 4T1 cell-injected mouse group showed significantly decreased micronodule foci compared with the control group. Significance was determined using two-way ANOVA. *P < 0.05, **P < 0.01, ***P < 0.001, ****p<0.0001.

**Figure S15**


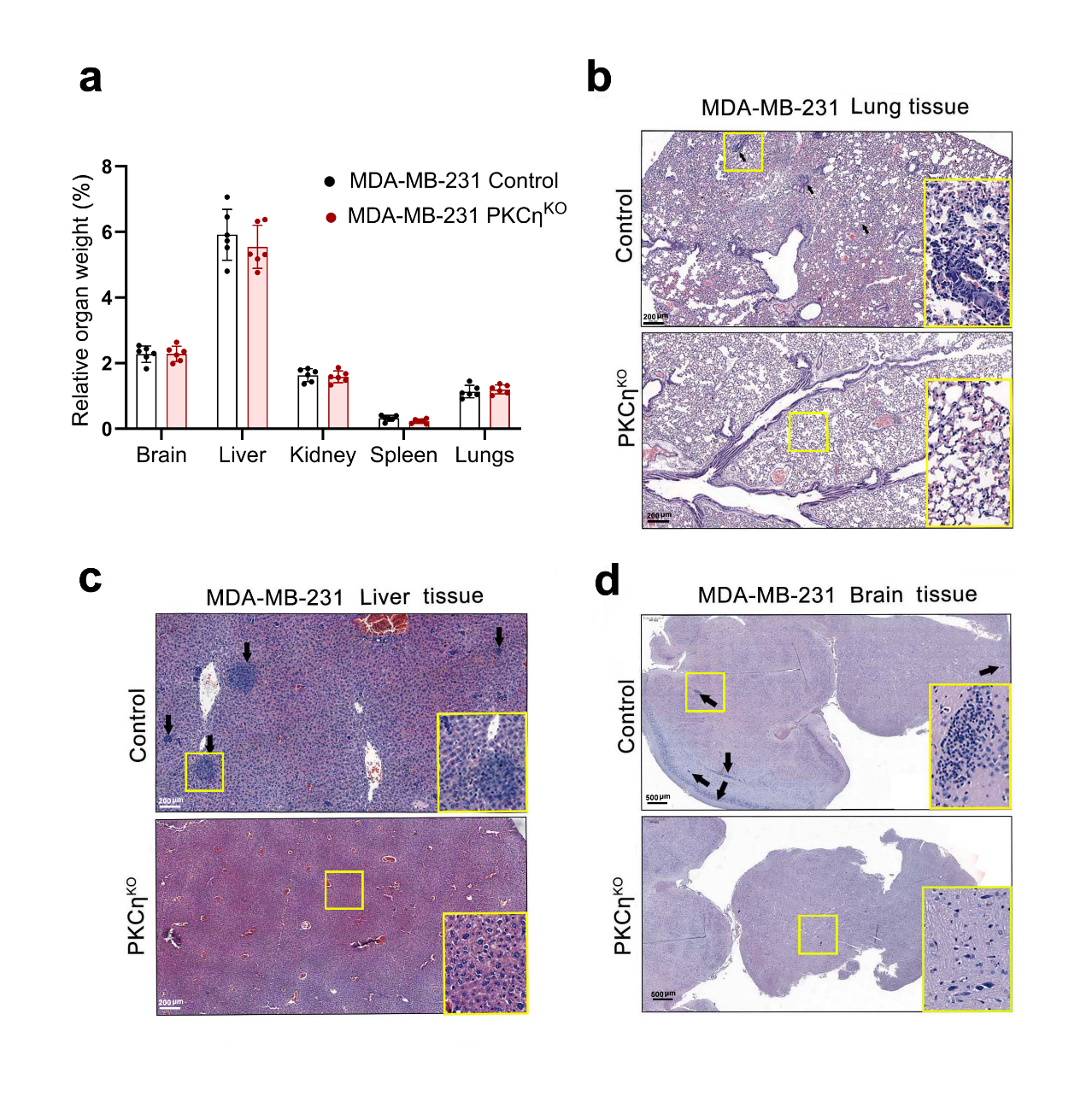


**Supplementary Fig. 15. Orthotopic xenografts of MDA-MB-231 PKCη^KO^ cells in NSG mice showing reduced metastasis.**

**a**, Graphical representation of relative organ weights (brain, liver, kidney, spleen, and lung) of MDA-MB-231 control and PKCη^KO^ xenografted NSG mice. **b**, H&E images of the lungs collected at the end of the experiment showing metastatic lung nodules (indicated by a black arrow) in control MDA-MB-231 xenografted NSG mice. The scale bar indicates 200 μm. **c,** Liver tissue (CRISPR control and MDA-MB-231 PKCη^KO^) sections were examined by H&E staining and observed under a light microscope. Microscopic liver metastatic lesions are identified (black arrows). Micronodules with multiple spherical metastases were observed on the surface of the liver. The scale bar indicates 200 μm. **d**, Histopathological images of the brain collected at the end of the experiment demonstrating micrometastasis (indicated by a black arrow) in control MDA-MB-231 xenografted NSG mice. The scale bar indicates 500 μm. Metastatic nodules with multiple spherical micrometastasis were observed in the lungs, liver, and brain.

**Figure S16**


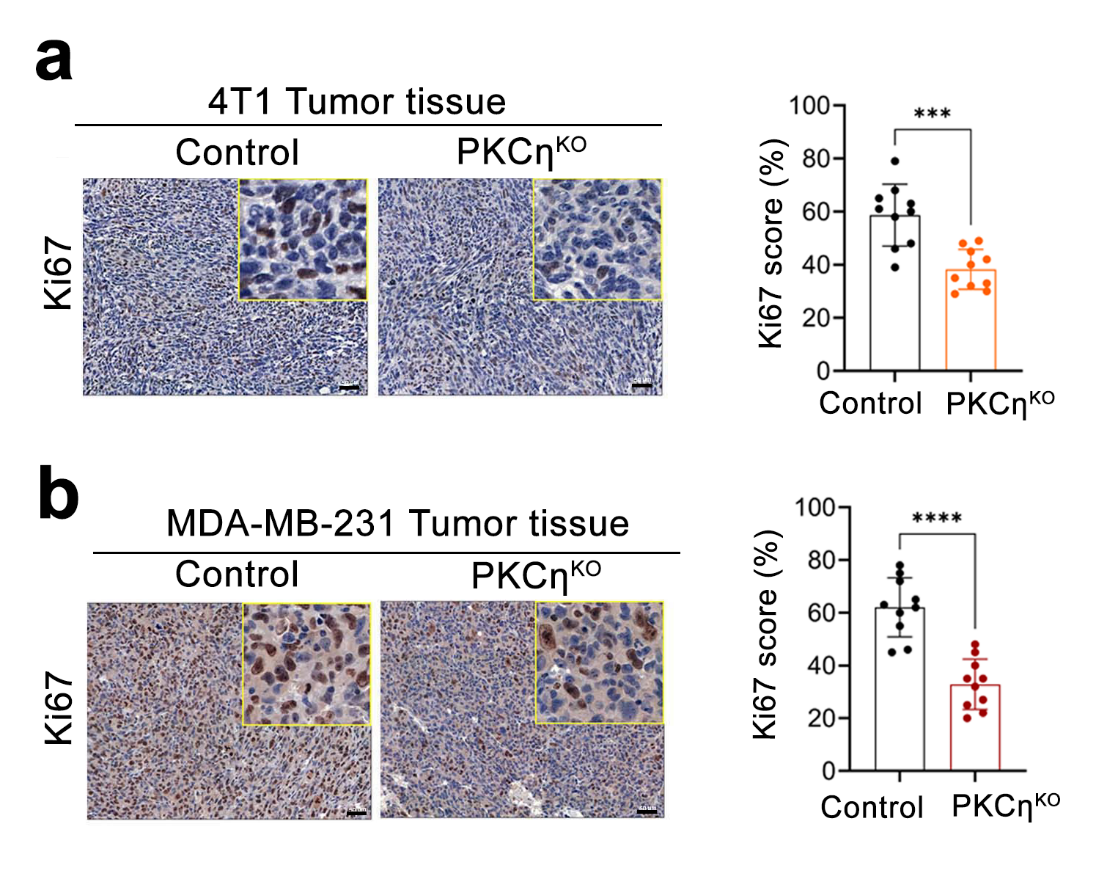


**Supplementary Fig. 16. TNBC PKCη^KO^ cells exhibited reduced levels of the proliferation marker (Ki67) in the primary tumor tissue.**

Primary tumor tissues exhibited elevated expression of the proliferation marker Ki67 compared to PKCη^KO^ tumor tissues in both (**a**) 4T1 and (**b**) MDA-MB-231 xenograft NSG mice models. The scale bar indicates 50 μm. Significance was determined using two-way ANOVA. *P < 0.05, **P < 0.01, ***P < 0.001, ****p<0.0001.

**Figure S17**


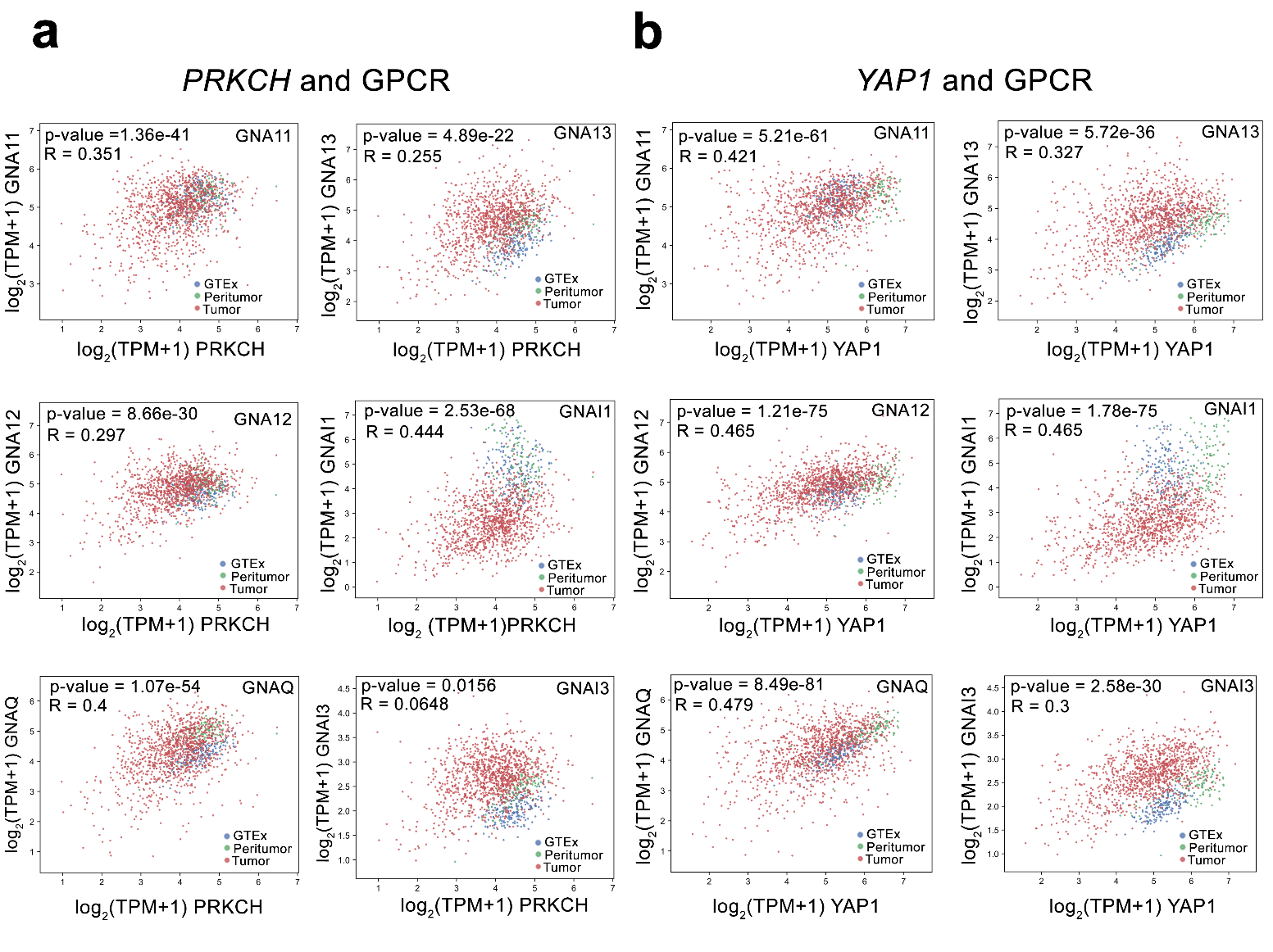


**Supplementary Fig. 17. The expression levels of *PRKCH* and *YAP1* were positively correlated with GPCRs expression.**

**a** and **b**, GPCR coupled with Gq/11 (GNAQ/GNA11), G12/13 (GNA12 and GNA13), and Gi (GNAI1) typically stimulate YAP/TAZ activation and its nucslear translocation. Analysis of TNBC patient samples showed that the RNA expression levels of G proteins, including *GNAQ/GNA11, GNA12/GNA13,* and *GNAI1/GNAI3*, were positively correlated with increased expression levels of *PRKCH* and *YAP1*. Correlation analysis was performed via GEPIA3 with the TCGA-BRCA datasets (TCGA-Tumor, TCGA-Peritumor, and Genotype-Tissue Expression (GTEx)-normal reference).

**Figure S18**


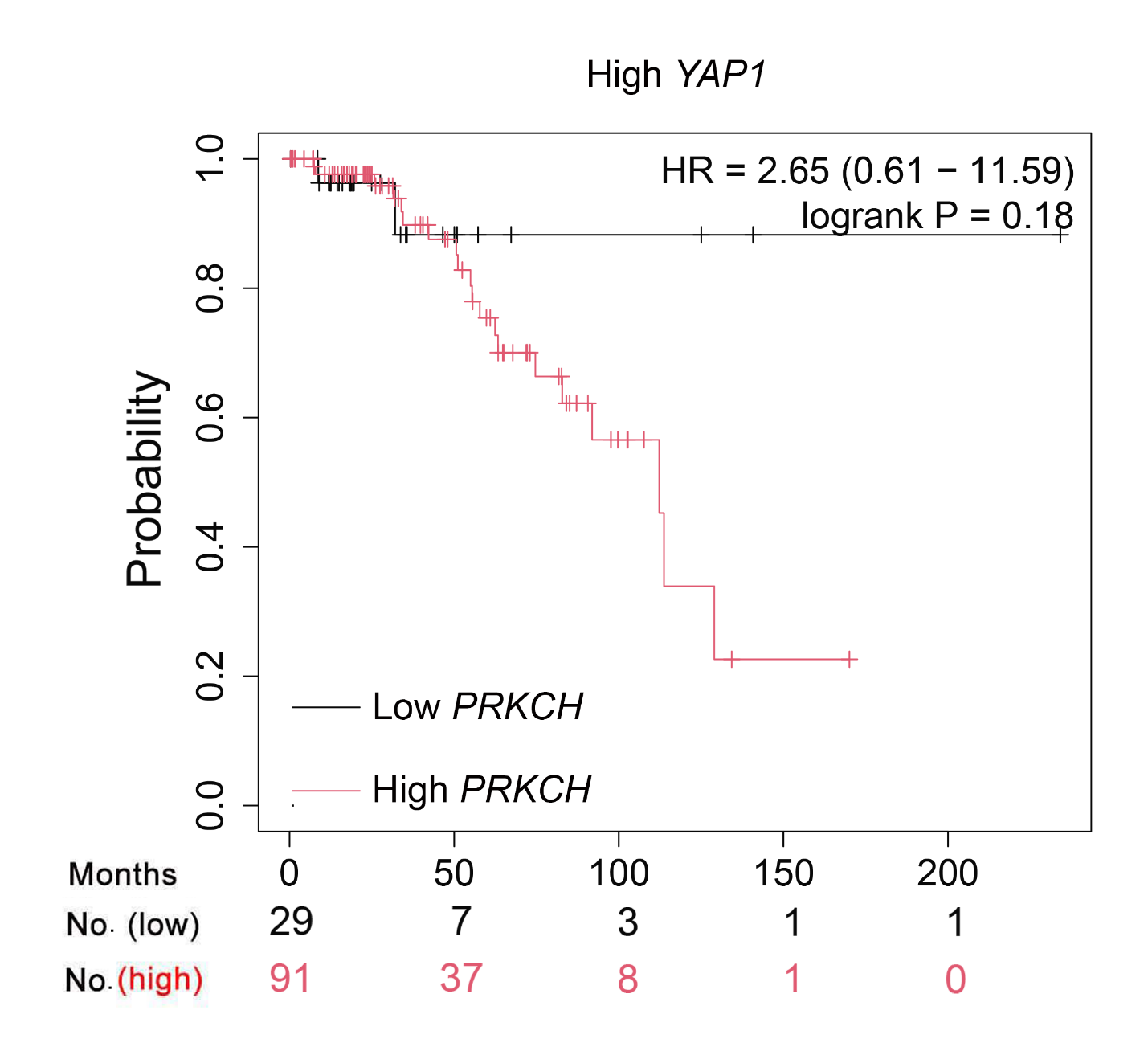


**Supplementary Fig. 18.** **Prognostic value of TNBC tumors exhibiting high YAP expression with either low or high PKCη expression.**

The survival graph demonstrates that patients with high YAP and PKCη expression exhibit worse overall survival than those with high YAP and low PKCη. The calculated hazard ratio (HR) for the high YAP/high PKCη group versus the high YAP/low PKCη group was 2.65, indicating that the risk of adverse events was 2.65 times higher in the high PKCη subgroup.

**Figure S19**


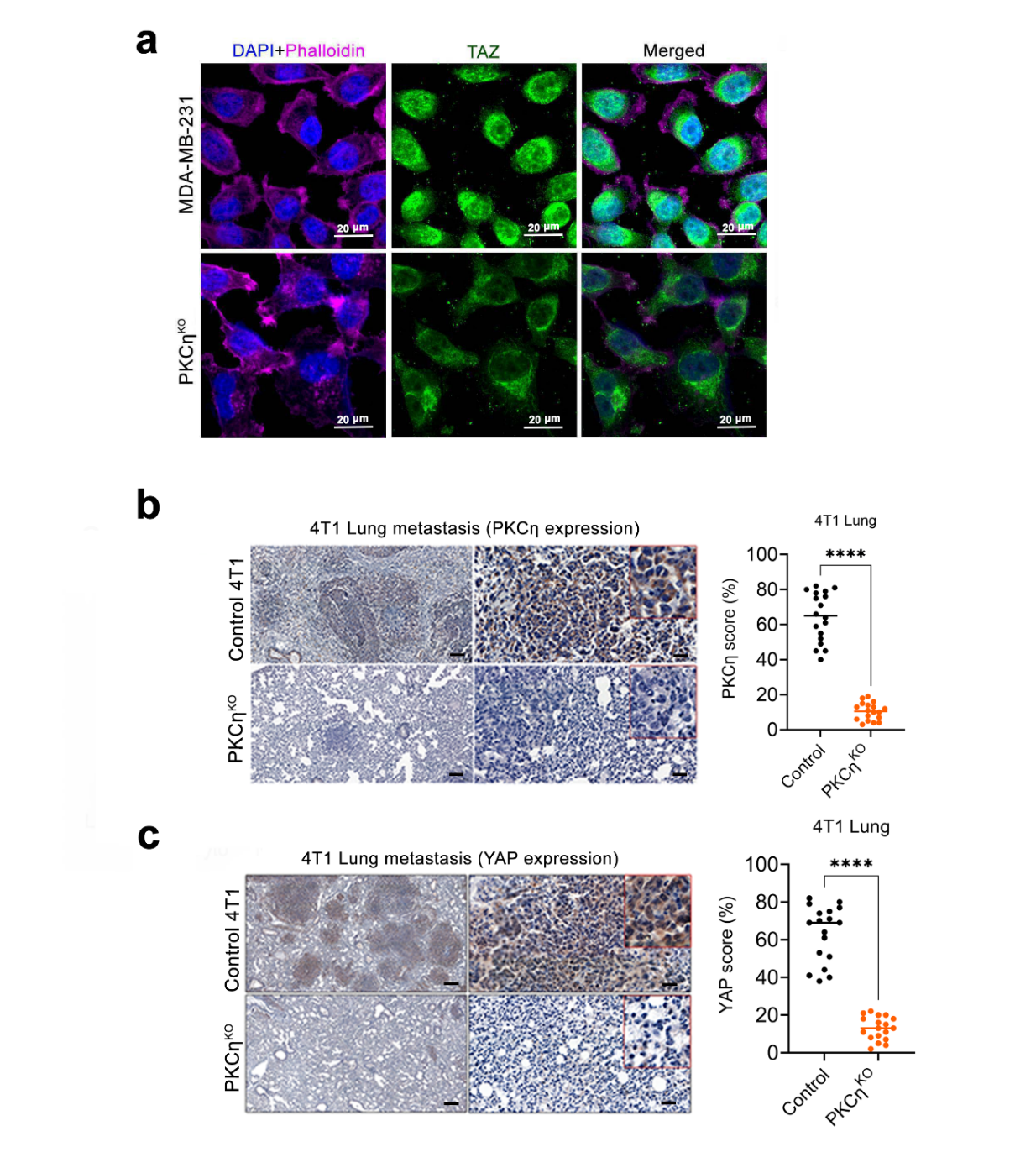


**Supplementary Fig. 19. PKCη stabilizes YAP/TAZ protein levels in TNBC cells**.

**a,** Immunofluorescence images of TAZ nuclear and cytoplasmic expression in control and PKCη^KO^ MDA-MB-231 cells. TAZ was stained green, phalloidin was stained pink, and the cell nuclei were counterstained with DAPI (blue). **b, c,** IHC analysis revealed metastatic nodules displaying high expression levels of PKCη and YAP in the lungs of 4T1 xenografted mouse tumors. The quantification of PKCη and YAP expression is presented herein. The scale bar indicates 100 μm.

**Figure S20**


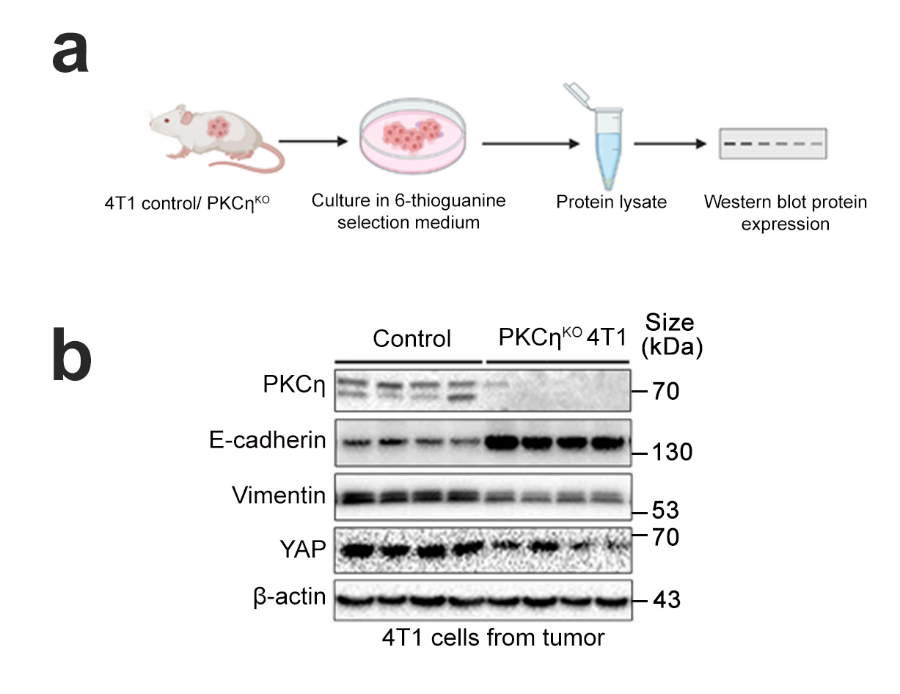


**Supplementary Fig. 20. 4T1 PKCη^KO^ cell xenograft tumors exhibited reduced levels of EMT and YAP expression.**

**a,** 4T1 cells from primary tumors of xenografted in NSG mice were cultured using 60 µM 6-thioguanine (4T1 cells are resistant to 6-thioguanine). **b,** Isolated 4T1 cells were lysed and analyzed for the expression of PKCη, E-cadherin, Vimentin, and YAP by immunoblotting.

**Figure S21**


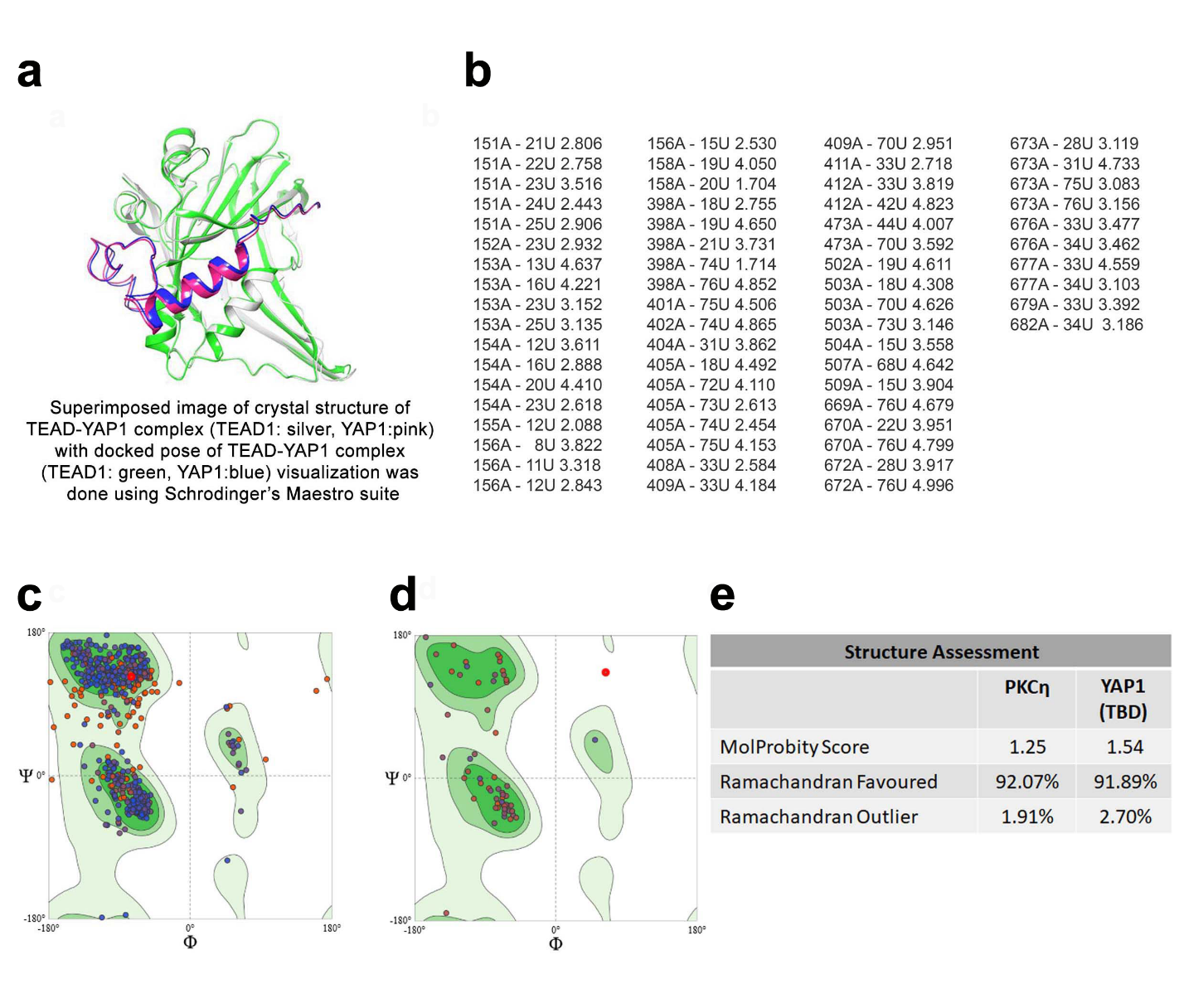


**Supplementary Fig. 21. Protein-protein docking of YAP and PKCη.**

**a,** To ensure that the protein–protein docking protocol used was reliable, the crystal structure chains of the TEAD1-YAP 1 complex (PDB ID: 3KYS) were extracted and docked using the HDOCK Server (http://hdock.phys.hust.edu.cn). The superimposition of crystal structure and docked pose resulted in a Root Mean Square Deviation (RMSD) value for C-Alpha and Protein Backbone atoms of 0.9 Å and 1.7 Å, which was well within the acceptable range of 2 Å, validating the docking algorithm. The docking and superimposition results are presented. **b,** Receptor-ligand interface receptor pairs and their respective distances (Å): A=PKCη and U=YAP (Fig. 5c). **c-e,** Ramachandran plot prediction and structural assessment of YAP1 and PKCη.

**Figure S22**


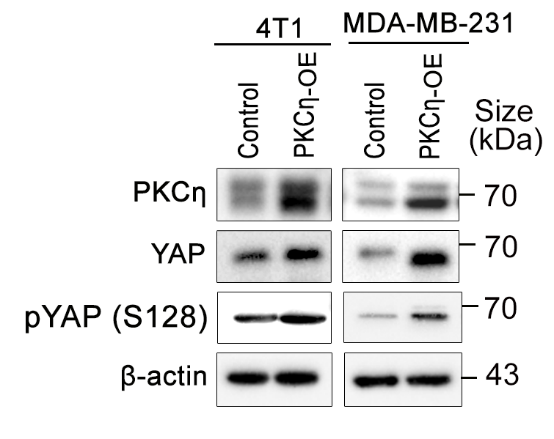


**Supplementary Fig. 22. Overexpression of PKCη (PKCη-OE) in 4T1 and MDA-MB-231 cells is associated with elevated levels of YAP and its phosphorylation at S128.**

**a,** Overexpression of PKCη (PKCη-OE) in 4T1 and MDA-MB-231 cells showed increased levels of YAP and its phosphorylation at S128 (pYAP S128).

**Figure S23**


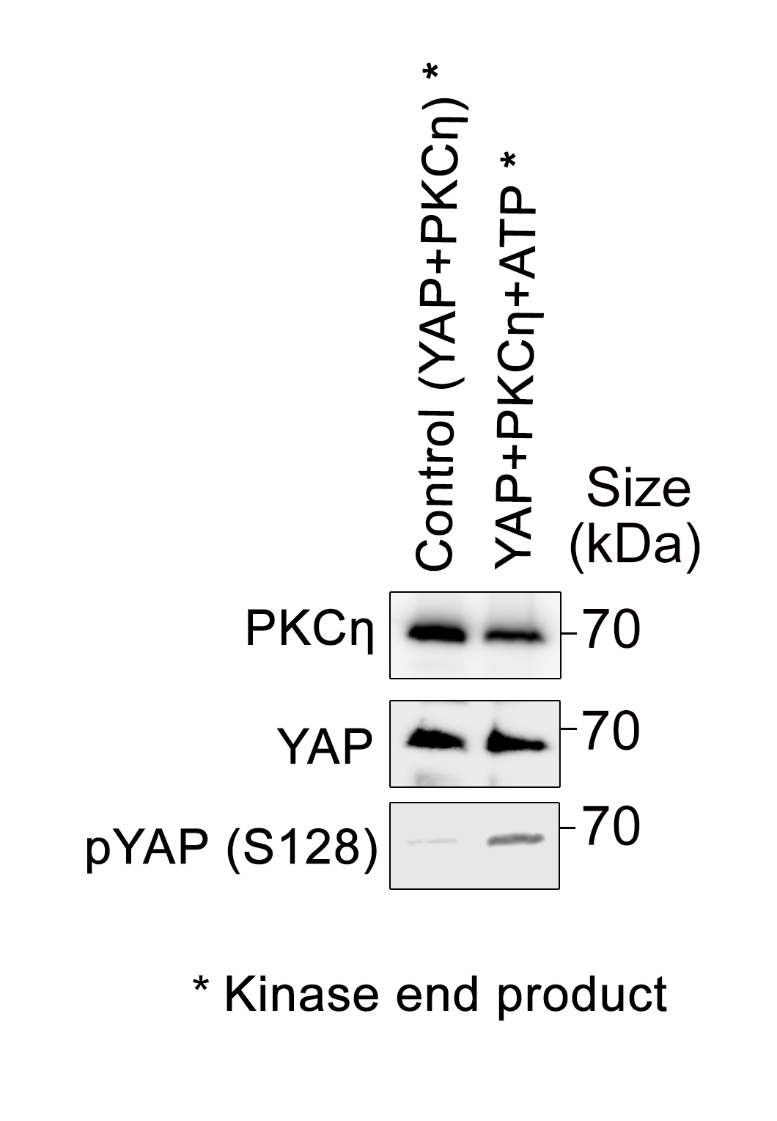


**Supplementary Fig. 23. PKCη-mediated phosphorylation of YAP at S128.**

After the kinase assay with recombinant proteins YAP and PKCη, the kinase reaction products were analyzed by western blotting. The results showed that YAP S128 was phosphorylated after the kinase assay.

**Figure S24**


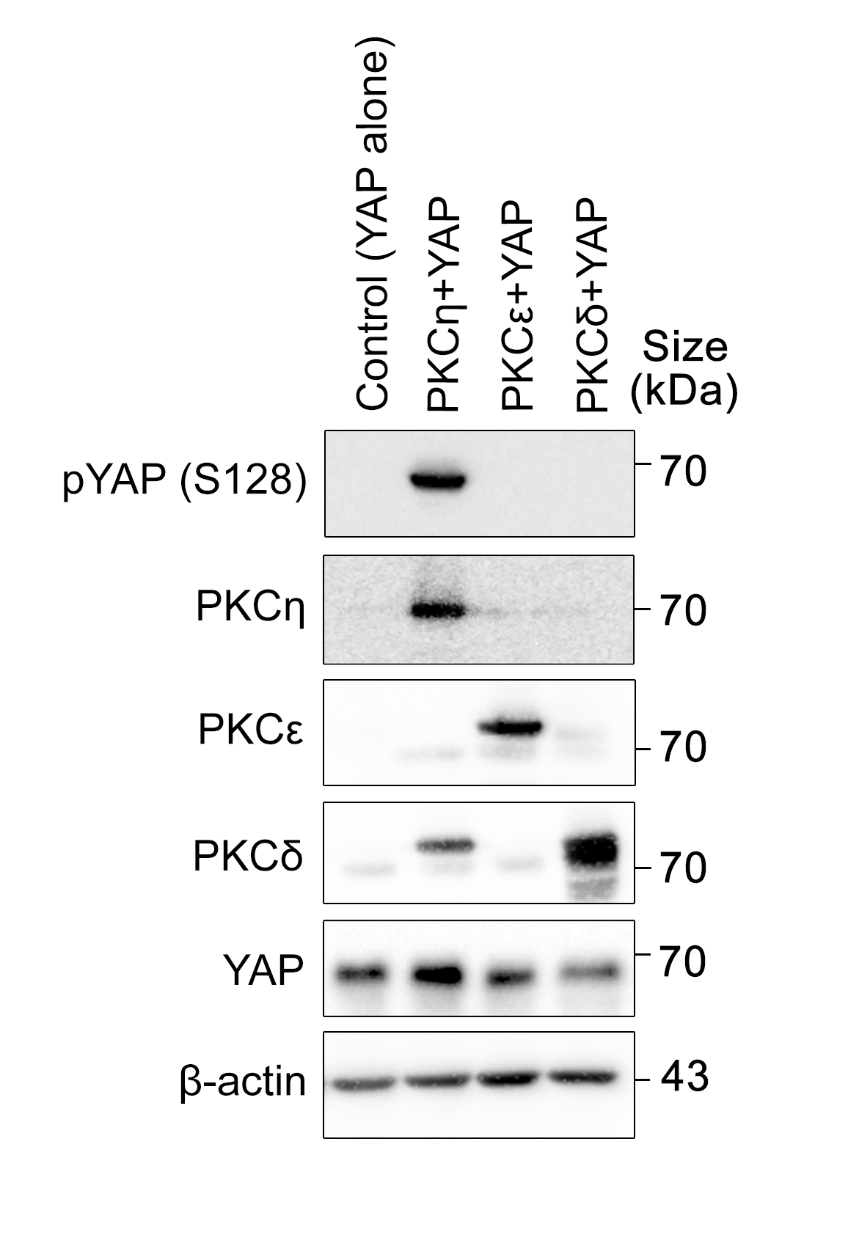


**Supplementary Fig. 24. YAP phosphorylation at serine 128 was specifically induced by PKCη.**

HEK293FT cells were transfected with YAP alone or YAP together with PKCη, PKCε, and PKCδ. Western blot analysis showed phosphorylation of YAP at Ser128 only in the presence of PKCη. YAP Ser128 phosphorylation was not detected upon expression of PKCε or PKCδ.

**Figure S25**


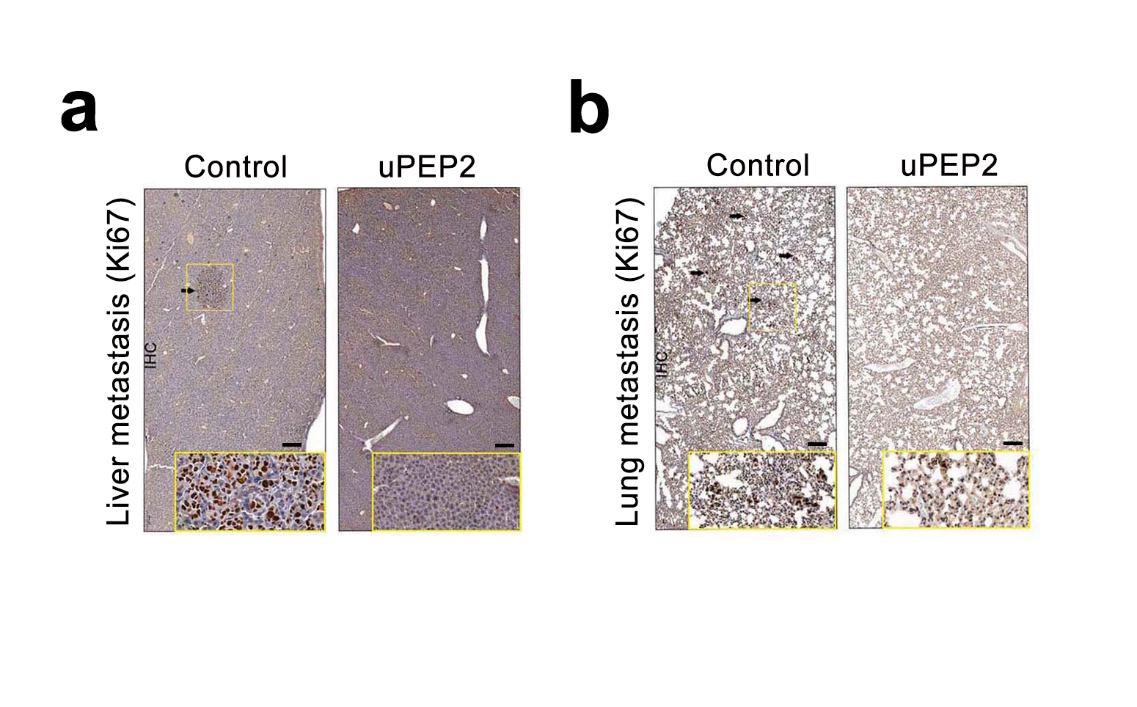


**Supplementary Fig. 25. Treatment of MDA-MB-231 xenografts with uORF-encoded micropeptide (uPEP2) reduced cell proliferation.**

**a**, Liver tissue harvested from uPEP2-treated MDA-MB-231 xenografted NSG mice showed reduced expression of the proliferation marker Ki67. **b**, Lung tissue obtained from the same mice also showed decreased expression of Ki67. The scale bar indicates 200 μm.

**Figure S26**


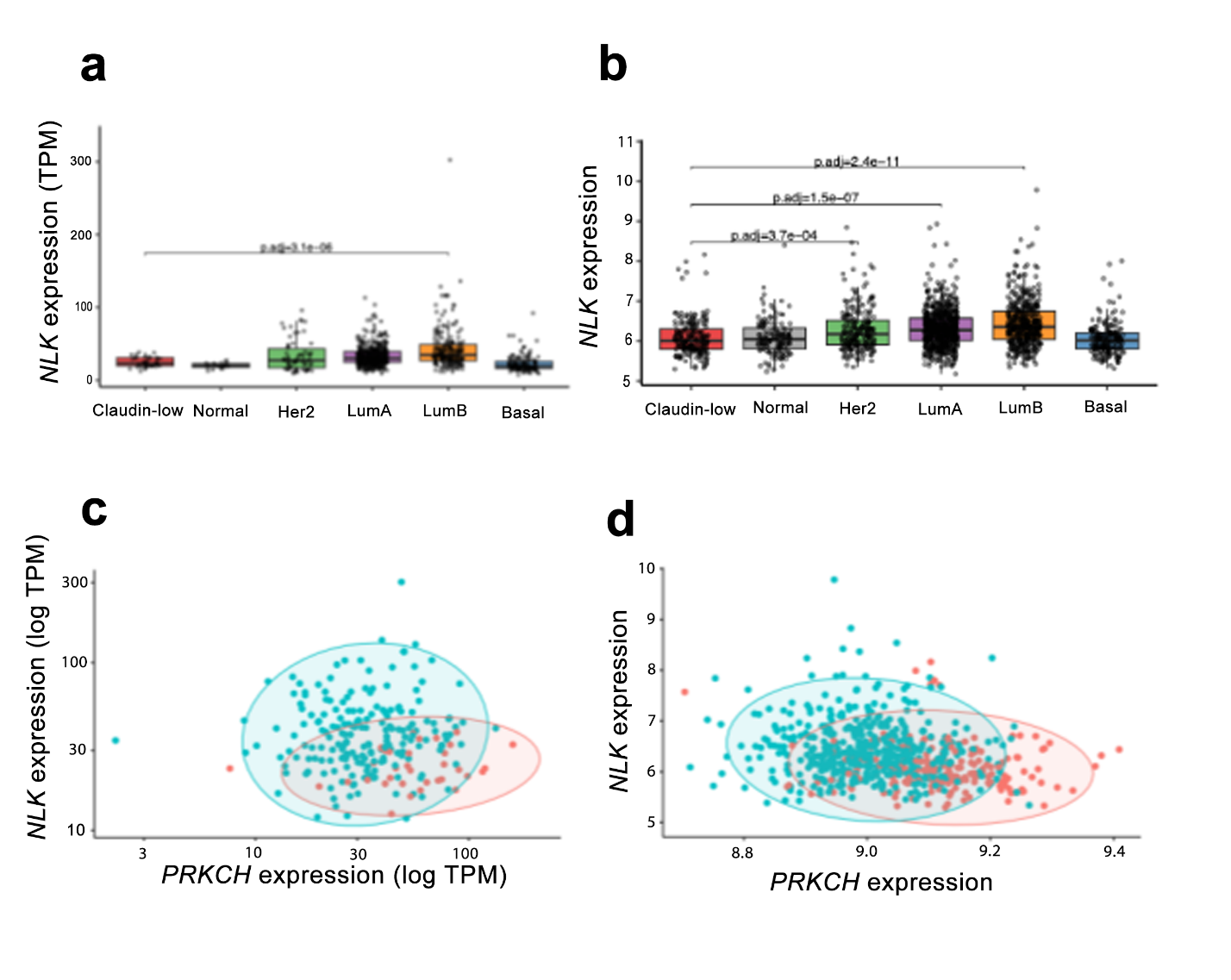


**Supplementary Fig. 26.** **Expression pattern and correlation analysis of *NLK* and *PRKCH* across BC subtypes.**

**a-b**, *NLK* expression levels across intrinsic molecular subtypes in METABRIC and TCGA datasets, respectively, showed significantly lower *NLK* expression in claudin-low tumors compared to other BC subtypes (Her2, Luminal A, Luminal B), whereas *PRKCH* was highly expressed in the claudin-low subtype (Supp Fig. 1). **c-d,** Scatter plots illustrating the correlation between *NLK* and *PRKCH* mRNA expression levels in claudin-low (red) and luminal B (blue) subtypes in METABRIC (c) and TCGA (d).

**Supplementary Tables**

**Table S1**. Differentially Expressed Genes (DEGs) of PKCη-high tumors (top 80**%).**

Table S2.

Differentially Expressed Genes (DEGs) of PKCη-low tumors (bottom 20%).

Table S3.

Differentially Expressed Genes (DEGs) of PKCη-high tumors (bottom 25%).

Table S4.

Differentially Expressed Genes (DEGs) in PKCη-low tumors (bottom 10%).

Table S5.

Immunohistochemical quantification of human TNBC samples.

| Mouse sgRNA for *PRKCH* | Fwd: 5’-CACCGCACAAAGTGGTCATAACCCAG-3’ |
| --- | --- |
| Mouse scrambled sgRNA | 5’-CACCGCACTACCAGAGCTAACTCA-3’ |
| Human sgRNA for *PRKCH* | Fwd: 5’-CACCGCCACGCTCACCGTCAGATAG-3’ |
| Human scrambled sgRNA | 5'-CACCGATCGTTTCCGCTTAACGGCG-3' |
| **Sequencing primers** | |
| Mouse | Fwd: 5- ACCTGACGGTGAGCGTAGAC-3’  Rev: 5’- CTGTCGCGTCAACAGTGC-3’ |
| Human | Fwd: 5’- ATGGCTATTTGAGGGTCCGC-3’  Rev: 5’- CGTTAGCGCAAAACTCCTCG-3’ |

Table S6. Guide RNA (gRNA) sequences targeting the PRKCH gene.

| No | Gene name | qPCR primer sequence |
| --- | --- | --- |
| 1 | *PTEN* | Forward primer (5' → 3'): CCCACCACAGCTAGAACTTATC  Reverse primer (5' → 3'): TCGTCCCTTTCCAGCTTTAC |
| 2 | *AXL* | Forward primer (5' → 3'): GGATGAACAGGATGACTGGATAG  Reverse primer (5' → 3'): AAGGTCTGATGTCCCAGAAAC |
| 3 | *IGFBP3* | Forward primer (5' → 3'): GCGCTACAAAGTTGACTACGA  Reverse primer (5' → 3'): TCTACGGCAGGGACCATATT |
| 4 | *TEAD1* | Forward primer (5' → 3'): CTGGTCTCTGGGCATTCATT  Reverse primer (5' → 3'): CAGGTAACTTGGGCCATTCT |
| 5 | *CYR61* | Forward primer (5' → 3'): TCGCATCCTATACAACCCTTTAC  Reverse primer (5' → 3'): GATACCAGTTCCACAGGTCTTT |
| 6 | *PRKCH* | Forward primer (5' → 3'): TTGGAAGGGACGGTCGG  Reverse primer (5' → 3'): GTTAGCGCAAAACTCCTCGT |
| 7 | *TBP* | Forward primer (5' → 3'): CTTCGGAGAGTTCTGGGATTG  Reverse primer (5' → 3'): CACGAAGTGCAATGGTCTTTAG |

**Table S7.** **PCR Primer Sequences**

**Reference**

1 Curtis, C. et al*.* The genomic and transcriptomic architecture of 2,000 breast tumours reveals novel subgroups. *Nature* **486**, 346–352 (2012).

2 Leek, J. T., Johnson, W. E., Parker, H. S., Jaffe, A. E. & Storey, J. D. The sva package for removing batch effects and other unwanted variation in high-throughput experiments. *Bioinformatics* **28**, 882–883 (2012).

3 Chen, T. XGBoost: A Scalable Tree Boosting System. *Cornell University* (2016).

4 Lehmann, B. D. et al. Multi-omics analysis identifies therapeutic vulnerabilities in triple-negative breast cancer subtypes. *Nat. Commun.* **12**, 6276 (2021).

5 Agro, L. & O’Brien, C. A. In vitro and in vivo limiting dilution assay for colorectal cancer. *Bio Protoc.* **5**, e1659–e1659 (2015).

6 Nagai-Singer, M. A. et al*.* Using computer-based image analysis to improve quantification of lung metastasis in the 4T1 breast cancer model. *J. Vis. Exp.* **164**, e61805 (2020).

7 Jagadeeshan, S. et al. Mutated HRAS activates YAP1–AXL signaling to drive metastasis of head and neck cancer. *Cancer Res.* **83**, 1031–1047 (2023).

8 Yan, Y., Tao, H., He, J. & Huang, S.-Y. The HDOCK server for integrated protein–protein docking. *Nat. Protoc.* **15**, 1829–1852 (2020).
